# Synthesis and Cytotoxicities of Royleanone Derivatives

**DOI:** 10.1007/s13659-018-0173-y

**Published:** 2018-06-16

**Authors:** Cheng-Ji Li, Fan Xia, Rong Wu, Hong-Sheng Tan, Hong-Xi Xu, Gang Xu, Hong-Bo Qin

**Affiliations:** 1 State Key Laboratory of Phytochemistry and Plant Resources in West China, Kunming Institute of Botany, Chinese Academy of Sciences, and Yunnan Key Laboratory of Natural Medicinal Chemistry, Kunming, 650201 People’s Republic of China; 2 Shanghai University of Traditional Chinese Medicine, Shanghai, People’s Republic of China; 3 University of Chinese Academy of Sciences, Beijing, 100049 People’s Republic of China

**Keywords:** Royleanones, Cytotoxicity, *para* benzoquinone

## Abstract

Carnosic acid was used as starting material to synthesize royleanone derivatives featured C11–C14 para quinone. The importance of C-20 group of royleanone derivatives was verified by the cytotoxicity assay of royleanonic acid, miltionone I and deoxyneocrptotanshinone. Following our synthetic route, 15 amide derivatives were synthesized and 8 compounds exhibited moderate cytotoxic activities against three human cancer lines in vitro.

**Electronic supplementary material:**

The online version of this article (10.1007/s13659-018-0173-y) contains supplementary material, which is available to authorized users.

## Introduction


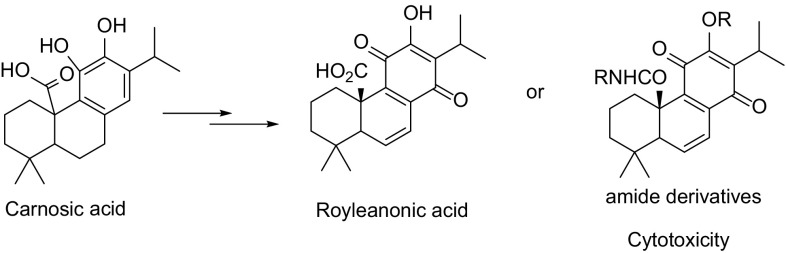
Royleanones, possessing a characteristic 11,14-para benzoquinone 12-hydroxy abietane skeleton, were first isolated by Handa’s group in 1945 from the roots of *Inula royleana* as yellow pigment [1]. Their chemical structures have been explicitly established for the first time in 1962 by Edwards by synthesis [2]. Royleanones have shown various pharmacological activities, including antitumor, anti-oxidant, antidiabetic [3–5]. These diterpenoids attracted extensive attentions among synthetic chemists and pharmacologists [6].

**Fig. 1:**
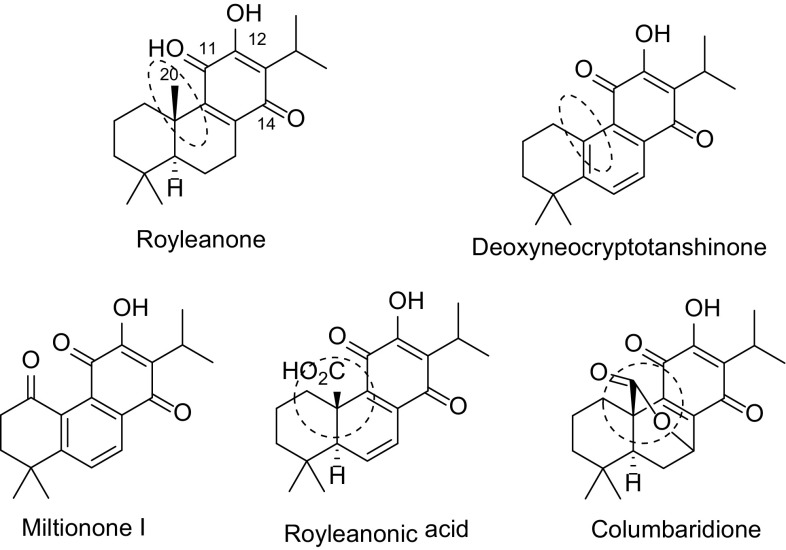
Structures of royleanone derivatives

Our research interests are largely related to the antitumor molecules, especially tricyclic terpenoids derivatives [7–12]. Tanshinones have the functional group of 11,12 ortho benzoquinone, which can serve as a Michael acceptor at C-14 to cellular nucleophiles such as DNA, RNA, protein and GSH [13]. Hence, tanshinones are potent cytotoxic. Considering the fact that *para* benzoquinone moiety in royleanones could not act as Michael acceptor, the cytotoxic activity of royleanones would be weak. In fact, royleanones has IC_50_ of 32.5 µM to MIAPaCa-2 cell line and deoxyneocryptotanshinone was inactive (IC_50_ > 100 µM) to the same cell line [4]. Royleanonic acid was also inactive to K562 human leukemia cell line. However, columbaridione showed potent cytotoxic activity to K562 at 10 µM (Fig. 1) [14]. The difference might attribute to the free acid group and there must be unsolved mechanism other than the Michael acceptor conception because the C-14 was blocked in royleanones. Studies on the structure–activity relationship of royleanon derivatives are rare. Therefore, investigations on the *para* benzoquinone derivatives might lead to more potent analogues than columbaridione.

**Scheme 1:**
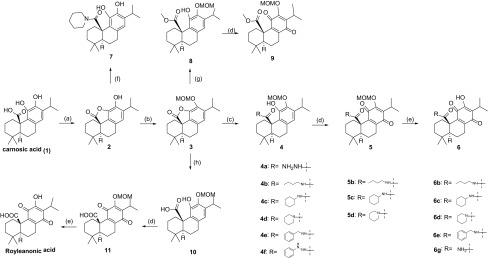
Synthetic route of target compounds. Reagents and conditions: **a** EDCI, DMAP, DCM, rt, 3 h; 95%, **b** MOMCl, DIPEA, DCM, rt, 12 h; 88%, **c** Amine or hydrazine, THF, rt, 0.5–5 h; 90%, **d** *m*-CPBA, NaHCO_3_, DCM, rt, 16 h; 45%, **e** HCl, MeOH, rt, 12 h; 60%, **f** piperidine, THF, rt, 0.5 h; 65%, **g** MeONa, MeOH, rt, 12 h; 80%, **h** LiOH, THF, H_2_O, rt, 2 h, 60%

We focused on the C-20 carbonic acid group of royleanonic acid. It could be transformed into amide derivative because the C-20 is important to maintain the cytotoxicity from above analysis. Therefore, we chose carnosic acid as starting material and then transformed into *para* benzoquinone. As a result, 15 new amide derivatives were synthesized (Scheme 1). Subsequently, these compounds were tested against three human cancer cell lines (HepG2, MCF 7 and A549). Amide derivatives showed potent antitumor activities.

## Results and Discussions

To verify the hypothesis of the importance of C-20, we synthesized royleanonic acid as indicated in Scheme 1. Started from carnosic acid (**1**), intramolecular esterification occurred to afford lactone **2** with DCC. The 12-hydroxy group was then protected as MOMO ether. After hydrolysis, the resulting free phenol **10** was oxidized into *para* benzoquinone **11**. Liberation of MOMO ether by HCl afforded the desired royleanonic acid.

**Scheme 2:**
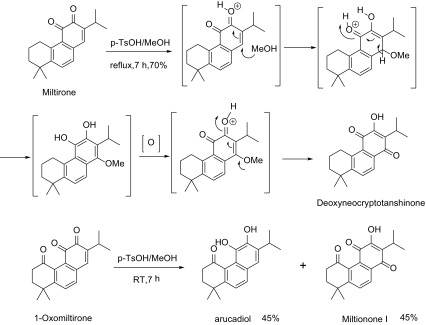
Synthetic route to deoxyneocryptanshinone and miltionone I

Next, its derivatives without C-20 group, such as miltionone I and deoxyneocryptanshinone, were also prepared. When miltirone was treated with *p*-TsOH in MeOH, Deoxyneocrptanshinone was obtained in 70% yield (Scheme 2). The possible mechanism involved the Michael addition of MeOH to miltirone at C-14, followed by oxidation and isomerization of vinyl ether into *para* benzoquinone. Similarly,

miltionone I was obtained from 1-oxomiltirone under the same condition. Meanwhile, arucadiol was also isolated which implied the reduction of 1-oxomiltirone. Although its mechanism is not clear yet, this transformation is different with known method of deprotection of *ortho* dimethoxy group [15].

The three diterpenoids were subjected to antitumor assay. The results were collected in Table 1. As can be clearly seen in Table 1, royleanonic acid exhibited more potent antitumor activity, which proved the importance of C-20 group. Obviously, the existence of C-20 group is important.

**Table 1:** Cytotoxicities of compounds against three cancer cell lines (IC_50_ μM)

Compounds	Hep G2	MCF 7	A549
Royleanonic acid	13.46	25.91	26.80
Miltionone I	> 100	86.82	> 100
Deoxyneocryptanshinone	53.17	78.96	74.28
**2**	11.12	4.24	18.04
**4a**	41.63	11.87	> 100
**4b**	35.54	50.46	39.07
**4c**	67.56	65.76	64.56
**4d**	72.29	49.53	48.39
**4e**	52.72	70.04	74.81
**4f**	> 100	> 100	> 100
**5b**	7.96	19.92	37.88
**5c**	44.75	> 100	> 100
**5d**	7.28	17.81	32.87
**6b**	3.93	14.90	16.48
**6c**	6.18	8.37	19.11
**6d**	5.74	10.53	17.76
**6e**	7.8	13.16	23.74
**6** **g**	28.35	82.78	> 100
**7**	1.02	2.03	24.26
**9**	24.90	26.84	62.70
STS^a^	0.04	0.10	0.01

^a^Positive control

Hence, following this route in Scheme 1, several amide derivatives were synthesized using amines to open lactone.

All the synthesized compounds were evaluated for cytotoxicity against three human cancer cell lines (HepG2, MCF 7 and A549). Eight royleanone derivatives showed potent cytotoxicity against three human cancer cell lines (IC_50_ < 10 μM) (Table 1). It is noteworthy that compound **7** had a significant cytotoxicity toward Hep G2 and MCF 7 human cancer lines in vitro (IC_50_ 1.02–2.03 μM).

Our experiments demonstrated that, for the first time, modification of orthoquinone into *para* benzoquinone have shown antitumor activity. Block of C-14 conjugate addition site did not lead to the drop of cytotoxicity (**5b, 5d, 6b–6e**). These results may attribute to mechanism other than Michael addition of DNA, RNA etc. [13]. The existence of C-20 amide functional group is important to the cytotoxicity when compared with deoxyneocryptanshinone and miltionone I. Cyclic secondary amine **7** gave the best result. All derivatives are active towards HpeG2 cell line. These findings may serve as new clues for discovering antitumor molecules with new structure scaffold.

## Electronic supplementary material

Below is the link to the electronic supplementary material.

Supplementary materials such as experimental procedures, MS and NMR spectra of the synthesized compounds are available in the online version of this article free of charge. Supplementary material 1 (DOC 5423 kb)
