# Supplementary material for: Synthesis and Cytotoxicities of Royleanone Derivatives
Source: Nat Prod Bioprospect. 2018 Jun 16;8(6):453–6. doi: 10.1007/s13659-018-0173-y (PMC6224808; doi:10.1007/s13659-018-0173-y)
Supplement: Supplementary file 1 — Supplementary materials such as experimental procedures, MS and NMR spectra of the synthesized compounds are available in the online version of this article free of charge. Supplementary material 1 (DOC 5423 kb) [file 13659_2018_173_MOESM1_ESM.doc]

Supporting Information

**Synthesis and Cytotoxicities of Royleanone derivatives**

Cheng-Ji Li,^a,c,†^ Fan Xia,^a,c,†^ Rong Wu,^b^ Hong-Sheng Tan,^b^ Hong-Xi Xu,b,[[1]](#footnote-2)* Gang Xu,a,[[2]](#footnote-3)* and Hong-Bo Qina,[[3]](#footnote-4)*

*a State Key Laboratory of Phytochemistry and Plant Resources in West China, Kunming Institute of Botany, Chinese Academy of Sciences, and Yunnan Key Laboratory of Natural Medicinal Chemistry, Kunming 650201, P. R. China*

*b Shanghai University of Traditional Chinese Medicin, Shanghai*

*c University of Chinese Academy of Sciences, Beijing 100049, P. R. China*

**Table of Content**

Experimental methods of cytotoxic activity**.**

General procedure for the synthesis of compounds **2, 3, 4a-4f, 5b-5d, 9, 11, 6b-6e, 6g, 12, 7, 8, 10.**

Datas of ^1^H and ^13^C NMR of **2**, **3**, **4a-4f**, **5b-5d**, **6b-6g**, **7**, **8**, **9**, **10**, **11**, **12**, **deoxyneocytotanshinone**, **arucadiol**, and **miltinone I**.

**General:** All reactions were performed in sealed tube containing a Teflon-coated stir bar. All reagents were obtained from commercial sources and used without further purifications. ^1^H and ^13^C NMR spectra were recorded in a Bruker 300 MHz and 500 MHz NMR spectrometer, respectively, with tetramethylsilane as an internal reference. Low- and high resolution mass spectra were obtained in the EI (70 eV) mode. Flash column chromatography on silica gel (200–300 mesh) was used for the routine purification of reaction products. The column output was monitored by TLC on silica gel (100–200 mesh) precoated on glass plates (10×50 cm), and spots were visualized by UV light at 254 nm.

**Cytotoxic activity:**

1. **Abbreviations and definitions**

mg milligram

mL milliliter

min minute

SD Standard Deviation

SOP Standard Operating Procedure

µg microgram

µl microliter

ATCC American Type Culture Collection

1. **Materials and Assay Condition**

（1）Materials:

- MEM (from Invitrogen, Cat. No. 11095-080; Lot. No.1868728)
- MEM NEAA (from Invitrogen, Cat. No.11140-050; Lot. No.1713032)
- F-12 K (from Invitrogen, Cat. No.211217-022; Lot. No.1854888)
- FBS (from Biological Industries, Cat. No. 04-002-1A, Lot. No. 1609758)
- 0.25%Trypsine-EDTA (from Invitrogen, Cat. No. 25200-072, Lot. No. 1806021)
- Distilled Water (from HyClone, Cat. No. SH30529.03, Lot. No. AB10190967)
- Penicillin/Streptomycin (from HyClone, Cat. No.SV30010, Lot. No. J1660032)
- DMSO (from Sigma, Cat. No. 276855-1L. Lot. No STBD8882V)
- 384-well plate, white wall, tissue culture treated (from Corning, Cat. No. CLS3707; Lot. No. 14815045)
- CellTiter Glo assay kit (from Promega, Cat. No. G7573 Lot. No. 00000249676)
- Staurosporine (from sigma, Cat. No. S4400-1MG, Lot. No. 063M4112V)
- Compound: all compounds from ****

（2）Assay condition:

| **Cell Line** | **Vendor** | **Cat#** | **Description** | **Growth properties** | **Complete medium** | **Seeding Density_for 72hr compound incubation, 384-well plate** |
| --- | --- | --- | --- | --- | --- | --- |
| A549 | ATCC | CCL-185 | lung | adherent | F-12K+10%FBS | 500 |
| MCF-7 | ATCC | HTB-22 | breast | adherent | EMEM+0.01mg/ml bovine insulin+ 10%FBS | 1000 |
| Hep G2 | ATCC | HB-8065 | liver | adherent | EMEM+10%FBS | 1250 |

1. **Experimental methods**

（1）Cell seeding

1. Prepare complete medium: Add FBS and appropriate additives according to the information sheet provided by the provider. Mix gently.
2. Check the cell name and complete medium and passage number marked on the flask. For attached cell lines, refer to c to k.
3. Remove and discard culture medium using a vacuum pump.
4. Briefly rinse the cell layer with 0.25% (w/v) Trypsin-0.038% (w/v) EDTA solution to remove all traces of serum that contains trypsin inhibitor.
5. Add 3.0 ml of Trypsin-EDTA solution to flask and observe cells under an inverted microscope until cell layer is dispersed.
6. Add 8.0 ml of complete growth medium and aspirate cells by gently pipetting.
7. Transfer the cell suspension to a centrifuge tube and centrifuge at 800-1000 rpm for 3-5 minutes.
8. Discard the supernatant using a vacuum pump.
9. Add appropriate volume of complete medium. Suspend the cell pellet by gently pipetting.
10. Count the cell numbers with Vi-cell XR and adjust cells to appropriate density.
11. Add 38 μL of cell suspension to 384-well opaque-walled clear bottom plates according to the planned plate layout and place the plates in the CO_2_ incubator over night.
12. Plate layout: plate layout (each cell line was tested in one 384-well plate)

（2）Compound Preparation and Addition for the Single Agent Test:

2.1 Compound preparation

a. As shown in the table below, all the compounds were tested for six points by 5-fold dilution. The compounds were diluted into 6 concentrations in 384-well plate with the Hamilton instrument.

b. For the reference compound, 0.4mM staurosporine (200× of the final concentration) was made from the stock solution. Then 6 points with 5-fold serial dilutions were done.

c. Removed the compound plate from -30 degree freezer and thawed. Transfer 4µl of compound solutions to the corresponding wells of the intermediate plate containing 36 µl of RPMI1640 without FBS. Mix completely, transferring 2µl of the diluted compounds from intermediate plate to the cell plate wells containing 38µl of the culture medium. The total dilution is 200 fold.

d. Incubated plates for 72 hours at 5% CO_2_, 37°C.

（3）Preparation of reagents

a. Thaw the CellTiter-Glo buffer and equilibrate to room temperature prior to use.

b. Equilibrate the lyophilized CellTiter-Glo substrate to room temperature prior to use.

c. Transfer the appropriate volume of CellTiter-Glo buffer into the amber bottle containing CellTiter-Glo substrate to reconstitute the lyophilized enzyme/substrate mixture.This forms the CellTiter-Glo reagent.

d. Mix by gently vortexing, swirling or by inverting the contents to obtain a homogeneous solution. The CellTiter-Glo substrate should go into solution easily in less than one minute.

（4） Assay measurement

a. Observe the cell morphology under an inverted microscope after 72 hours treatment.

b. Equilibrate the plate and its contents to room temperature for approximately 30 minutes.

c. Add 25μL of CellTiter-Glo reagent to the assay plate by Multidrop Combi instrument.

d. Mix contents for 10 minutes on an orbital shaker to induce cell lysis.

e. Allow the plate to incubate at room temperature for 10 minutes to stabilize luminescent signal.

f. Paste the clear bottom with white back seal and record luminescence with Enspire.

g. The settings should be: Luminescence, measurement time 0.1ms.

Experimental details and characterization data for synthesized compounds

**（a）**

**Carnosic acid γ-lactone (2)**: to a solution of carnosic acid (200mg, 0.6mmol) in dry DCM (7.0ml) was added EDCI (172mg, 0.9mmol) and catalytic amount of DMAP (15mg, 0.12mmol). the mixture was stirred at room temperature for 3h. cooled in an ice-bath and after addition of water, washed with brine (3×10ml ), the product was extracted with EtOAc (3×10ml ), and dried anhydrous Na_2_SO_4_. The residue was purified by flash column chromatography on silica gel cluting with PE/EtOAc = 50 : 1 to yield **2** (180mg,95%) as colourless solid.

Compound **2**:

^1^H NMR (400 MHz, CDCl_3_): δ 6.65 (s, 1H), 4.95 (s, 1H), 3.26 (step, *J* =6.8 Hz, 1H), 2.57 (m, 2H), 2.24 (m, 1H), 2.11 (m, 1H), 2.00 (m, 1H), 1.90 – 1.83 (m, 3H), , 1.71 (m, 1H), 1.39 (m, 1H), 1.24 (d, *J* = 6.8 Hz, 3H), 1.23(d, *J* = 6.8 Hz, 3H), 1.15 (s, 3H), 1.08 (s, 3H), 0.83 (m, 1H); ^13^C NMR (100 MHz, CDCl_3_): δ 178.3, 139.7, 136.3, 135.3, 131.5, 129.9, 120.0, 56.5, 50.5, 41.7, 38.5, 32.8, 32.5, 31.7, 27.2, 24.2, 23.1, 22.3, 22.2, 18.2; ^13^C NMR (100 MHz, CDCl_3_): δ 178.3, 139.7, 136.3, 135.3, 131.5, 129.9, 120.2, 56.5, 50.0, 41.7, 38.5, 32.8, 32.5, 31.7, 27.2, 24.2, 23.1, 22.3, 22.2, 18.2; HRESIMS *m*/*z* 337.1774 [M+Na]^+^ (calcd for C_20_H_26_O_3_Na, 337.1772).

**(b)**

**Carnosic acid γ-lactone 12-** **methoxymethoxy (3)**: to a solution of 2 (240mg, 0.76mmol) in dry DCM (6.0ml) was added DIPEA (380µl, 2.3mmol) and Chloromethyl methyl ether (115µl, 1.5mmol) at 0℃, rising to room temperature for 12h, after the reaction was completed, quenched with saturated aqueous NH_4_Cl (3×5 mL) and extracted with DCM (3×5 mL). The combined organic layers were washed with brine (3×8 mL), dried over Na_2_SO_4_, filtered and concentrated under reduced pressure to give the crude product. The crude product was purified by flash chromatography (PE/EtOAc = 50:1) to afford **3** (240mg, 88%) as colourless liquid.

Compound **3**:

^1^H NMR (400 MHz, CDCl_3_): δ 6.69 (s, 1H), 5.31 (d, *J* = 6.0 Hz, 1H), 5.17 (d, *J* = 6.0 Hz 1H), 3.57 (s, 3H), 3.37 (step, *J* = 6.9 Hz, 1H), 2.60 (m, 2H), 2.22 (m, 1H), 2.10 (m, 1H), 2.00 (m, 1H), 1.86 (overlap, 3H), 1.38 (m, 2H), 1.29 (d, *J* = 6.9 Hz, 3H) , 1.25 (d, *J* = 6.9 Hz, 3H), 1.16 (s, 3H), 1.08 (s, 3H), 0.90 (m, 2H); ^13^C NMR (100 MHz, CDCl_3_): δ 178.0, 143.3, 140.8, 136.0, 134.1, 130.8, 120.2, 98.2, 57.3, 56.3, 49.2, 41.9, 38.6, 32.8, 32.7, 31.8, 26.8, 24.0, 23.7, 23.2, 22.2, 18.3; HRESIMS *m*/*z* 381.2037 [M+Na]^+^ (calcd for C_22_H_30_O_4_Na, 381.2036).

**(c)**

**A series of contain N Compounds (4)** : under argon atmosphere to a various solution of 3 (25mg,1.0eq) in dry THF (2.5ml) was added amine or hydrazine (n-butylamine, cyclohexane, piperidine, benzylamine, phenylhydrazine, hydrazine ) (5.0eq) , then the mixture was stirred for 0.5h~5h, evaporate solvent under reduced pressure to give the crude product. The crude product was purified by flash chromatography (PE/EtOAc = 30: 1) to afford **4** (90%) as pale yellow liquid.

Compound **4a:**

^1^H NMR (400 MHz, CDCl_3_): δ 7.37 (brs, 1H), 7.03 (s, 1H), 6.50 (s, 1H), 4.92 (d, *J* = 6.0 Hz, 1H), 4.90 (d, *J* = 6.0 Hz, 1H), 3.58 (s, 3H), 3.50 (dt, *J* = 14.6, 4.2Hz, 1H) 3.09 (step, *J* = 6.9 Hz, 1H), 2.85 (m, 2H), 2.23 (m, 2H), 1.86 (m, 1H), 1.65-1.50 (m, 4H), 1.32-1.27 (m, 3H), 1.19 (d, *J* = 6.9 Hz, 3H) , 1.18 (d, J = 6.9 Hz, 3H), 0.97 (s, 3H), 0.85 (s, 3H); ^13^C NMR (100 MHz, CDCl_3_): δ 177.4, 147.7, 142.0, 140.0, 134.9, 127.2, 118.1, 100.8, 57.6, 53.5, 47.5, 40.7, 34.2, 33.7, 32.6, 31.8, 26.9, 23.4, 23.2, 21.0, 20.0, 19.0; HRMS(EI) calcd for C_22_H_34_O_4_N_2_, [M]^+^: 390.2522, found 390.2519.

Compound **4b**:

^1^H NMR (400 MHz, CDCl_3_): δ 7.29 (s, 1H), 6.51 (s, 1H), 5.79 (t, *J* = 6.1 Hz, 1H), 4.96 (d, *J* = 6.0 Hz, 1H), 4.89 (d, *J* = 6.0 Hz, 1H), 3.58 (s, 3H), 3.53 (dt, *J* = 14.7, 4.6Hz, 1H), 3.18 (m, 2H), 3.10 (m, 1H), 2.30 (m, 1H), 2.14 (m, 1H), 1.88 (m, 1H), 1.70-1.50 (m, 4H), 1.30 (m, 2H) , 1.24-1.19 (m, 5H), 1.18 (d, *J* = 6.9 Hz, 3H), 1.17 (d, *J* = 6.9 Hz, 3H), 0.96 (s, 3H), 1.89 (s, 3H), 0.85 (t, *J* = 7.4 Hz, 3H) ; ^13^C NMR (100 MHz, CDCl_3_): δ 175.6, 147.8, 141.9, 139.6, 134.7, 128.1, 118.0, 100.8, 57.5, 53.5, 48.3, 40.9, 39.5, 34.5, 33.8, 32.7, 31.9, 31.9, 26.9, 23.4, 23.2, 21.2, 20.2, 20.2, 19.1, 13.8; HRESIMS *m*/*z* 454.2926 [M+Na]^+^ (calcd for C_26_H_41_NO_4_Na, 454.2928).

Compound **4c**:

^1^H NMR (400 MHz, CDCl_3_): δ 7.26 (s, 1H), 6.51 (s, 1H), 5.78 (d, *J* = 5.6 Hz, 1H), 4.97 (d, *J* = 5.4 Hz, 1H), 4.87 (d, *J* = 5.4 Hz, 1H), 3.77 (m, 1H), 3.57 (s, 3H) 3.50 (dt, *J* = 14.4, 4.3 Hz, 1H), 3.09 (step, *J* = 6.8 Hz, 1H), 2.86 (m, 2H), 2.42 (m, 2H), 2.12 (m, 1H), 1.87-1.75 (m, 3H), 1.62-1.48 (m, 5H), 1.33-1.26 (m, 4H) , 1.19 (d, *J* = 6.8 Hz, 5H), 1.19 (d, *J* = 6.8 Hz, 3H), 1.08 (m, 1H), 0.96 (s, 3H), 0.91 (s, 3H); ^13^C NMR (100 MHz, CDCl_3_): δ 174.3, 147.8, 141.8, 139.5, 134.9, 127.9, 118.0, 100.8, 57.4, 53.7, 48.2, 47.7, 41.1, 34.4, 33.9, 32.8, 32.7, 32.7, 31.9, 26.9, 25.7, 24.8, 24.7, 23.5, 23.2, 21.3, 20.2, 19.0;HRMS(EI) *m*/*z* 457.3186 [M]^+^ (calcd for C_28_H_43_NO_4_, 457.3192).

Compound **4d**:

^1^H NMR (400 MHz, CDCl_3_): δ 7.05 (s, 1H), 6.50 (s, 1H), 4.95 (d, *J* = 5.6 Hz, 1H), 4.92 (d, *J* = 5.6 Hz, 1H), 3.61 (s, 3H), 3.40 (t, *J* =4.8 Hz, 4H), 3.10 (m, 2H), 2.72 (m, 2H), 2.00 (m, 1H), 1.90 (m, 1H), 1.76 (m, 2H), 1.63 (m, 1H), 1.55-1.38 (m, 5H), 1.26 (m, 1H), 1.20 (d, *J* = 6.8 Hz, 3H), 1.17 (d, *J* = 6.8 Hz, 3H), 1.13 (m, 1H), 1.07 (s, 3H) , 1.01 (m, 2H), 0.91 (s, 3H); ^13^C NMR (100 MHz, CDCl_3_): δ 172.9, 146.1, 142.2, 139.1, 136.3, 128.7, 117.2, 101.1, 57.5, 53.5, 51.7, 47.6, 47.6, 41.4, 36.9, 34.4, 33.5, 30.1, 26.9, 25.7, 25.7, 24.9, 23.6, 23.3, 23.2, 20.0, 20.0; HRMS(EI) *m*/*z* 443.3042 [M]^+^ (calcd for C_27_H_41_NO_4_, 433.3036).

Compound **4e**:

^1^H NMR (400 MHz, CDCl_3_): δ 7.18-7.12 (m, 5H), 6.42 (s, 1H), 5.98 (t, *J* = 5.4 Hz, 1H), 4.87 (d, *J* = 6.1 Hz, 1H), 4.82 (d, *J* = 6.1 Hz, 1H), 4.43 (dd, *J* = 15.0, 6.0 Hz, 1H), 4.27 (dd, *J* = 15.0, 6.0 Hz, 1H), 3.50 (s, 3H), 3.47 (overlap, 1H), 3.02 (step, *J* = 6.9 Hz, 1H), 2.78 (m, 2H), 2.22 (m, 1H), 2.10 (m, 1H), 1.82 (m, 1H), 1.60 (m, 2H), 1.48 (m, 1H) , 1.22 (m, 3H), 1.10 (d, *J* = 6.9 Hz, 3H), 1.08 (d, *J* = 6.9 Hz, 3H), 0.98 (s, 3H), 0.83 (s, 3H); ^13^C NMR (100 MHz, CDCl_3_): δ 175.7, 147.7, 142.1, 139.7, 139.0, 134.8, 128.4, 128.4, 127.9, 127.6, 127.6, 127.0, 118.1, 100.8, 57.6, 53.5, 48.4, 43.8, 40.8, 34.5, 33.8, 32.7, 31.8, 27.0, 23.4, 23.2, 21.3, 20.2, 19.1; HRESIMS *m*/*z* 488.2772 [M+Na]^+^ (calcd for C_29_H_39_NO_4_Na, 488.2771).

Compound **4f**:

^1^H NMR (400 MHz, CDCl_3_): δ 7.60 (d, *J* = 5.4 Hz, 1H), 7.53 (s, 1H), 7.17 (t, *J* = 7.8 Hz, 2H), 6.83 (m, 3H), 6.54 (s, 1H), 4.99 (d, *J* = 5.5 Hz, 1H), 4.95 (d, *J* = 5.5 Hz, 1H), 3.65 (s, 3H), 3.62 (overlap, 1H), 3.11 (step, *J* = 6.8 Hz, 1H), 2.90 (m, 2H), 2.36 (m, 1H), 2.25 (m, 1H), 1.94 (m, 1H), 1.60 (m, 3H), 1.54 (m, 1H), 1.42-1.22 (m, 3H), 1.20 (d, *J* = 6.8 Hz, 1H) , 1.18 (m, *J* = 6.8 Hz, 3H), 0.97 (s, 3H), 0.80 (s, 3H); ^13^C NMR (100 MHz, CDCl_3_): δ 176.7, 148.9, 147.8, 142.4, 140.4, 135.3, 129.9, 129.2, 127.2, 121.0, 118.5, 114.1, 114.1, 101.1, 57.9, 53.9, 48.0, 41.4, 34.7, 34.1, 32.9, 32.0, 27.2, 23.5, 23.4, 21.6, 20.4, 19.3; HRMS(EI) *m*/*z* 466.2824 [M]^+^ (calcd for C_28_H_38_N_2_O_4_,466.2832).

**(d)**

**A series of contain N Compounds (5)** : to a solution of 4 (15mg,1.0eq) under Ar atmosphere in DCM (2.0ml) was added NaHCO_3_ (2.0eq) and m-CPBA (2.5eq) at room temperature, then the mixture was stirred for 16 h and the reaction was quenched with saturated aqueous Na_2_S_2_O_3_ (3×3mL) and washed with aqueous NaOH (3×3mL), extracted with DCM (6×3mL) dried over Na_2_SO_4_, filtered and concentrated under reduced pressure to give the crude product. The crude product was purified by flash chromatography (PE/EtOAc=20: 1) to afford **5** (45%) as yellow solid.

Compound **5b**:

^1^H NMR (400 MHz, CDCl_3_): δ 6.20 (t, *J* = 5.5 Hz, 1H), 5.28 (d, *J* = 6.0 Hz, 1H), 5.06 (d, *J* = 5.5 Hz, 1H), 3.51 (s, 3H), 3.28-3.18(m, 3H), 2.93 (d, *J* = 14.9 Hz, 1H), 2.84 (d, *J* = 5.6 Hz, 1H), 2.79 (d, *J* = 5.6 Hz, 1H), 2.53 (m, 1H), 2.38 (m, 1H), 2.04 (m, 1H), 1.86 (m, 1H), 1.63 (m, 1H), 1.50 (m, 3H) , 1.37 (m, 2H), 1.28 (m, 2H), 1.24 (d, *J* = 6.9 Hz, 3H), 1.22 (d, *J* = 6.9 Hz, 3H), 0.94 (s, 3H), 0.92 (t, *J* = 7.2 Hz, 3H), 0.85 (s, 3H) ; ^13^C NMR (100 MHz, CDCl_3_): δ 187.6, 183.4, 172.9, 153.1, 146.2, 143.6, 136.8, 98.0, 57.6, 53.4, 46.8, 41.6, 39.8, 34.2, 33.8, 32.3, 31.1, 25.0, 25.0, 20.5, 20.4, 20.3, 20.3, 20.3, 17.1, 13.7; HRMS(EI) *m*/*z* 445.2827 [M]^+^ (calcd for C_26_H_39_NO_5_, 445.2828).

Compound **5c**:

^1^H NMR (400 MHz, CDCl_3_): δ 6.09 (d, *J* = 8.0 Hz, 1H), 5.28 (d, *J* = 6.0 Hz, 1H), 5.06 (d, *J* = 6.0 Hz, 1H), 3.73 (m, 1H), 3.51(s, 3H), 3.25 (step, *J* = 6.9 Hz, 1H), 2.92 (m, 1H), 2.78 (d, *J* = 5.8 Hz, 1H), 2.58 (m, 1H), 2.38 (m, 1H), 2.02-1.80 (m, 3H), 1.80-1.45 (m, 5H), 1.40-1.35 (m, 3H), 1.25 (brs, 6H) , 1.23 (d, *J* = 6.9 Hz, 3H), 1.22 (d, *J* = 6.9 Hz, 3H), 0.93 (s, 3H), 0.87 (s, 3H) ; ^13^C NMR (100 MHz, CDCl_3_): δ 187.6, 183.8, 171.8, 153.1, 146.3, 143.5, 136.8, 98.0, 57.7, 53.5, 48.5, 46.8, 41.7, 34.2, 33.9, 32.7, 32.5, 32.3, 25.6, 25.0, 25.0, 24.9, 24.8, 20.5, 20.5, 20.4, 20.3, 17.1; HRMS(EI) *m*/*z* 471.2987 [M]^+^ (calcd for C_28_H_41_NO_5_, 471.2985).

Compound **5d**:

^1^H NMR (400 MHz, CDCl_3_): δ 5.31 (d, *J* = 5.8 Hz, 1H), 5.12 (d, *J* = 5.8 Hz, 1H), 3.54 (s, 3H), 3.27 (step, *J* = 6.8 Hz, 1H), 2.95 (dt, *J* = 14.1, 4.5 Hz, 1H), 2.74 (m, 1H), 2.46 (m, 1H), 2.20-2.00 (m, 2H), 1.85 (m, 1H), 1.70-1.45 (m, 9H), 1.35 (m, 1H), 1.26 (d, *J* = 6.8 Hz, 3H), 1.23 (d, *J* = 6.8 Hz, 3H), 1.16 (m, 2H) , 0.92 (s, 3H), 0.82 (s, 3H) ; ^13^C NMR (100 MHz, CDCl_3_): δ 187.8, 183.5, 173.0, 153.2, 146.7, 143.9, 136.7, 98.1, 57.7, 54.8, 48.7, 47.6, 47.6, 40.5, 37.5, 33.6, 32.3, 25.9, 25.9, 25.0, 24.8, 24.6, 20.6, 20.5, 20.4, 19.9, 17.3; HRMS(EI) *m*/*z* 480.2721 [M+Na]^+^ (calcd for C_27_H_39_NO_5_Na, 480.2720).

**(e)**

**Royleanone derivatives (6):** to a series of above solution of 5 (10mg, 1.0eq) in MeOH (1ml) was added hydrochloric acid (1.2eq). After being stirred overnight the reaction was quenched with saturated aqueous NaHCO_3_ (3×3 mL) and extracted with EA (3×3 mL). The combined organic layers were washed with brine (3×3 mL), dried over Na_2_SO_4_, filtered and concentrated under reduced pressure to give the crude product and purification by flash chromatography (PE/EtOAc = 20:1) to afford **6** (60%) as yellow solid.

**Compound 6g:**

^1^H NMR (400 MHz, CDCl_3_): δ 7.10 (s, 1H), 6.21 (br d, 1H), 5,52 (br d, 1H), 3.15 (step, *J* =6.8 Hz, 1H), 3.10 (m, 1H), 2.56-2.38 (m, 2H), 2.08 (m, 1H), 1.90 (m, 1H), 1.68 (m, 1H), 1.54 (m, 3H), 1.26 (m, 5H), 1.21 (d, *J* = 6.8 Hz, 3H), 1.19 (d, *J* = 6.8 Hz, 3H), 0.96 (s, 3H), 0.92 (s, 3H); ^13^C NMR (100 MHz, CDCl_3_): δ 186.9, 183.1, 176.0, 150.4, 149.7, 140.3, 124.9, 53.6, 46.8, 41.5, 34.7, 33.9, 32.3, 26.1, 20.4, 20.2, 19.9, 19.8, 17.2, 14.2; HRMS(EI) calcd for C_20_H_27_O_4_N [M]^+^: 345.1949, found 345.1940.

Compound **6b**:

^1^H NMR (400 MHz, CDCl_3_): δ 7.10 (s, 1H), 6.25 (t, *J* = 5.0 Hz, 1H), 3.22 (m, 2H), 3.15 (step, *J* =6.9 Hz, 1H), 3.08 (m, 1H), 2.87 (m, 1H), 2.43 (m, 1H), 1.97 (m, 1H), 1.86 (m, 1H), 1.35 (m, 2H), 1.26 (m, 3H), 1.21 (d, *J* = 6.8 Hz, 3H), 1.19 (d, *J* = 6.8 Hz, 3H), 0.95 (s, 3H), 0.93 (t, *J* = 7.3 Hz, 3H), 0.85 (s, 3H); ^13^C NMR (100 MHz, CDCl_3_): δ 186.9, 183.2, 172.6, 150.3, 149.7, 140.4, 124.7, 53.8, 46.8, 41.6, 39.8, 34.2, 33.8, 32.3, 31.1, 26.1, 24.2, 20.5, 20.3, 20.3, 19.9, 19.8, 17.2, 13.7; HRMS(EI) calcd for C_24_H_35_O_4_N [M]^+^: 401.2559, found 401.2566.

Compound **6e**:

^1^H NMR (400 MHz, CDCl_3_): δ 7.07 (s, 1H), 6.13 (d, *J* = 7.8 Hz, 1H), 3.73 (m, 1H), 3.15 (step, *J* =6.8 Hz, 1H), 3.03 (m, 1H), 2.86 (m, 1H), 2.65 (m, 1H), 2.45 (m, 1H), 1.92-1.78 (m, 6H), 1.69-1.49 (m, 9H), 1.19 (d, *J* = 6.8 Hz, 3H), 1.18 (d, *J* = 6.8 Hz, 3H), 0.94 (s, 3H), 0.87 (s, 3H); ^13^C NMR (100 MHz, CDCl_3_): δ 187.0, 183.1, 171.5, 150.3, 149.8, 140.4, 124.7, 53.8, 48.4, 46.8, 41.7, 34.2, 33.9, 32.7, 32.6, 26.1, 24.6, 24.8, 24.8, 24.2, 20.3, 20.3 19.9, 19.8, 17.2; HRMS(EI) calcd for C_26_H_37_O_4_N [M]^+^: 427.2725, found 427.2723.

Compound **6c**:

^1^H NMR (400 MHz, CDCl_3_): δ 7.09 (s, 1H), 3.15 (step, *J* =6.8 Hz, 1H), 2.71 (m, 1H), 2.55 (m, 1H), 2.17 (m, 1H), 2.04 (m, 1H), 1.85 (m, 1H), 1.66-1.51 (m, 8H), 1.31 (m, 4H), 1.21 (d, *J* = 6.8 Hz, 3H), 1.19 (d, *J* = 6.8 Hz, 3H), 0.93 (s, 3H), 0.84 (s, 3H); ^13^C NMR (100 MHz, CDCl_3_): δ 187.2, 183.0, 172.7, 150.3, 147.9, 142.0, 124.7, 55.3, 48.9, 47.5, 47.5, 40.6, 37.3, 33.8, 32.4, 26.0, 26.0, 25.4, 24.6, 24.2, 20.7, 19.9, 19.8, 19.8, 17.5; HRMS(EI) calcd for C_25_H_35_O_4_N [M]^+^: 413.2574, found 413.2566.

Compound **6d**:

^1^H NMR (400 MHz, CDCl_3_): δ 7.33 (m, 5H), 7.10 (s, 1H), 6.50 (t, *J* = 4.7 Hz, 1H), 4.41 (d, *J* = 5.2 Hz, 2H), 3.15 (step, *J* = 6.9 Hz, 1H), 3.09 (m, 1H), 2.87 (m, 1H), 2.60 (m, 1H), 2.46 (m, 1H), 1.90 (m, 2H), 1.63-1.49 (m, 2H), 1.27 (m, 3H), 1.21 (d, *J* = 6.8 Hz, 3H), 1.19 (d, *J* = 6.8 Hz, 3H), 0.95 (s, 3H), 0.84 (s, 3H); ^13^C NMR (100 MHz, CDCl_3_): δ 186.9, 183.2, 172.7, 150.4, 149.8, 140.3, 137.9, 128.7, 128.7, 128.1, 128.1, 127.6, 124.8, 53.8, 46.9, 44.4, 41.5, 34.1, 33.8, 32.3, 26.1, 24.2, 20.5, 20.3, 19.9, 19.8, 17.2; HRMS(EI) calcd for C_27_H_33_O_4_N [M]^+^: 435.2410, found 435.2413.

Compound **12:**

^1^H NMR (400 MHz, Acetone-*d_6_*): δ 11.8 (brd, 1H), 8.87 (s, 1H), 3.18 (m, 1H), 3.06 (m, 1H), 2.73 (dd, *J* = 20.0, 5.5 Hz, 1H), 2.38 (m, 2H), 1.90 (m, 1H), 1.52 (m, 2H), 1.45 (d, *J* = 12.3 Hz, 1H), 1.28 (m, 1H), 1.21 (d, *J* = 6.8 Hz, 3H), 1.19 (d, *J* = 6.8 Hz, 3H), 1.11(m, 1H), 0.97 (s, 3H), 0.88 (s, 3H); ^13^C NMR (100 MHz, Acetone-*d_6_*): δ 188.0, 183.2, 175.5, 152.7, 147.3, 143.4, 125.0, 53.1, 47.2, 41.7, 34.9, 34.3, 32.8, 26.8, 24.9, 20.5, 20.3, 20.2, 19.9, 17.7.

**(f)**

**Compounds (7):** to a solution of 2 (25mg, 0.08mmol) in THF (1.5ml) was added piperidine (38µl, 0.4mmol). After being stirred 30min the reaction was evaporate solvent under reduced pressure to give the crude product. The crude product was purified by flash chromatography (PE/EtOAc = 10: 1) to afford **7** (17mg, 65%) as pale yellow solid.

Compound **7**:

^1^H NMR (400 MHz, CDCl_3_): δ 6.51 (s, 1H), 6.16 (s, 1H), 5.56 (s, 1H), 3.40 (m, 2H), 3.24-3.09 (m, 4H), 2.87 (step, J = 6.8 Hz, 1H), 2.81 (m, 1H), 2.09 (m, 1H), 1.86 (m, 2H), 1.72-1.54 (m, 4H), 1.42 (m, 3H), 1.26 (m, 2H), 1.21 (d, J = 6.8 Hz, 3H), 1.19 (d, J = 6.8 Hz, 3H), 1.12 (m, 2H), 0.95 (s, 3H), 0.89 (s, 3H); ^13^C NMR (100 MHz, CDCl_3_): δ 176.3, 141.7, 140.1, 132.9, 127.7, 125.2, 118.2, 53.9, 49.9, 48.4, 48.4, 40.8, 38.1, 34.2, 32.5, 30.5, 27.1, 25.6, 25.6, 24.5, 22.6, 22.4, 21.9, 19.9, 19.1; HRMS(EI) calcd for C_25_H_37_O_3_N [M]^+^: 399.2773, found 399.2770.

**(g)**

**Compounds (8):** to a solution of 3 (170mg, 0.48mmol) in MeOH (8.0ml) was added NaOCH_3_ (51mg,0.95mmol). After being stirred overnight the reaction was quenched with diluted hydrochloric acid aqueous (3×10 mL) and extracted with EA (3×10 mL). The combined organic layers were washed with brine, dried over Na_2_SO_4_, filtered and concentrated under reduced pressure to give the crude product and purification by flash chromatography (PE/EtOAc = 50:1) to afford **8** (150mg, 80%) as colourless solid.

**Compound 8:**

^1^H NMR (400 MHz, CDCl_3_): δ 7.12 (s, 1H), 6.50 (s, 1H), 4.95 (dd, *J* =14.4, 6.9 Hz ,2H), 3.61 (s, 3H), 3.57 (s, 3H), 3.11 (step, *J* =6.9 Hz, 1H), 2.83 (m, 2H), 2.23 (m, 2H), 1.80 (m, 1H), 1.55 (m, 3H), 1.27 (m, 2H), 1.20 (d, J = 6.9 Hz, 3H), 1.17 (d, J = 6.9 Hz, 3H), 0.97 (s, 3H), 0.78 (s, 3H) ; ^13^C NMR (100 MHz, CDCl_3_): δ 176.4, 147.9, 141.7, 139.4, 134.9, 126.9, 117.7, 100.8, 57.6, 54.2, 51.5, 47.9, 41.3, 34.2, 33.9, 32.7, 32.1, 26.9, 23.5, 23.1, 20.0, 20.0, 18.5.

**Compounds (9):** to a solution of 8 (34 mg,1.0 eq) under Ar atmosphere in DCM (0.8 mL) was added NaHCO_3_ (14.6 mg, 2.0 eq) and m-CPBA (30.0 mg,2.5 eq) at room temperature, then the mixture was stirred for 16 h and the reaction was quenched with saturated aqueous Na_2_S_2_O_3_ (3×3mL) and washed with aqueous NaOH (3×3mL), extracted with DCM (6×3mL), dried over Na_2_SO_4_, filtered and concentrated under reduced pressure to give the crude product. The crude product was purified by flash chromatography (PE/EtOAc=20: 1) to afford **9** (15 mg, 45%) as yellow solid.

Compound **9:**

^1^H NMR (400 MHz, CDCl_3_): 4.94 (dd, *J* =14.4, 6.9 Hz ,2H), 3.60 (s, 3H), 3.57 (s, 3H), 3.13 (step, *J* =6.8 Hz, 1H), 2.83 (m, 2H), 2.23 (m, 2H), 1.80 (m, 1H), 1.55 (m, 3H), 1.27 (m, 2H), 1.21 (d, J = 6.8 Hz, 3H), 1.18 (d, J = 6.8 Hz, 3H), 0.96 (s, 3H), 0.79 (s, 3H) ; ^13^C NMR (100 MHz, CDCl_3_): δ 187.6, 183.5, 174.6, 153.3, 145.0, 144.5, 136.4, 97.9, 57.6, 52.8, 52.1, 46.5, 40.9, 34.1, 33.4, 32.3, 25.2, 25.0, 20.5, 20.4, 19.7, 19.2, 16.7; HRMS(EI) *m*/*z* 404.2200 [M]^+^ (calcd for C_23_H_32_O_6_, 404.2219).

**(h)**

**Compounds (10):** to a solution of 3 (20mg, 0.05mmol) in MeOH-H_2_O(0.8ml-0.4ml) was added LiOH (2.5mg, 0.1mmol). After being stirred 2h the reaction was quenched with diluted hydrochloric acid aqueous (3×3 mL) and extracted with EA (3×3 mL). The combined organic layers were washed with brine, dried over Na_2_SO_4_, filtered and concentrated under reduced pressure to give the crude product and purification by flash chromatography (PE/EtOAc = 8:1) to afford **10** (11mg, 60%) as colourless liquid.

Compound **10**:

^1^H NMR (400 MHz, CDCl_3_): δ 6.49 (s, 1H), 4.90 (m, 2H), 3.53 (s, 3H), 3.11 (m, 1H), 2.80 (m, 2H), 2.30 (m, 2H), 1.81 (m, 1H), 1.51 (m, 2H), 1.26 (m, 2H), 1.19 (d, *J* = 6.9 Hz, 3H), 1.17 (d, *J* = 6.9 Hz, 3H), 0.95 (s, 3H), 0.86 (s, 3H). ^13^C NMR (100 MHz, CDCl_3_): δ 171.3, 148.1, 141.7, 139.5, 135.0, 126.9, 117.9, 101.4, 60.5, 57.3, 54.2, 41.7, 34.1, 32.9, 32.2, 26.7, 23.5, 23.2, 21.1, 20.2, 18.6, 14.2.

Compound **11** was obtained following the condition d as described in the transformation of **4** to **5**

Compound **11**:

^1^H NMR (400 MHz, CDCl_3_): δ 5.35 (d, *J* = 6.2 Hz, 1H), 5.09 (d, *J* = 6.2 Hz, 1H), 3.50 (s, 3H), 3.27 (step, *J* = 6.9 Hz, 1H), 2.83-2.73 (m, 2H), 2.31 (m, 2H), 1.99 (m, 1H), 1.87 (m, 1H), 1.54 (m, 1H), 1.38 (m, 1H), 1.24 (d, *J* = 6.9 Hz, 3H), 1.22 (d, *J* = 6.9 Hz, 3H), 1.18 (m, 1H), 1.05 (m, 1H) , 0.94 (s, 3H), 0.84 (s, 3H); ^13^C NMR (100 MHz, CDCl_3_): δ 187.6, 183.3, 179.9, 153.3, 144.8, 144.5, 136.4, 97.9, 57.6, 52.8, 46.3, 41.0, 34.1, 33.7, 32.5, 25.2, 25.0, 20.5, 20.4, 19.6, 19.2, 16.7; HRMS(EI) *m*/*z* 390.2050 [M]^+^ (calcd for C_22_H_30_O_6_, 390.2044).

**(i)**

**Deoxyneocryptotanshinone:** to a solution of miltirone (25mg, 0.08mmol) in MeOH (2.0ml) was added p-TsOH (16.5mg, 0.09mmol). The mixture was reflux at 65℃ and stirred for 7h ,Then the reaction was quenched with saturated aqueous NaHCO_3_ (3×3 mL) and extracted with EA (3×3 mL). The combined organic layers were washed with brine (3×3 mL), dried over Na_2_SO_4_, filtered and concentrated under reduced pressure to give the crude product and purification by flash chromatography (PE/EtOAc = 50:1) to afford Deoxyneocryptotanshinone (17mg, 70%) as reddish brown solid.

**Deoxyneocryptotanshinone:**^1^H NMR (400 MHz, CDCl_3_): δ 7.99 (d, *J* = 7.0 Hz, 1H), 7.73 (s, 1H), 7.72 (d, *J* = 7.0 Hz, 1H), 3.34 (step, *J* = 6.9 Hz, 1H), 3.23 (m, 2H), 1.81 (m, 2H), 1.65 (m, 2H), 1.29 (s, 6H), 1.26 (d, 3H), 1.26 (d, 3H); ^13^C NMR (100 MHz, CDCl_3_): δ 184.7, 183.4, 153.2, 152.5, 140.7, 133.3, 132.7, 126.4, 126.1, 125.0, 37.7, 34.8, 31.8, 31.8, 29.9, 24.4, 19.9, 19.9, 19.1.

**(j)**

**Arucadiol and miltionone:** to a solution of 1-oxomiltirone (20mg, 0.067mmol) in MeOH(1.5ml) was added *p*-TsOH (14mg, 0.08mmol). After being stirred 7h the reaction was quenched with saturated aqueous NaHCO_3_ and extracted with EA (3×3 mL). The combined organic layers were washed with brine, dried over Na_2_SO_4_, filtered and concentrated under reduced pressure to give the crude product and purification by flash chromatography (PE/EtOAc=50:1) to afford arucadiol (9.0mg,45%) as yellow solid and miltionone I (9.5mg, 45%) as reddish brown solid.

**Arucadiol:**

^1^H NMR (400 MHz, CDCl_3_): δ 10.64 (s, 1H), 7.96 (d, *J* = 8.5 Hz, 1H), 7.31 (d, *J* = 8.5 Hz, 1H), 3.43 (step, *J* = 6.9 Hz, 3H), 2.92 (t, *J* = 6.8 Hz, 1H), 2.07 (t, *J* =6.8 Hz, 1H), 1.44 (s, 6H), 1.33 (d, *J* = 6.9 Hz, 3H), 1.32 (d, *J* = 6.9 Hz, 3H); ^13^C NMR (100 MHz, CDCl_3_): δ 203.4, 157.3, 144.0, 137.1, 136.7, 135.6, 126.8, 126.8, 124.3, 119.3, 119.2, 117.6, 35.3, 35.0, 34.5, 28.7, 28.7, 26.7, 21.2, 21.2.

**Miltionone:**

^1^H NMR (400 MHz, CDCl_3_): δ 8.19 (s, *J* = 8.0 Hz, 1H), 7.70 (d, *J* = 8.0 Hz, 1H), 7.38 (s, 1H), 3.37 (step, *J* = 6.9 Hz, 1H), 2.95 (t, *J* = 7.3 Hz, 2H), 2.11 (t, *J* =6.8 Hz, 2H), 1.35 (s, 6H), 1.30 (d, *J* = 6.9 Hz, 3H), 1.28 (d, *J* = 6.9 Hz, 3H); ^13^C NMR (100 MHz, CDCl_3_): δ 199.7, 183.6, 181.0, 156.9, 153.4, 135.0, 133.3, 130.5, 129.3, 128.7, 127.3, 36.6, 36.5, 35.4, 28.7, 28.7, 24.6, 19.8, 19.8.

**Compound 2: ^1^H NMR**

**
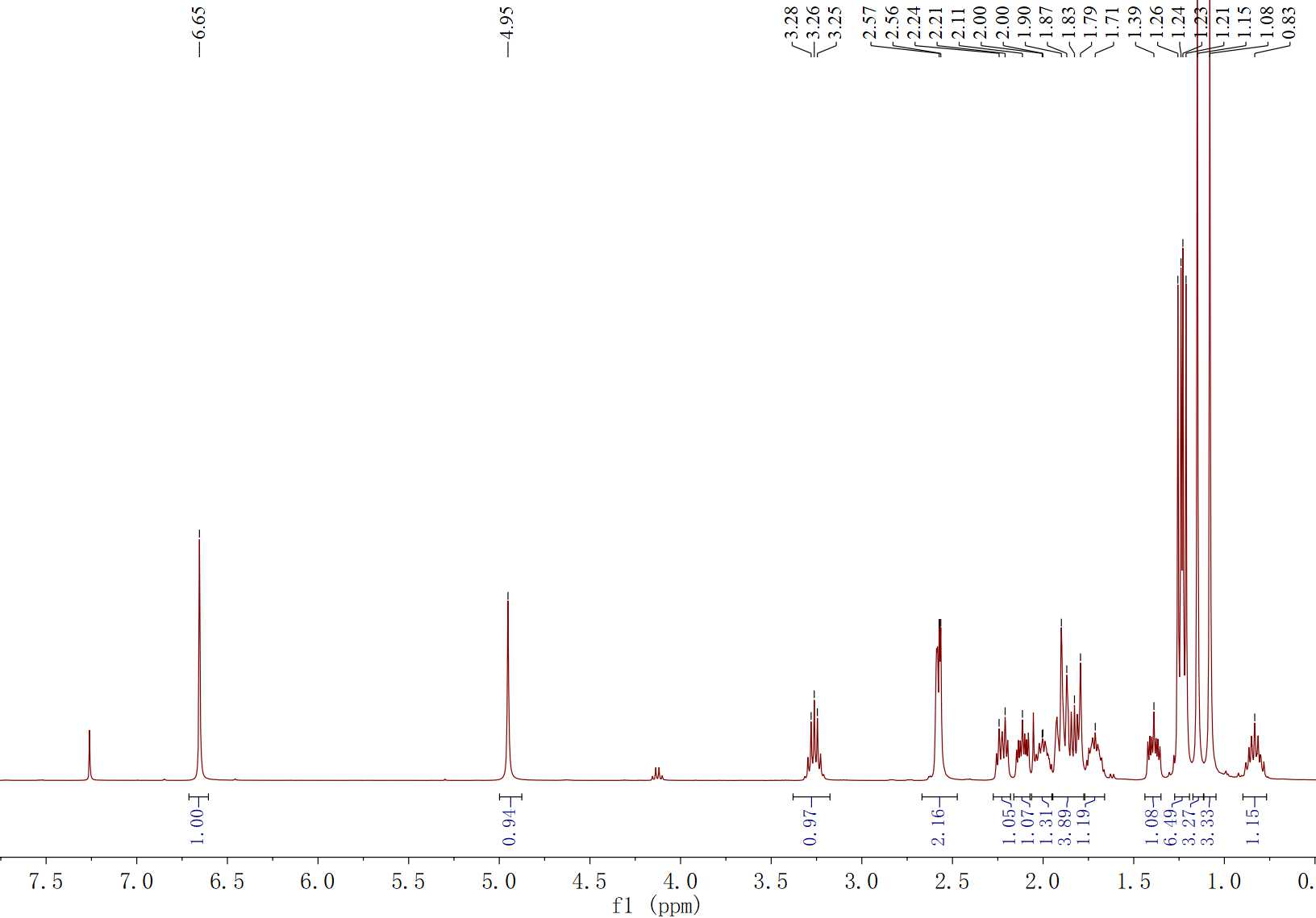
**

**Compound 2: ^1^H NMR**

**
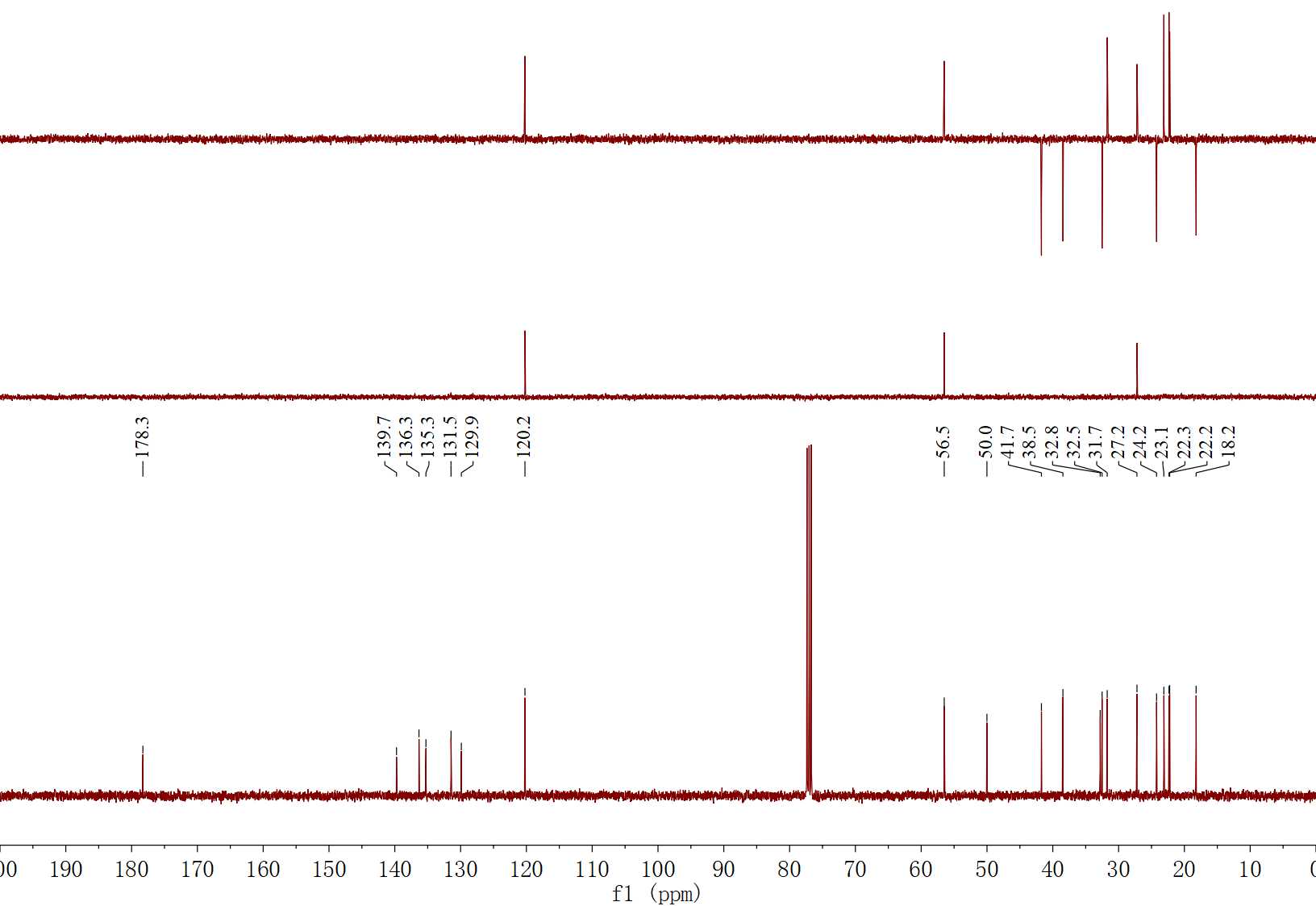
**

**Compound 3: ^1^H NMR**


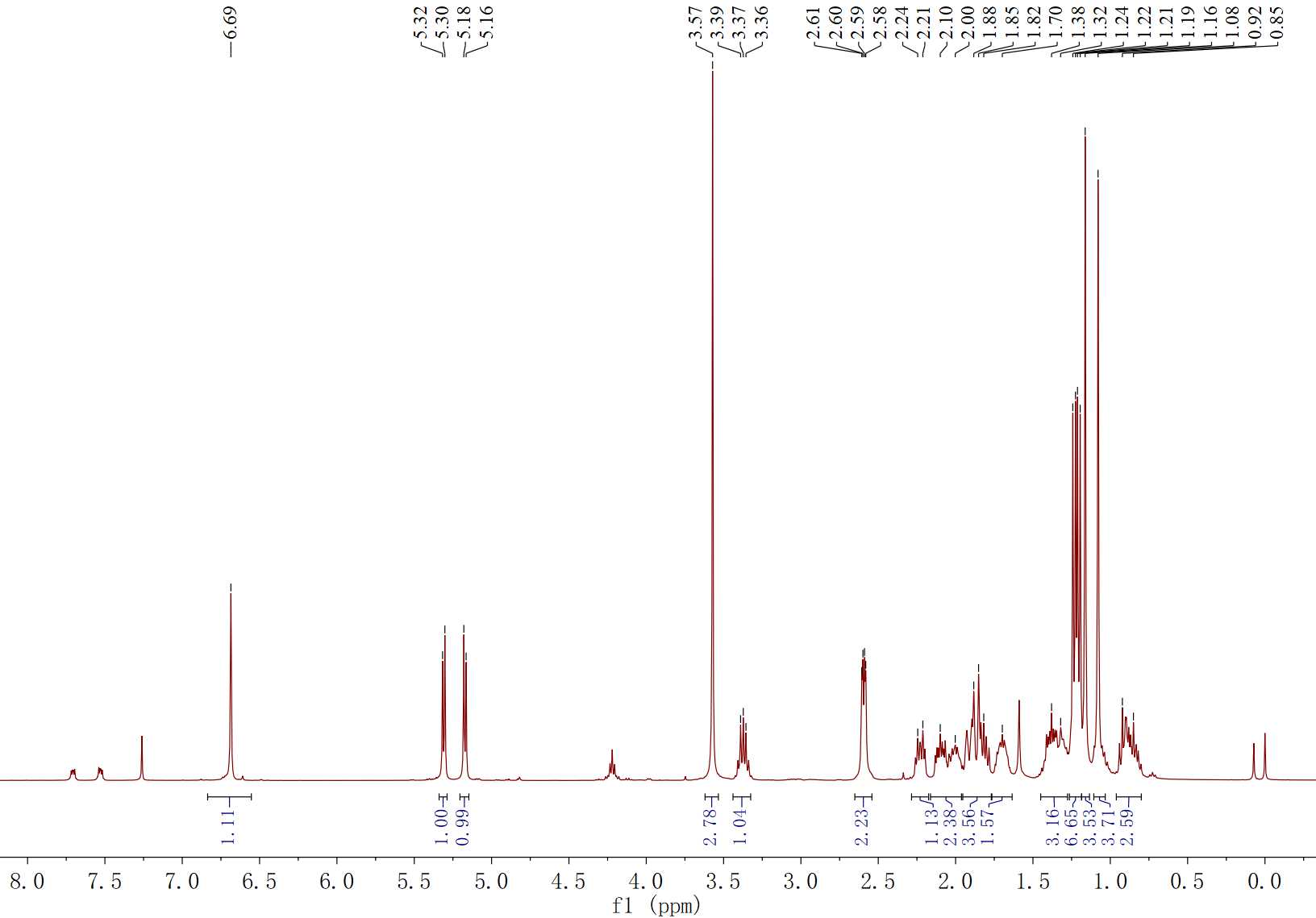


**Compound 3: ^13^C NMR**


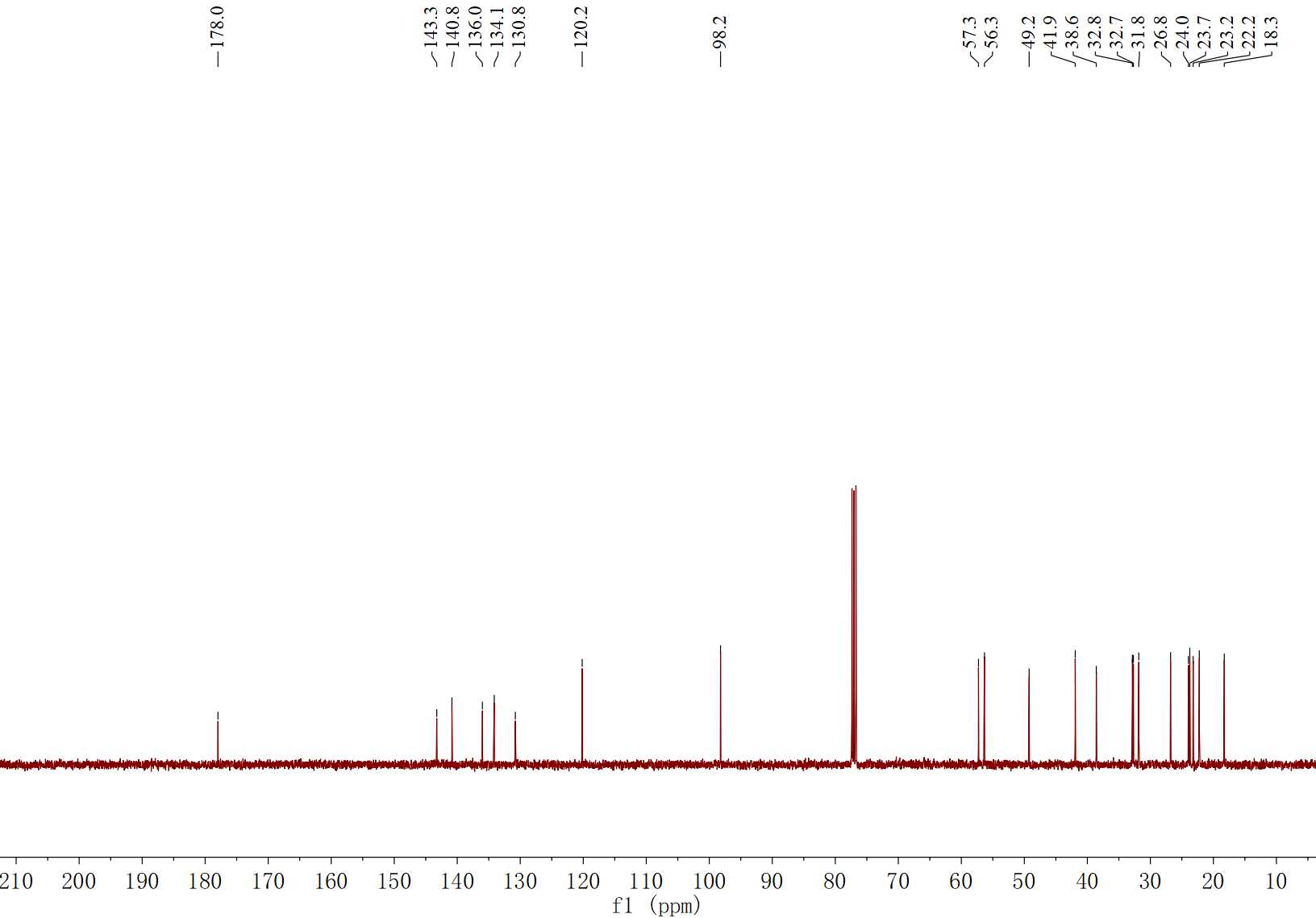


**Compound 4a: ^1^H NMR**


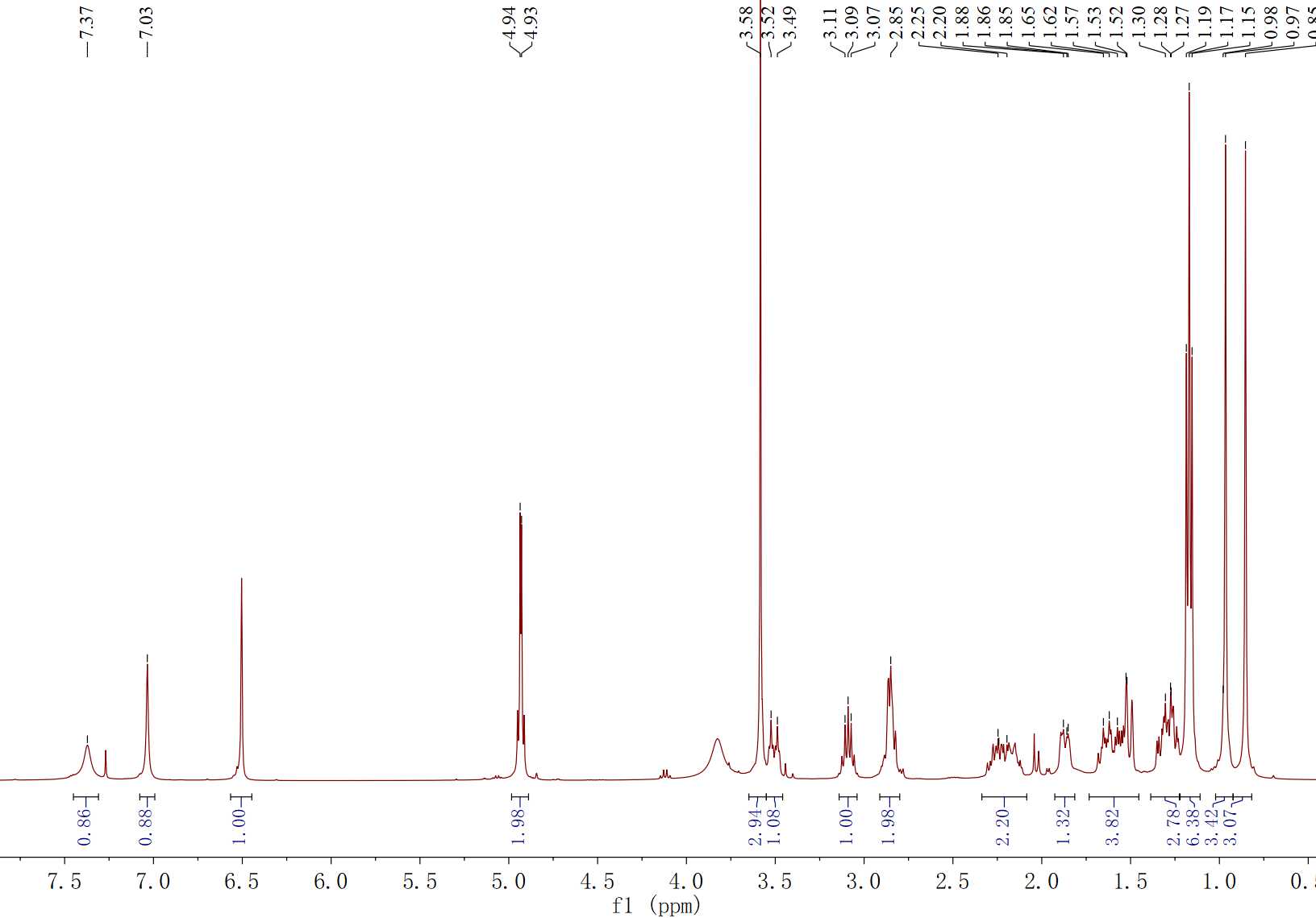


**Compound 4a: ^13^C NMR**

**
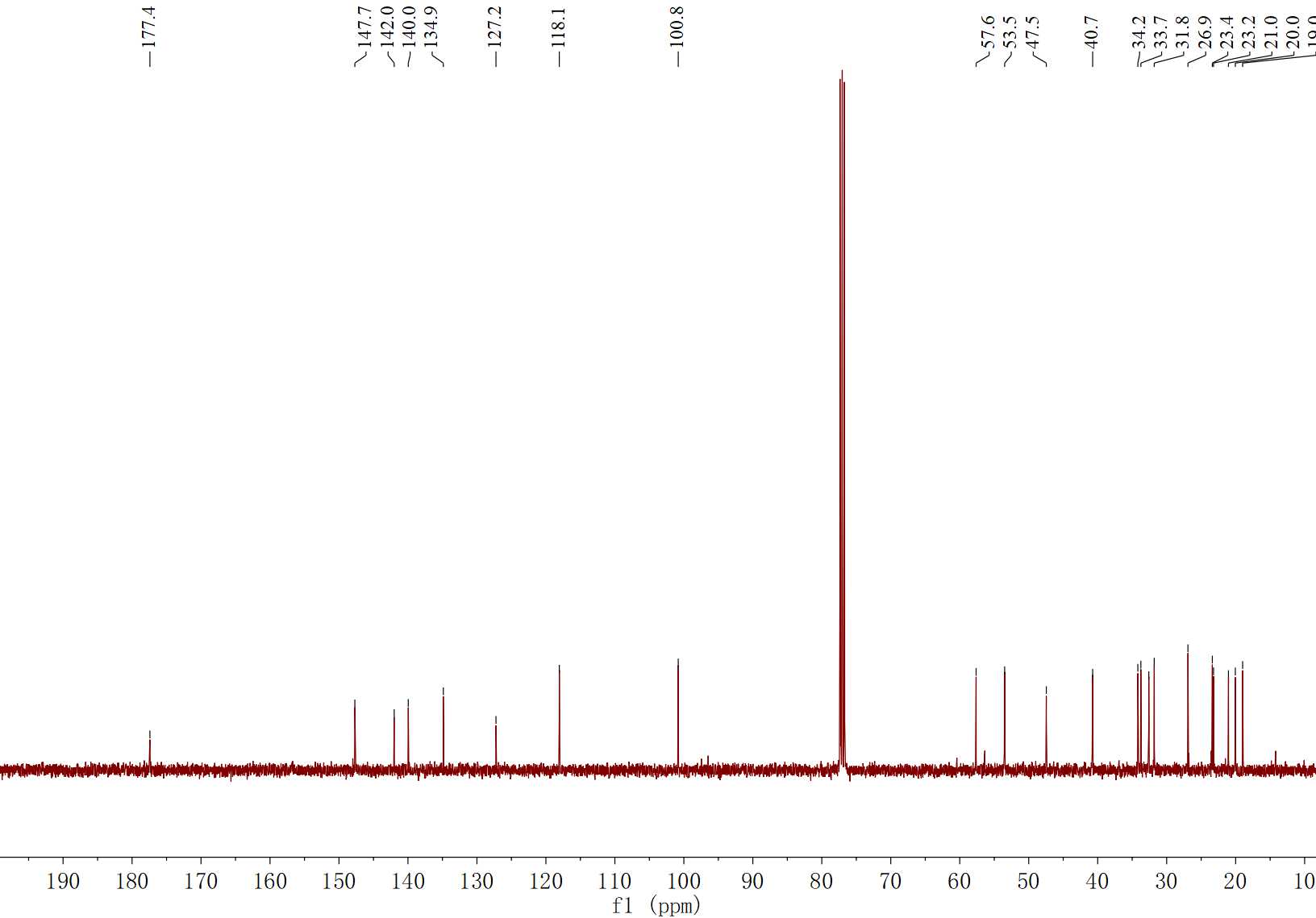
**

**Compound 4b: ^1^H NMR**


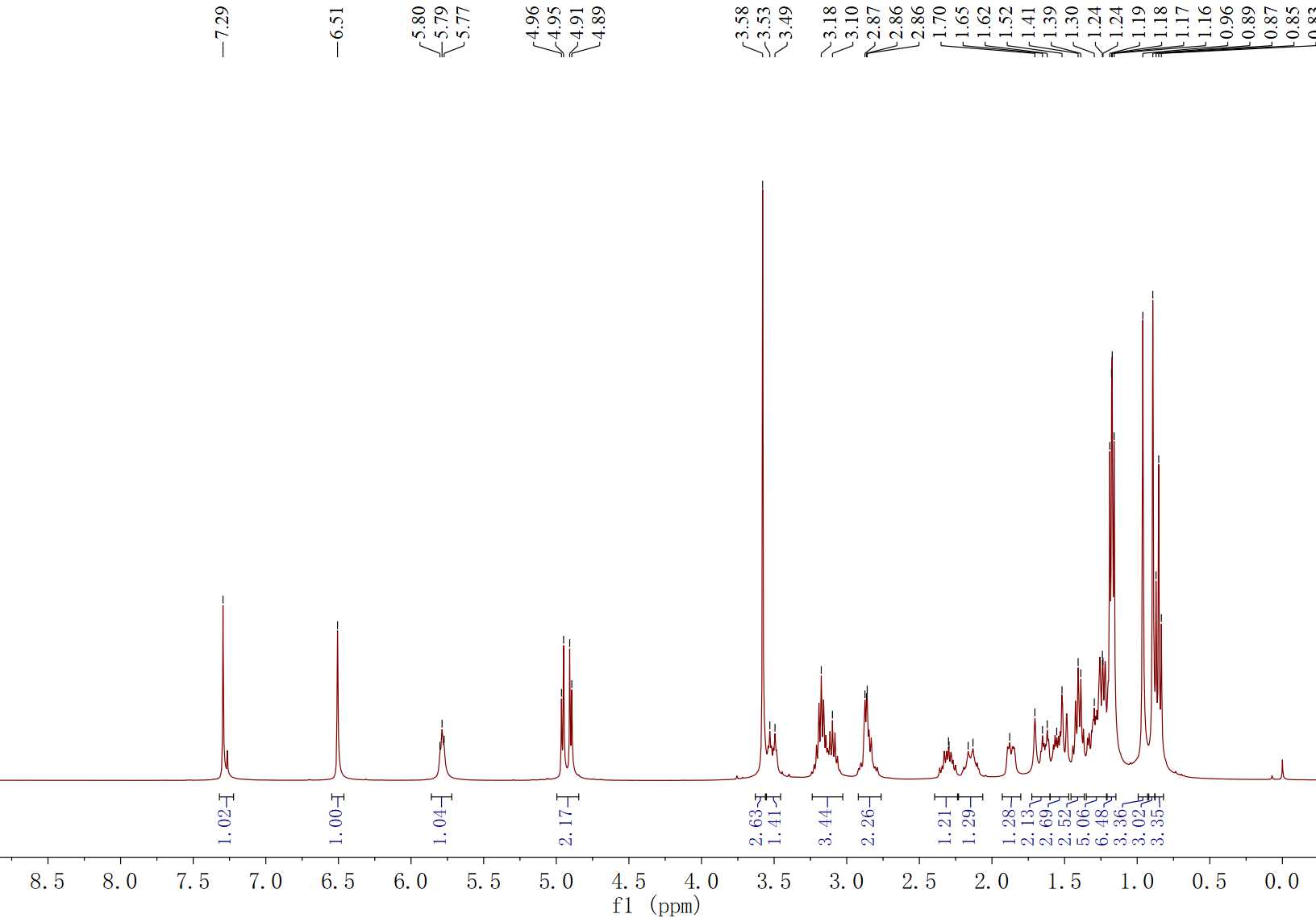


**Compound 4b: ^13^C NMR**


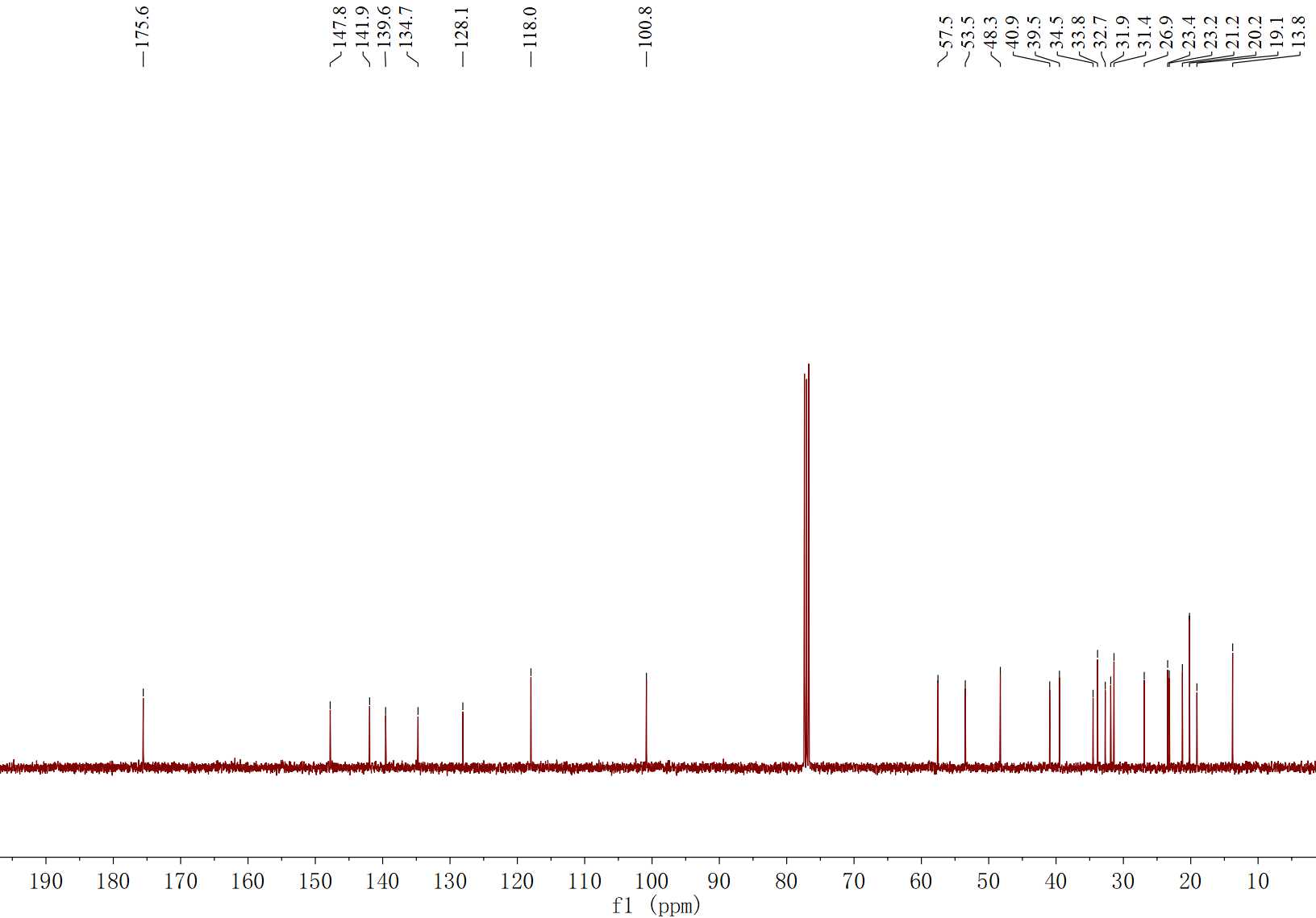


**Compound 4c: ^1^H NMR**


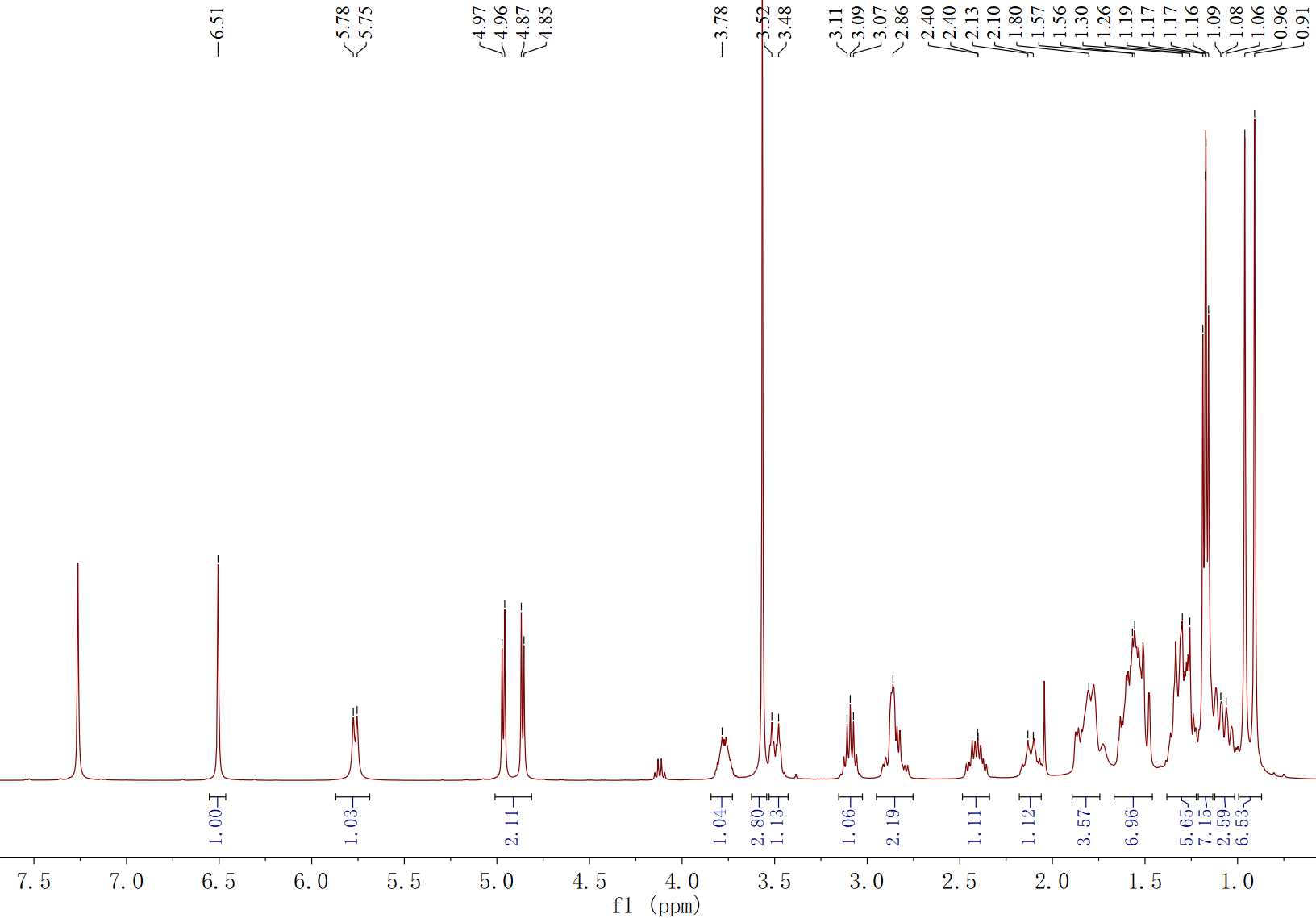


**Compound 4c: ^13^C NMR**


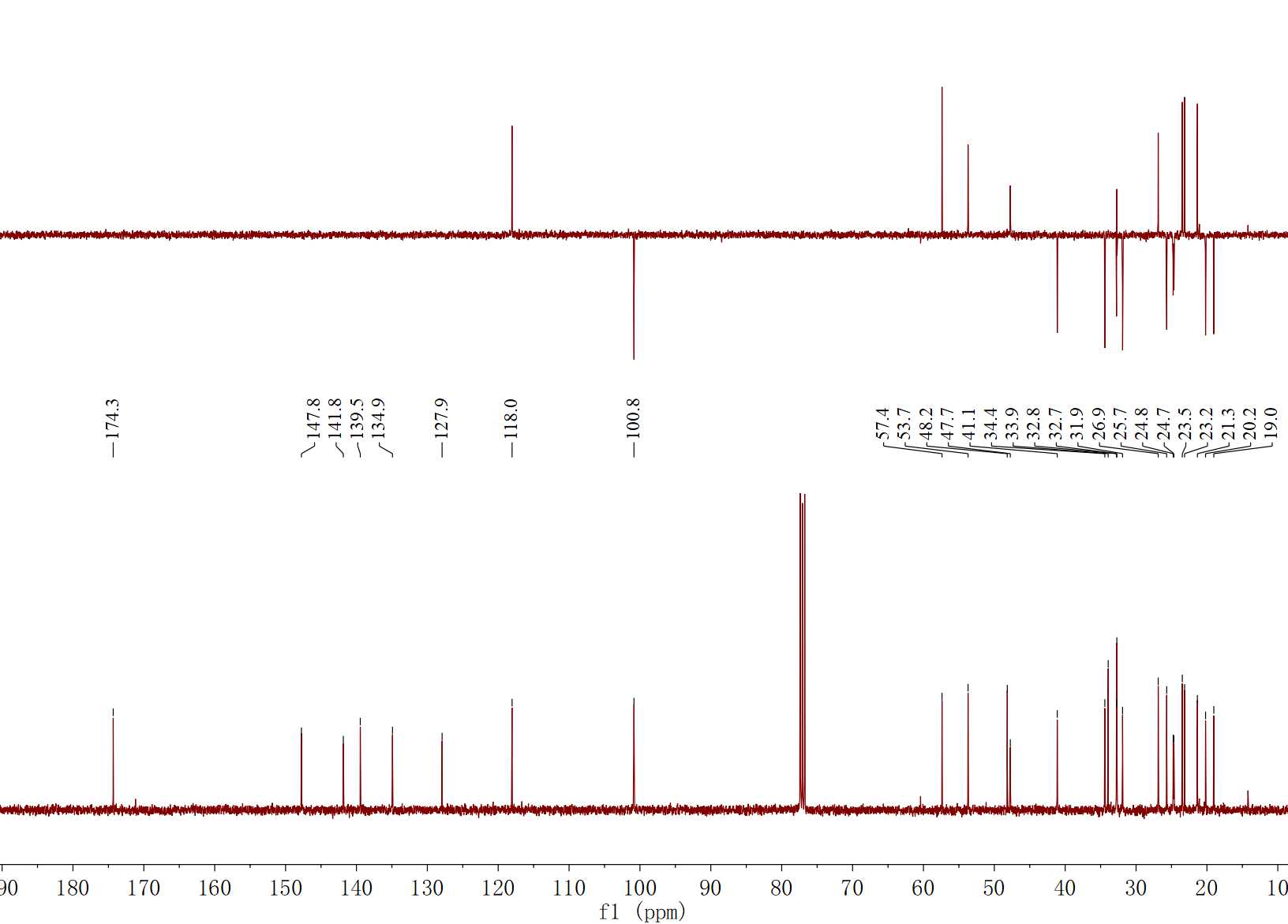


**Compound 4d: ^1^H NMR**


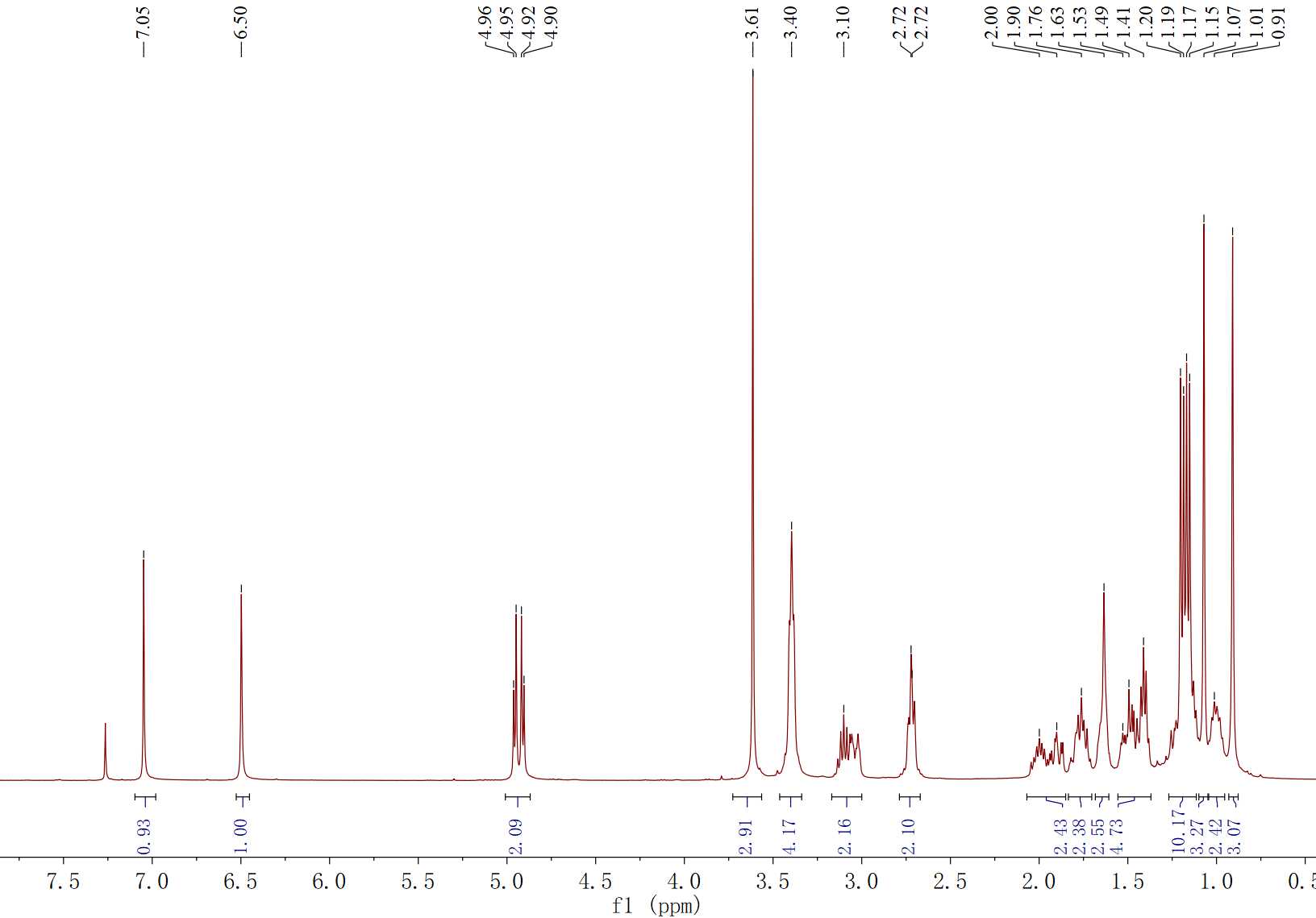


**Compound 4d: ^13^C NMR**


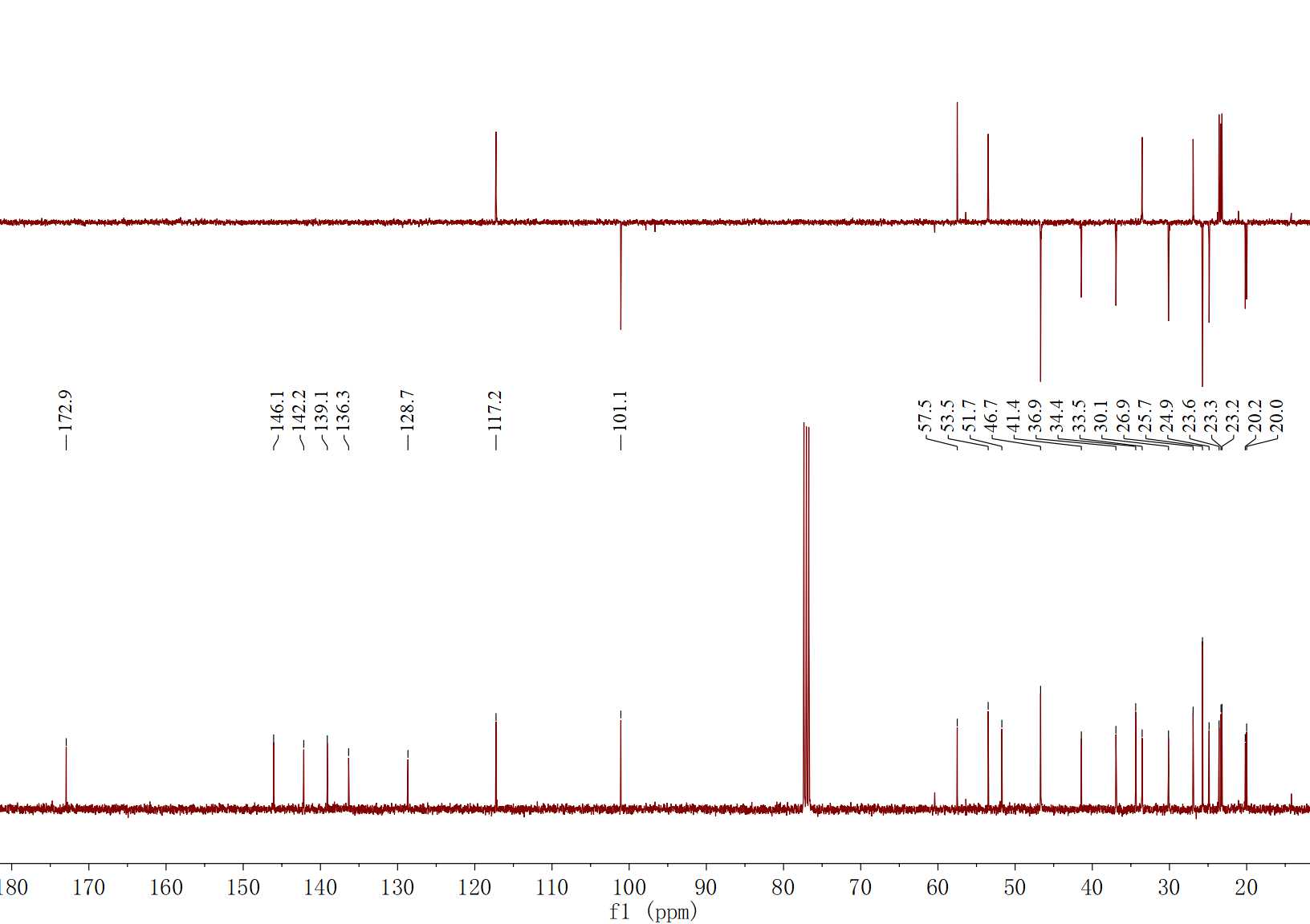


**Compound 4e: ^1^H NMR**


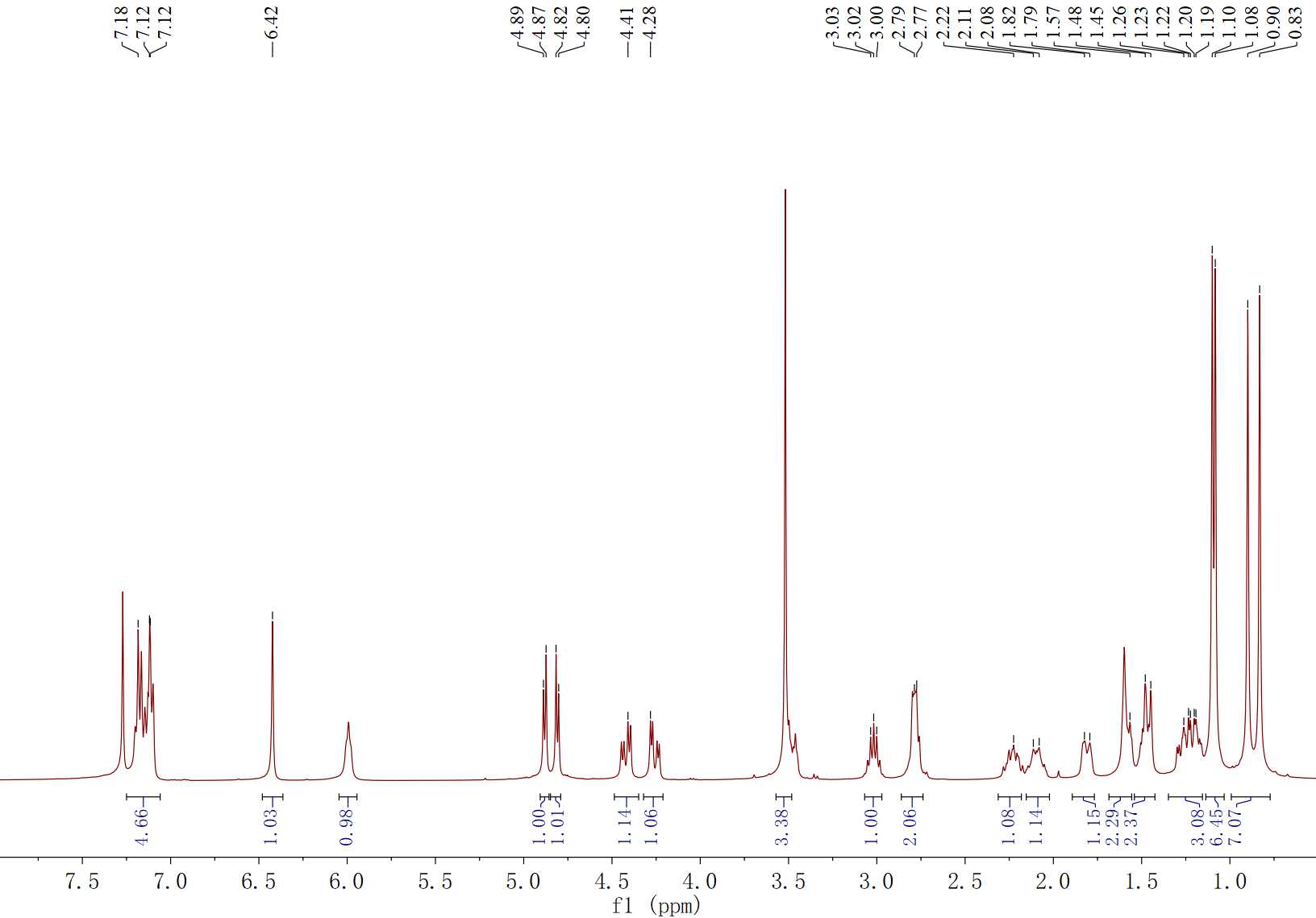


**Compound 4e: ^13^C NMR**


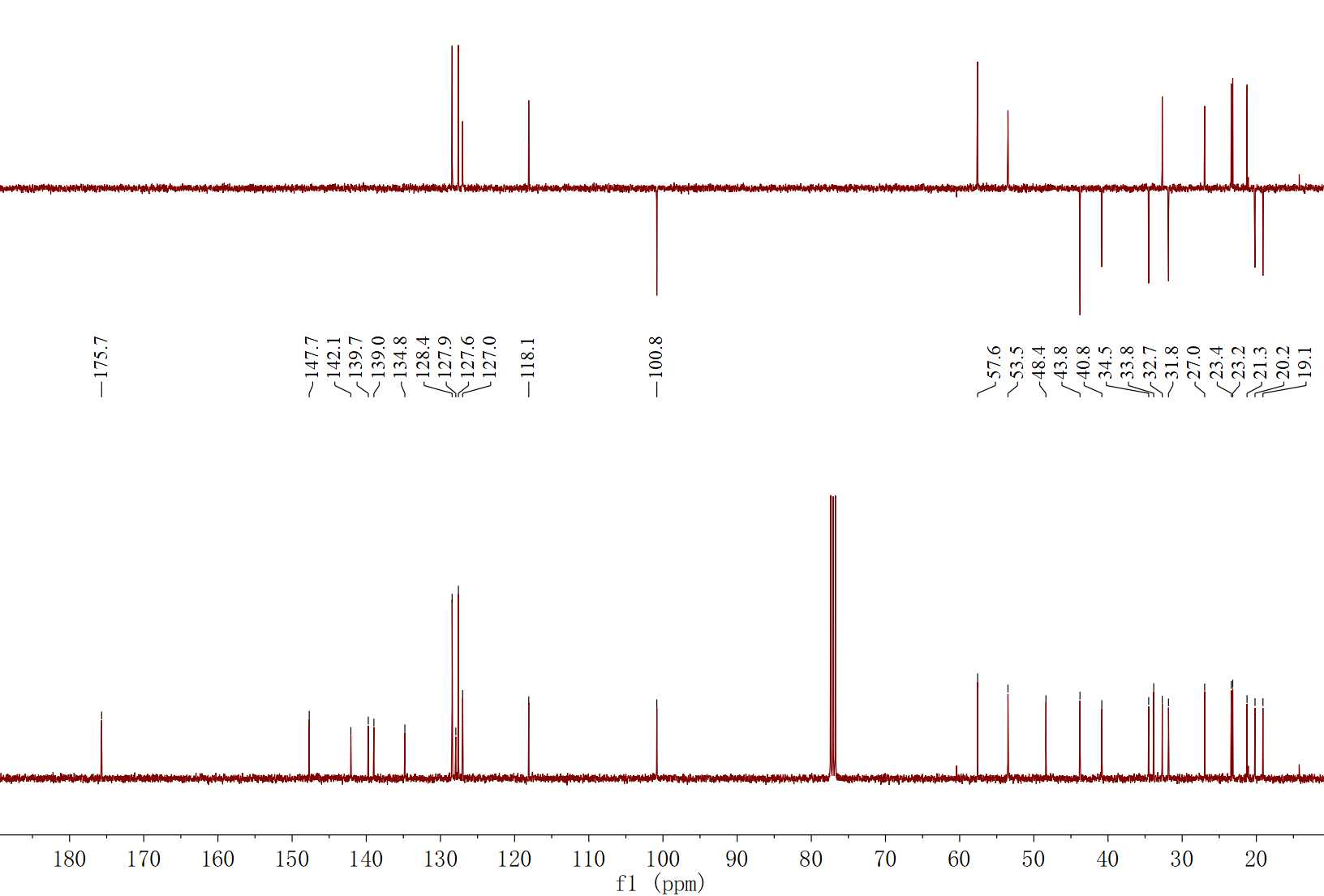


**Compound 4f: ^1^H NMR**


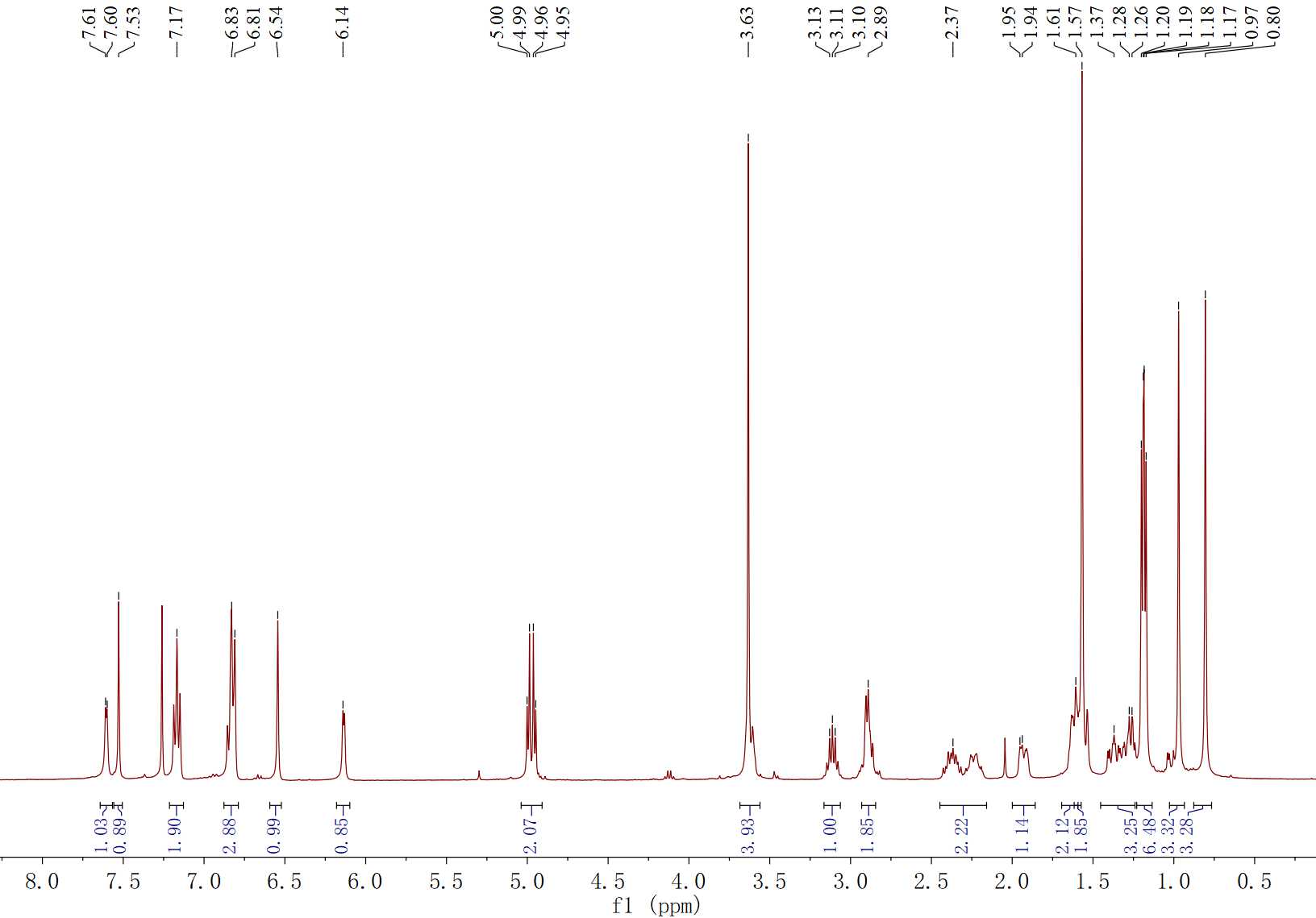


**Compound 4f: ^13^C NMR**


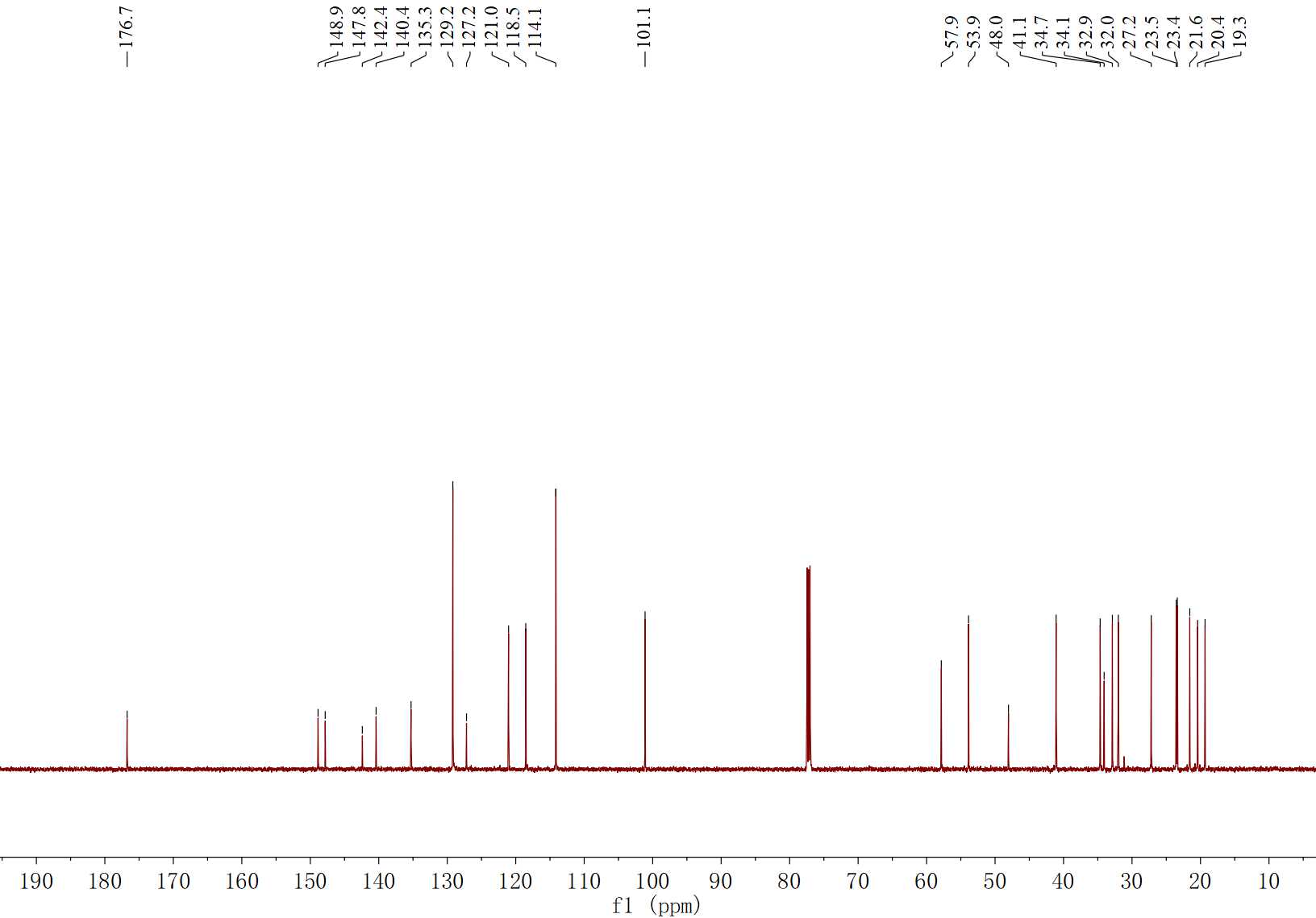


**Compound 5b: ^1^H NMR**


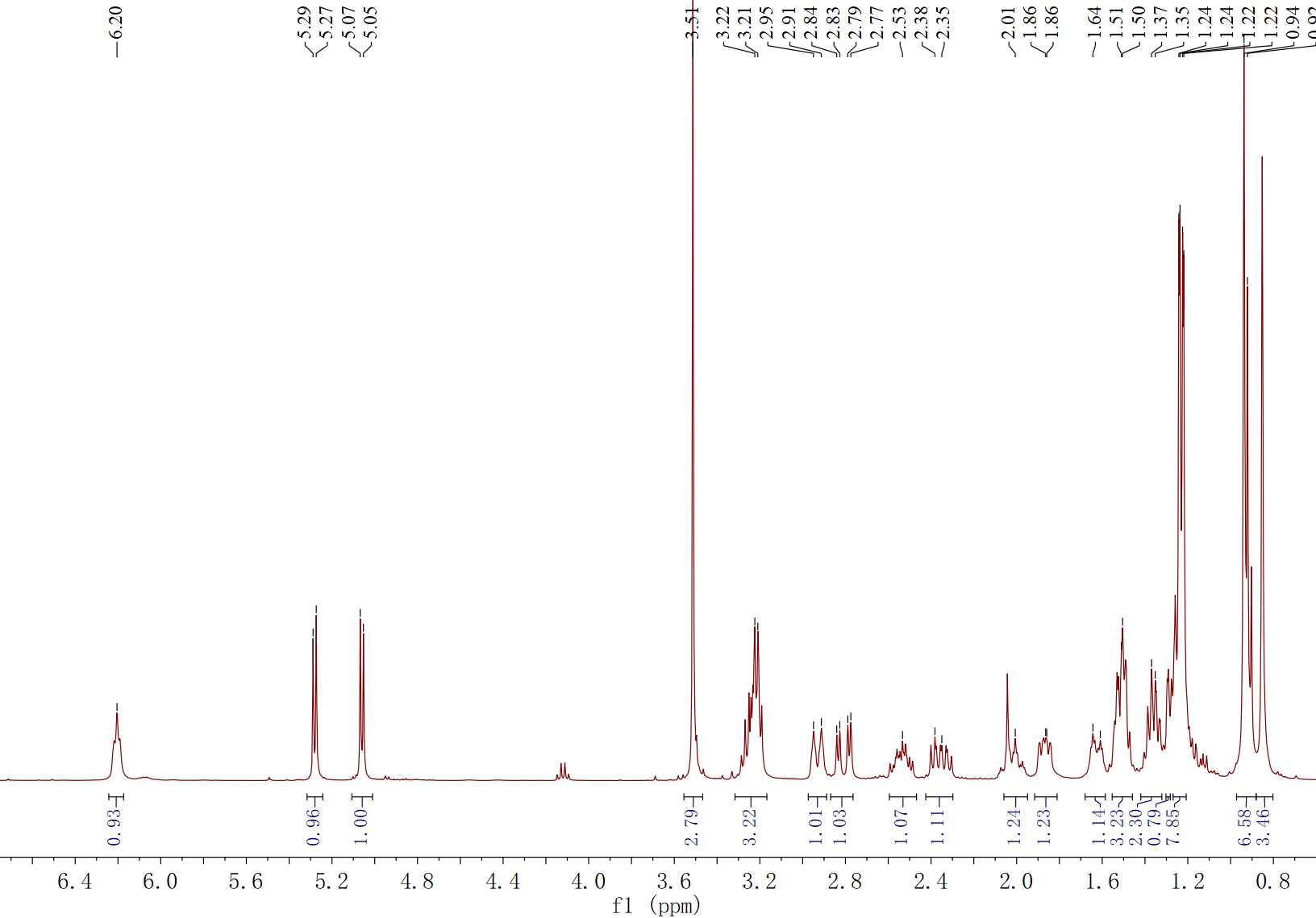


**Compound 5b: ^13^C NMR**


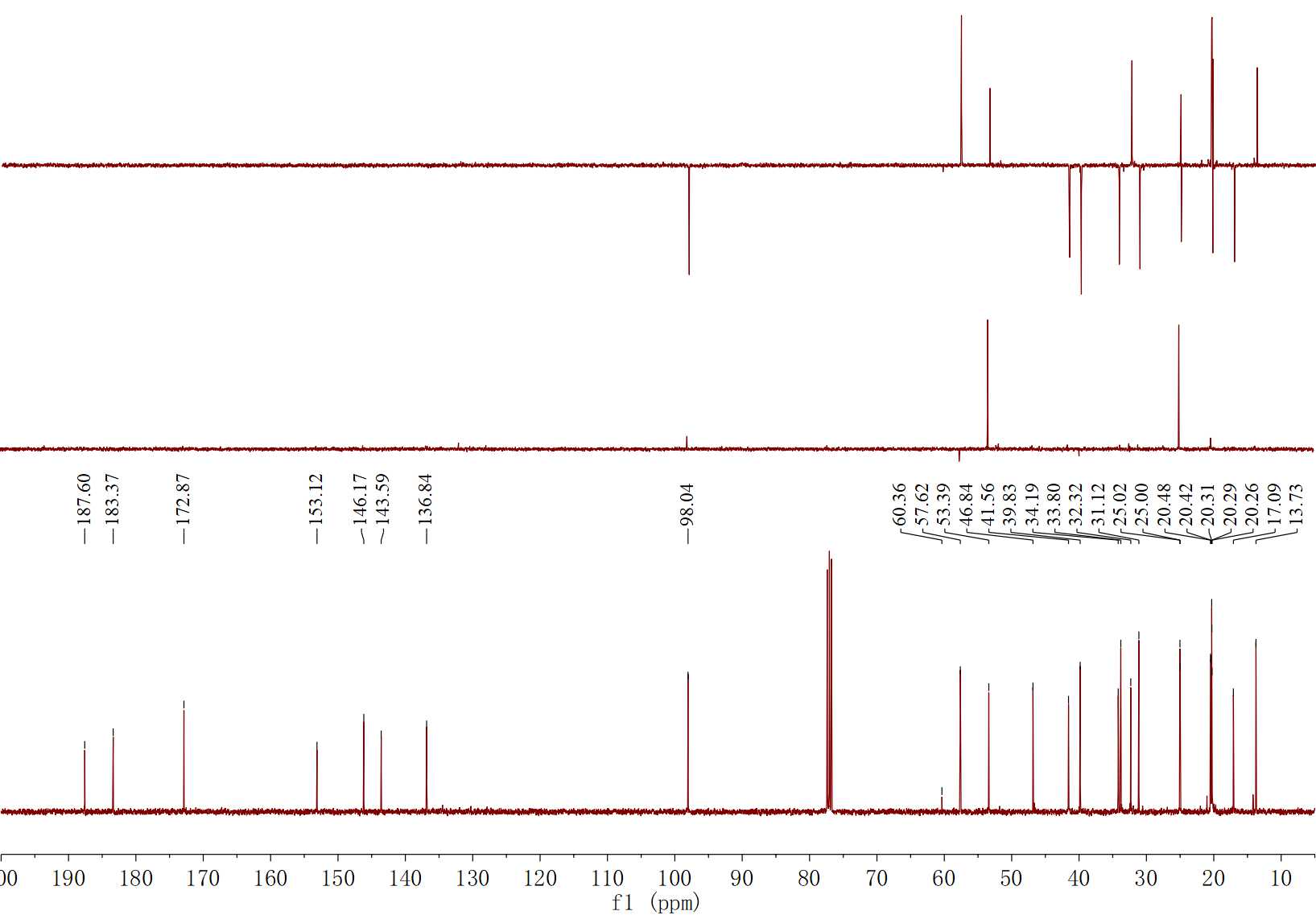


**Compound 5c: ^1^H NMR**


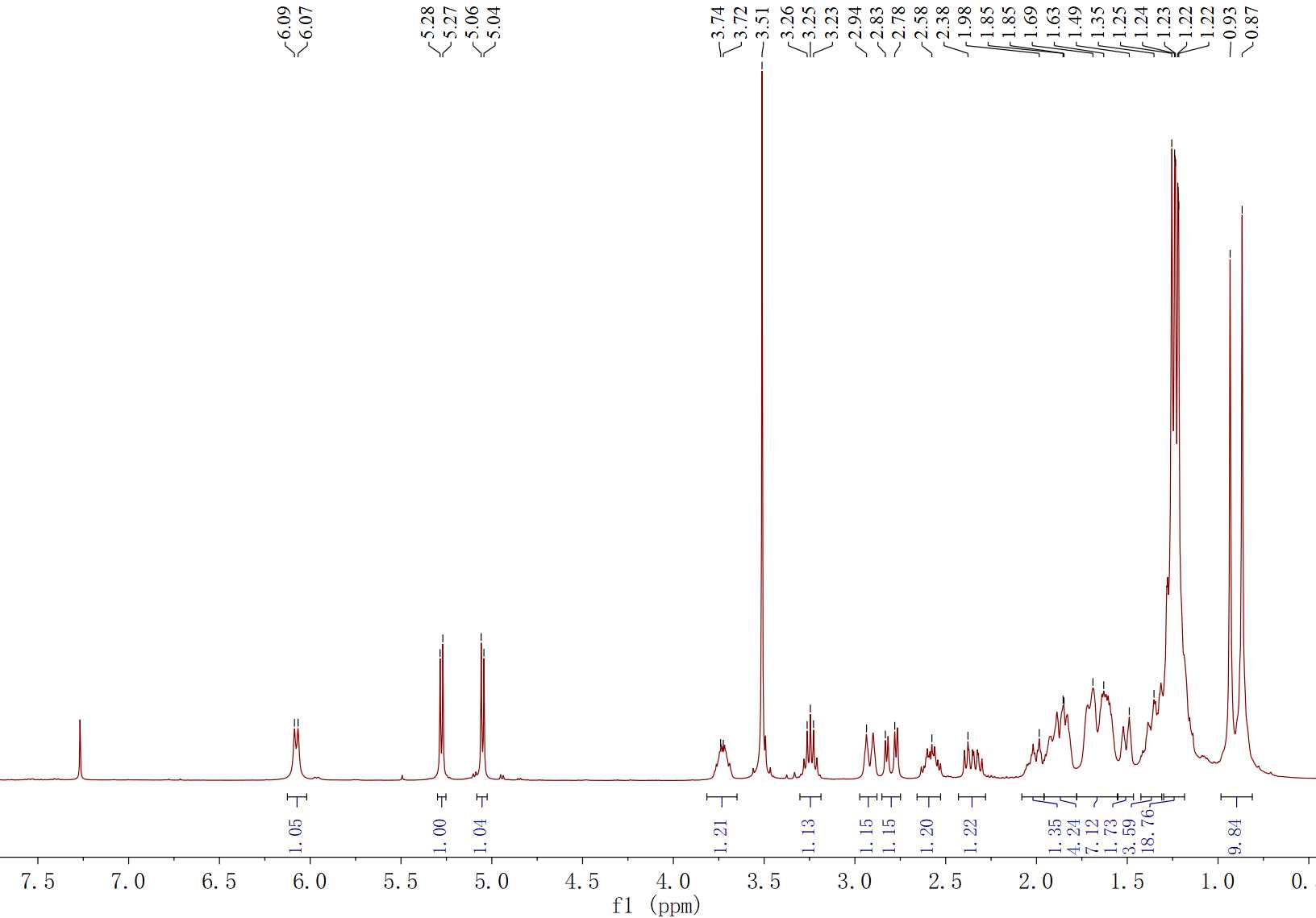


**Compound 5c: ^1^H NMR**

**
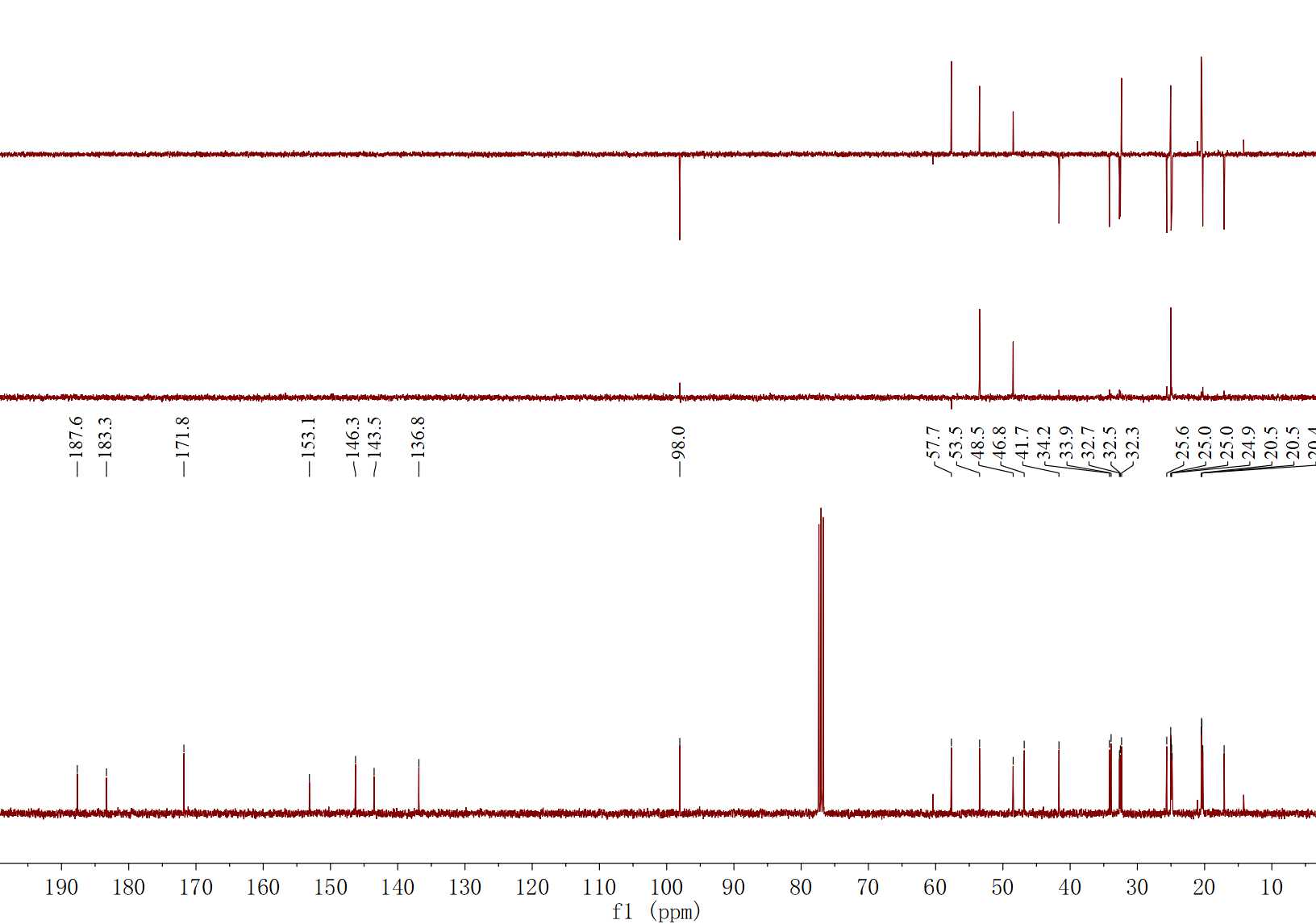
**

**Compound 5d: ^1^H NMR**


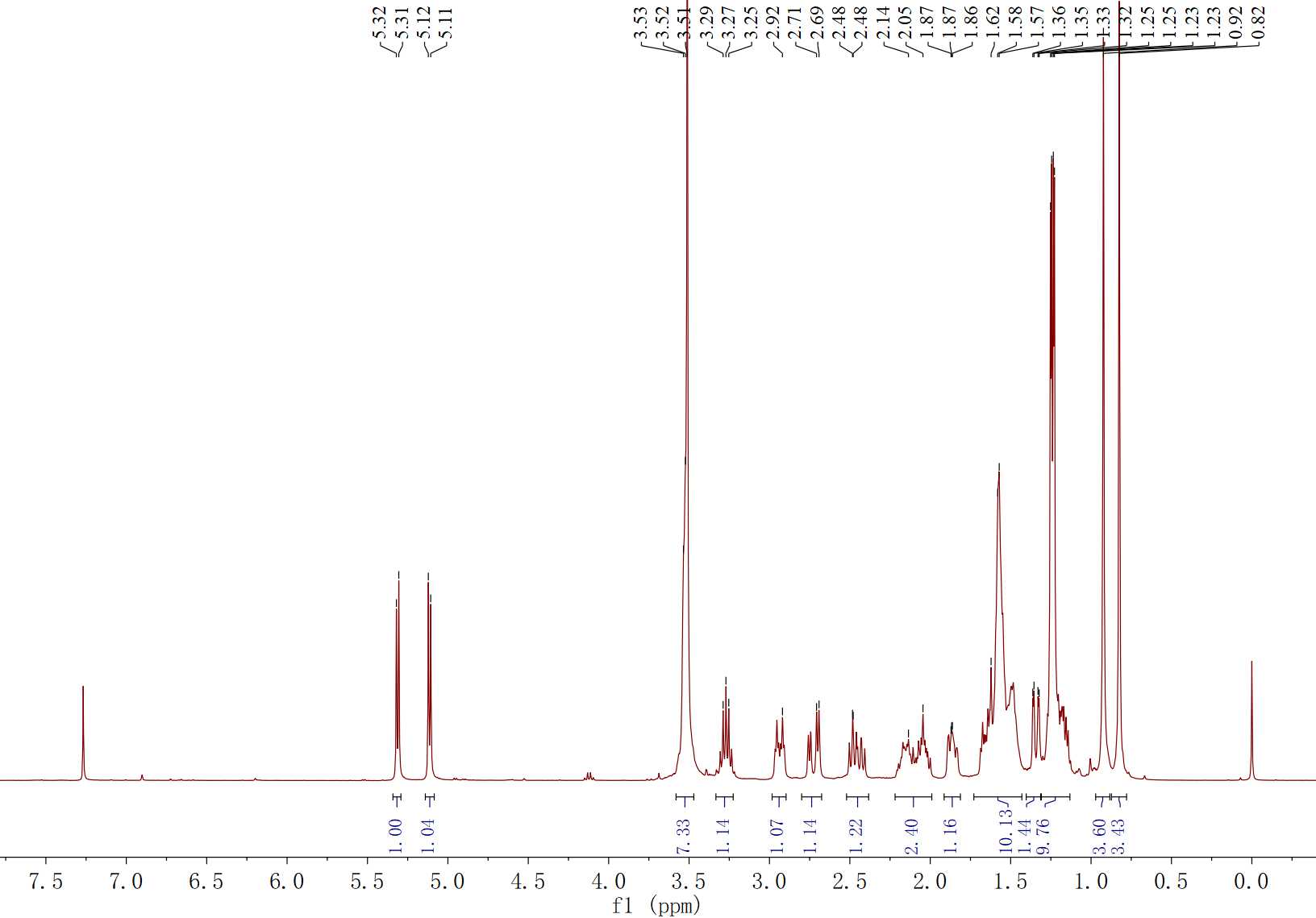


**Compound 5d: ^13^C NMR**


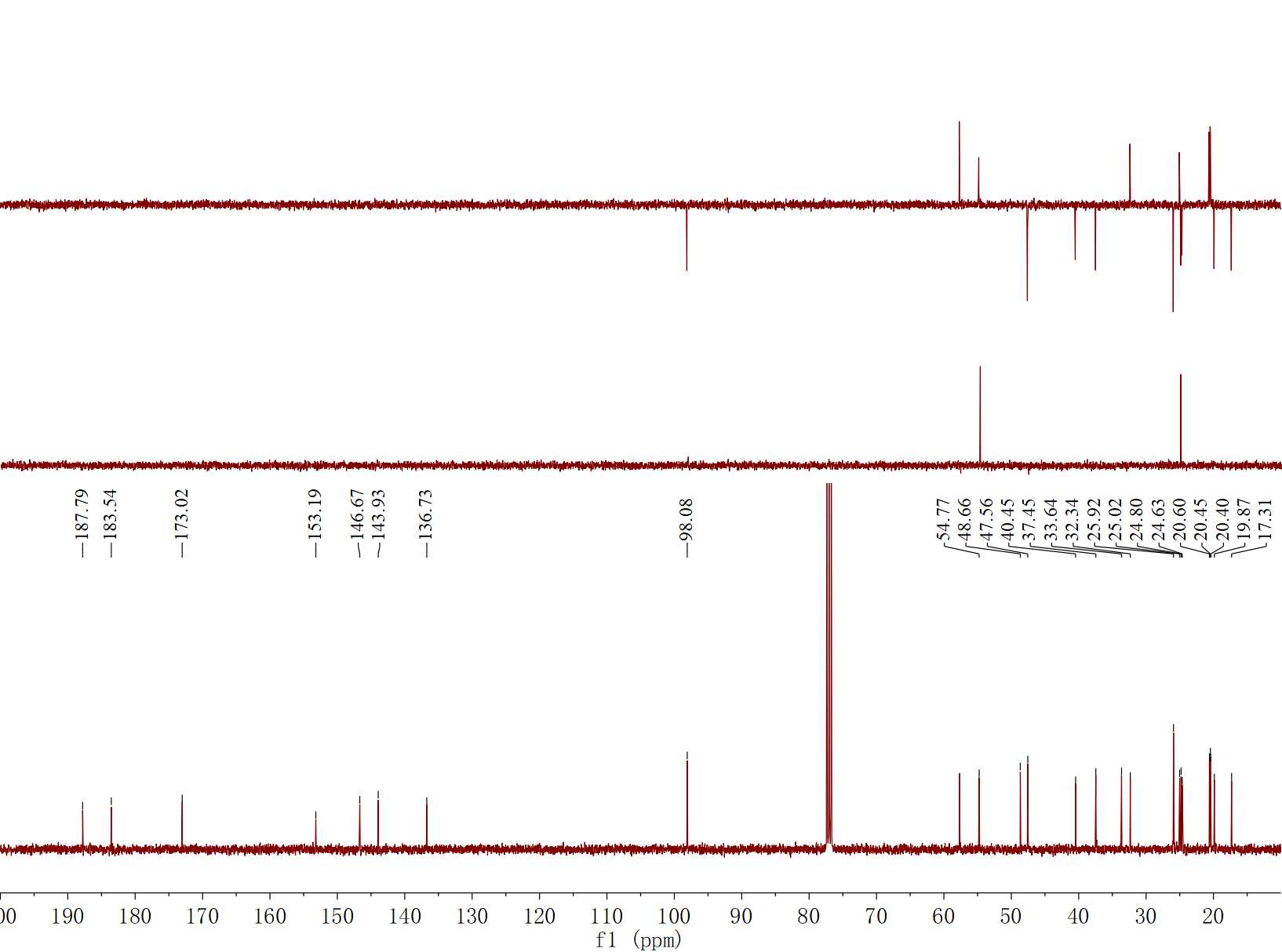


**Compound 6b: ^1^H NMR**


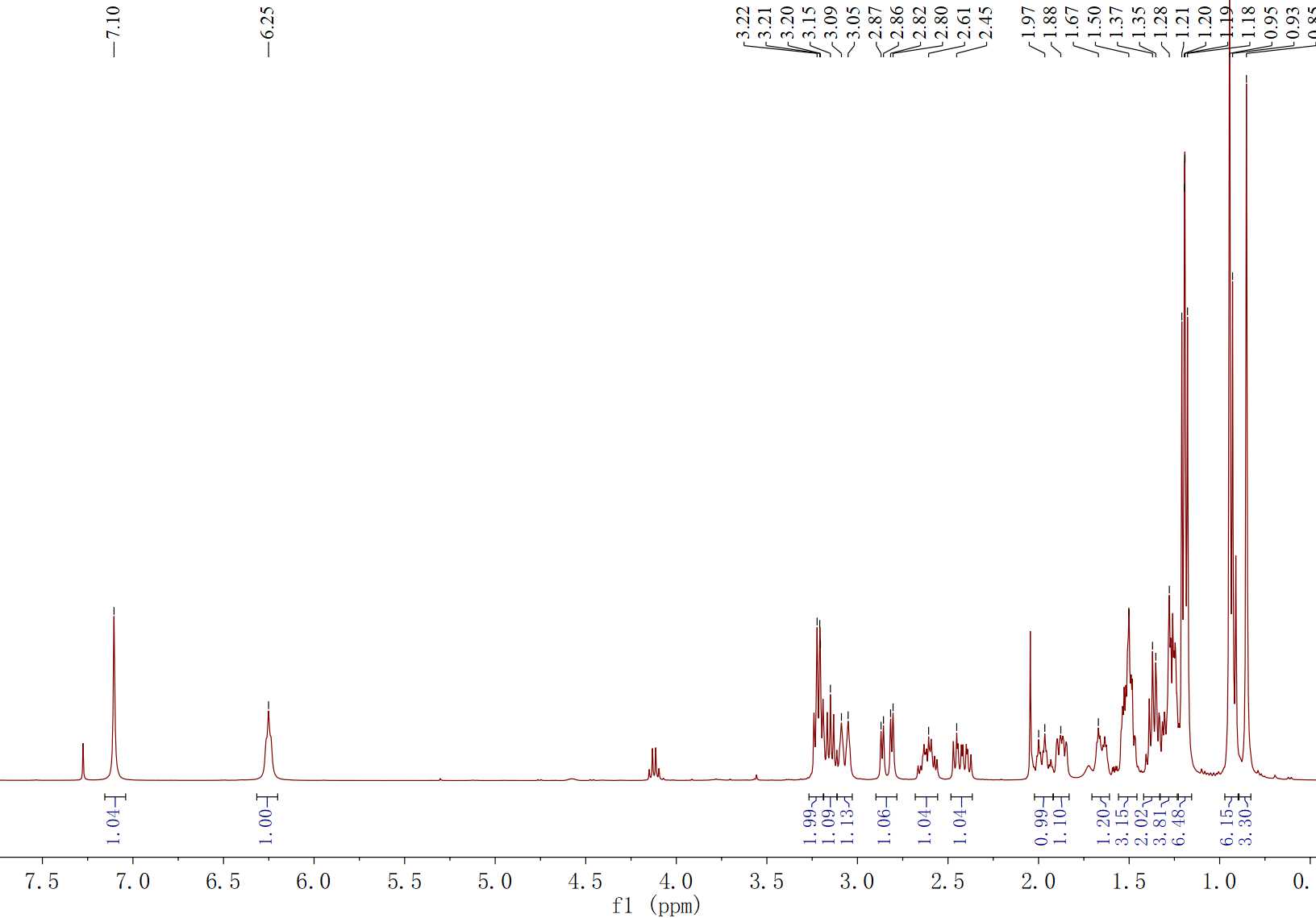


**Compound 6b: ^13^C NMR**


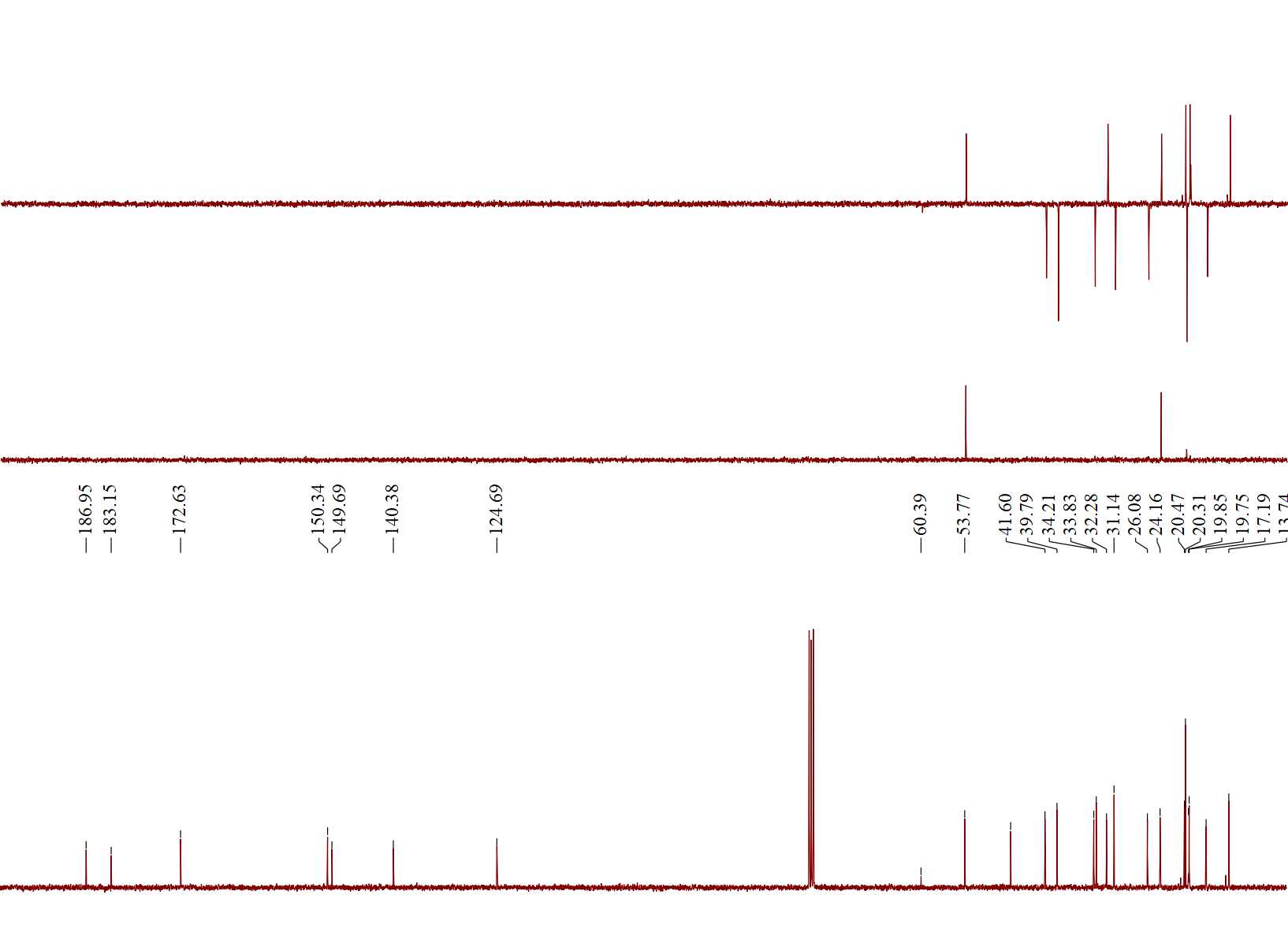


**Compound 6c: ^1^H NMR**


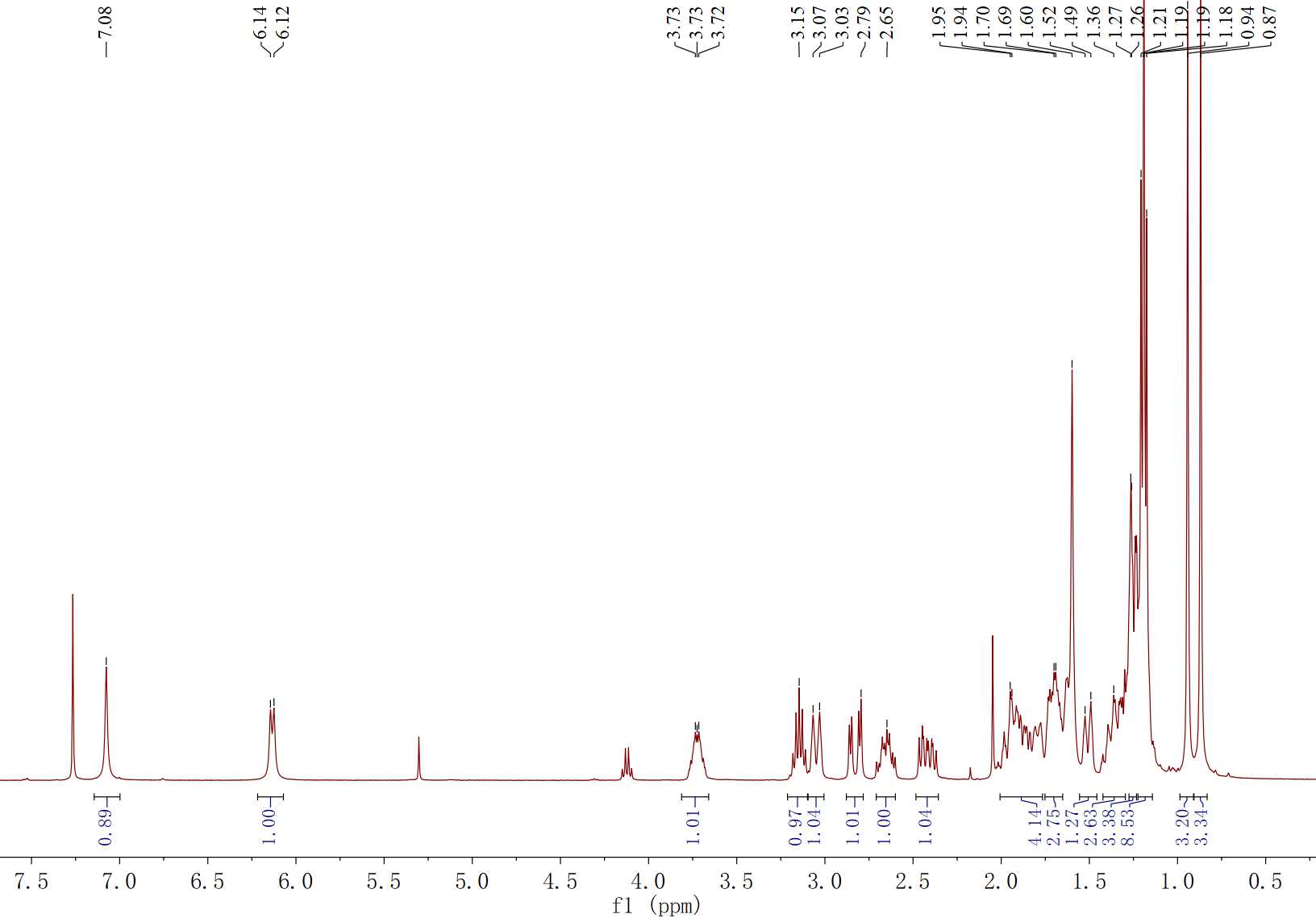


**Compound 6c: ^13^C NMR**


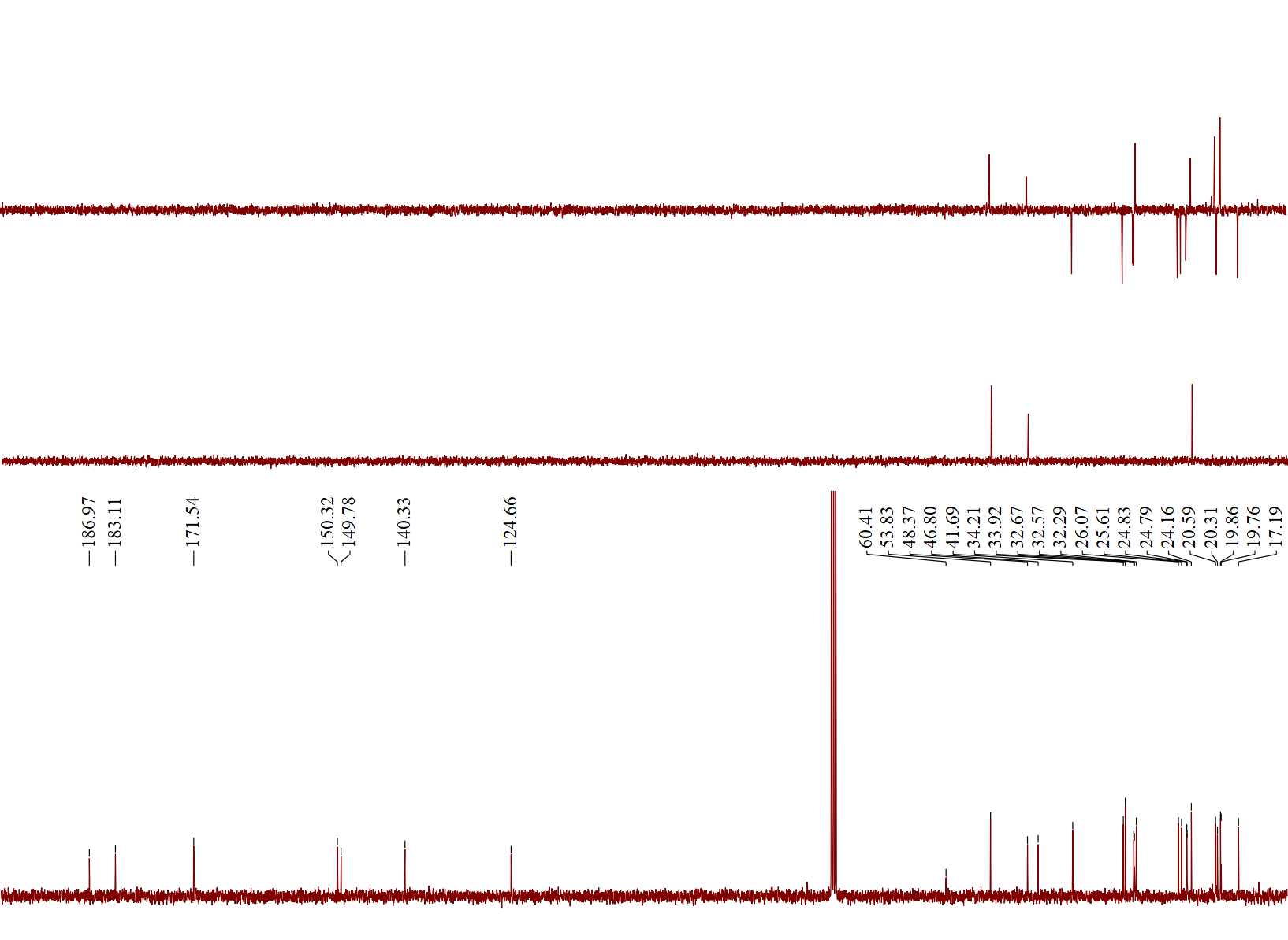


**Compound 6d: ^1^H NMR**


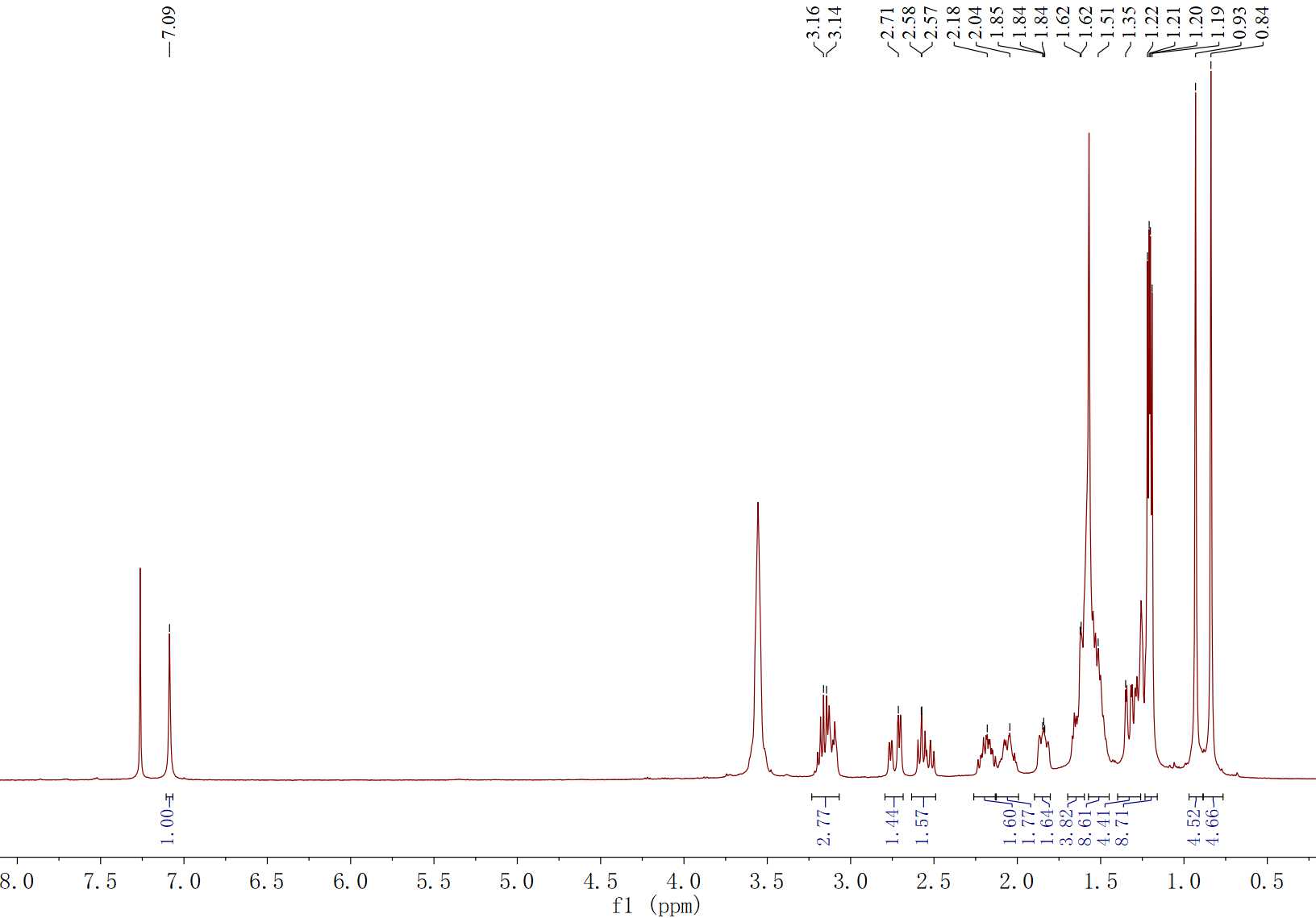


**Compound 6d: ^13^C NMR**


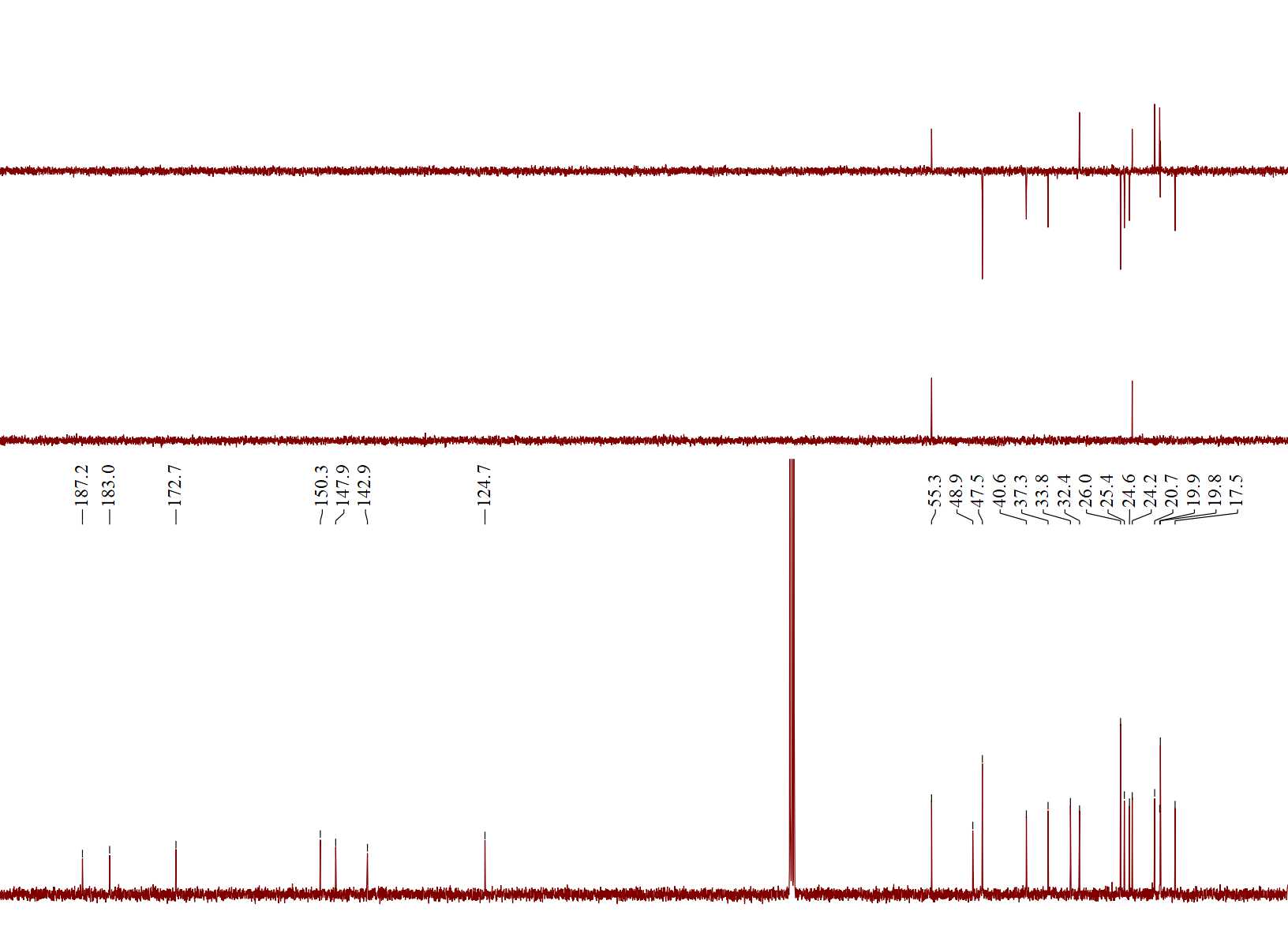


**Compound 6e: ^1^H NMR**


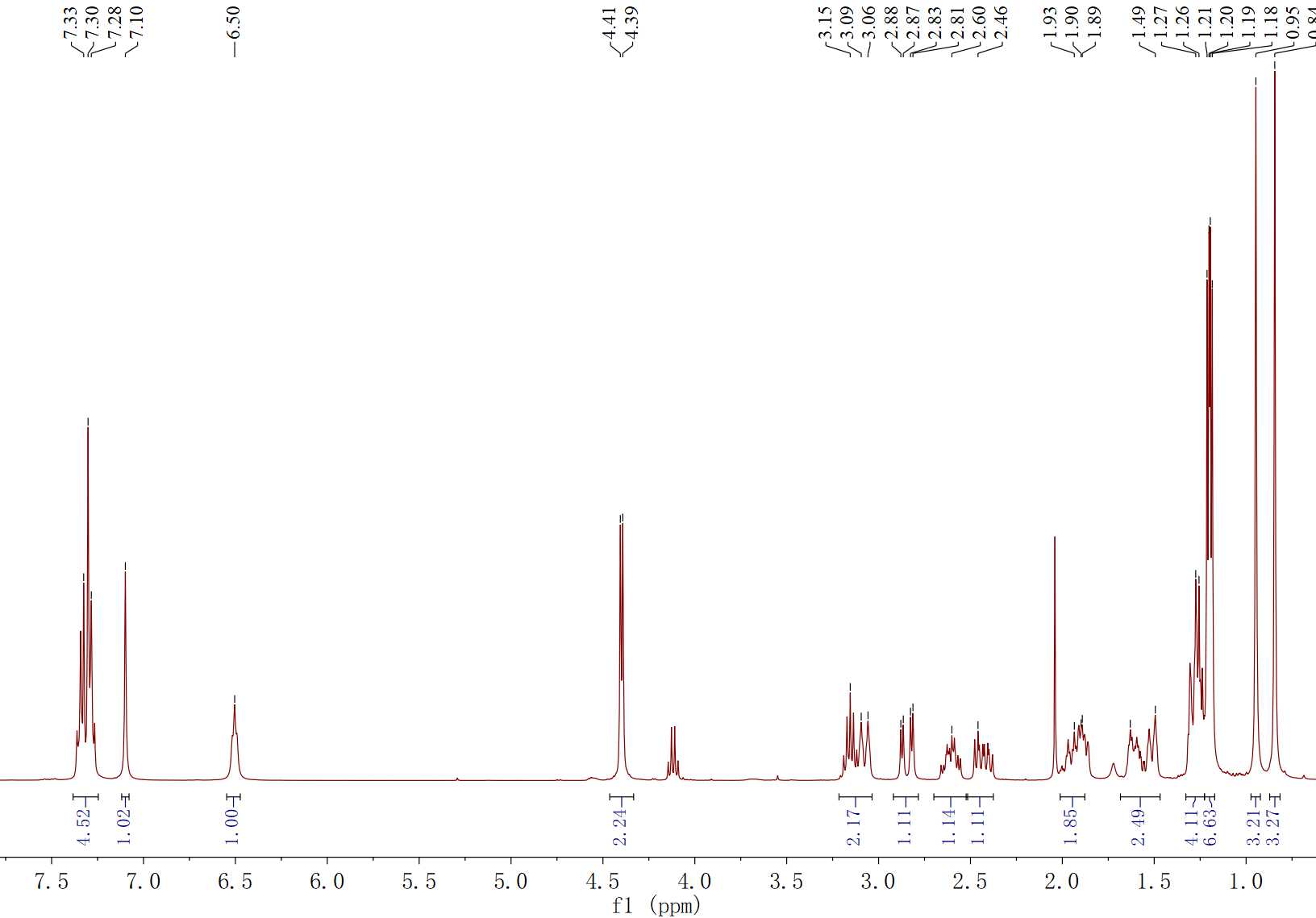


**Compound 6e: ^13^C NMR**


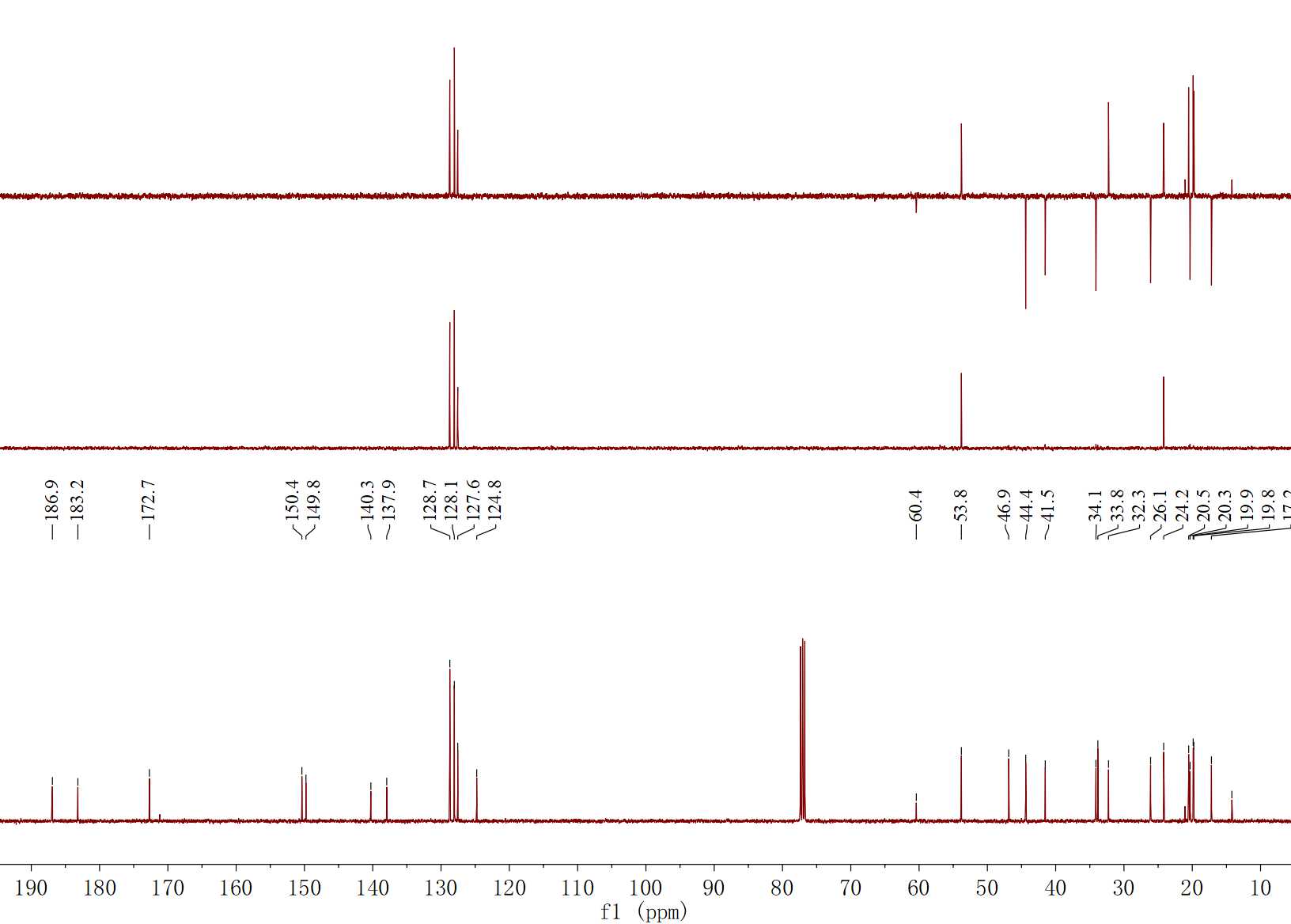


**Compound 6g: ^1^H NMR**


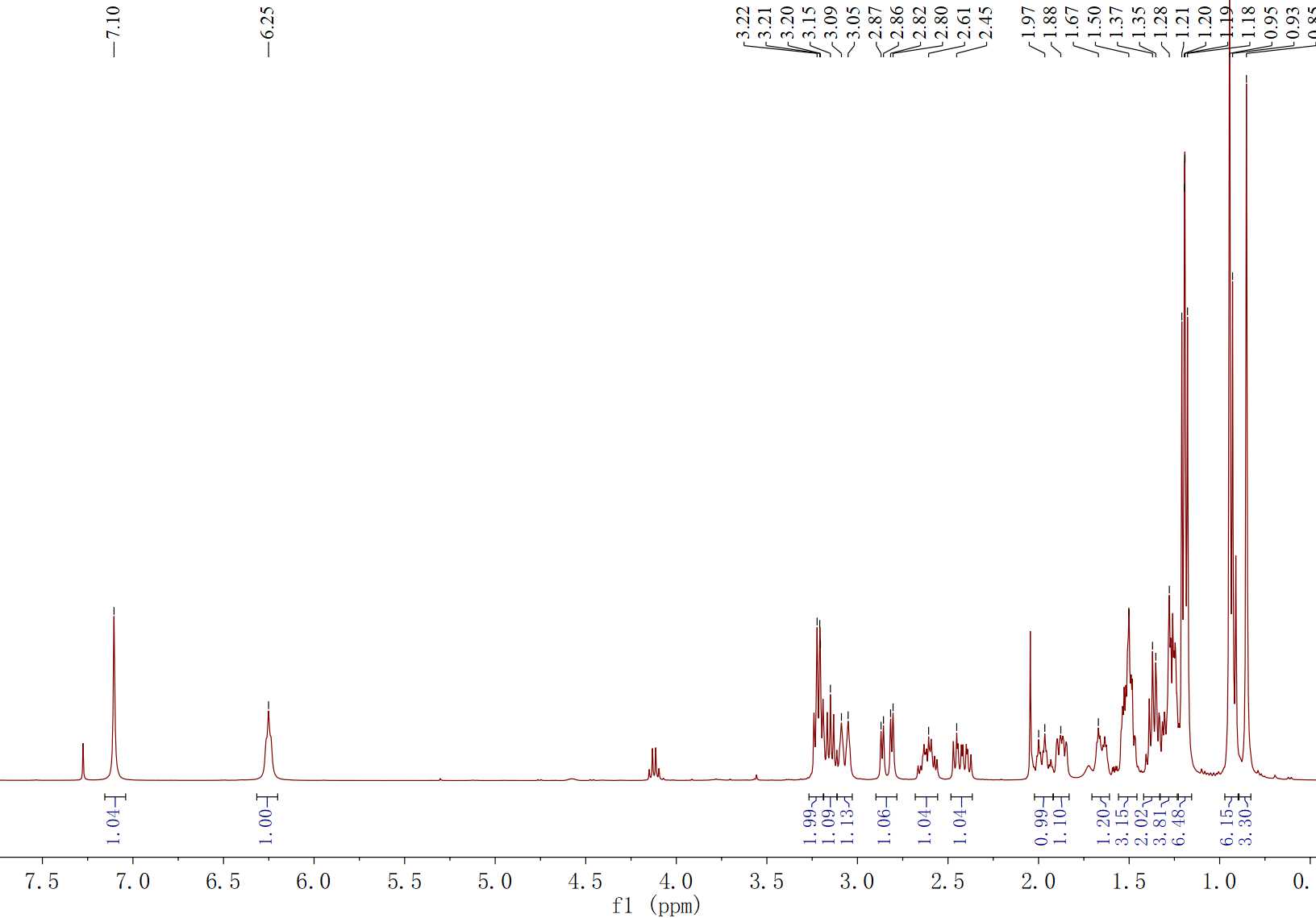


**Compound 6g: ^13^C NMR**


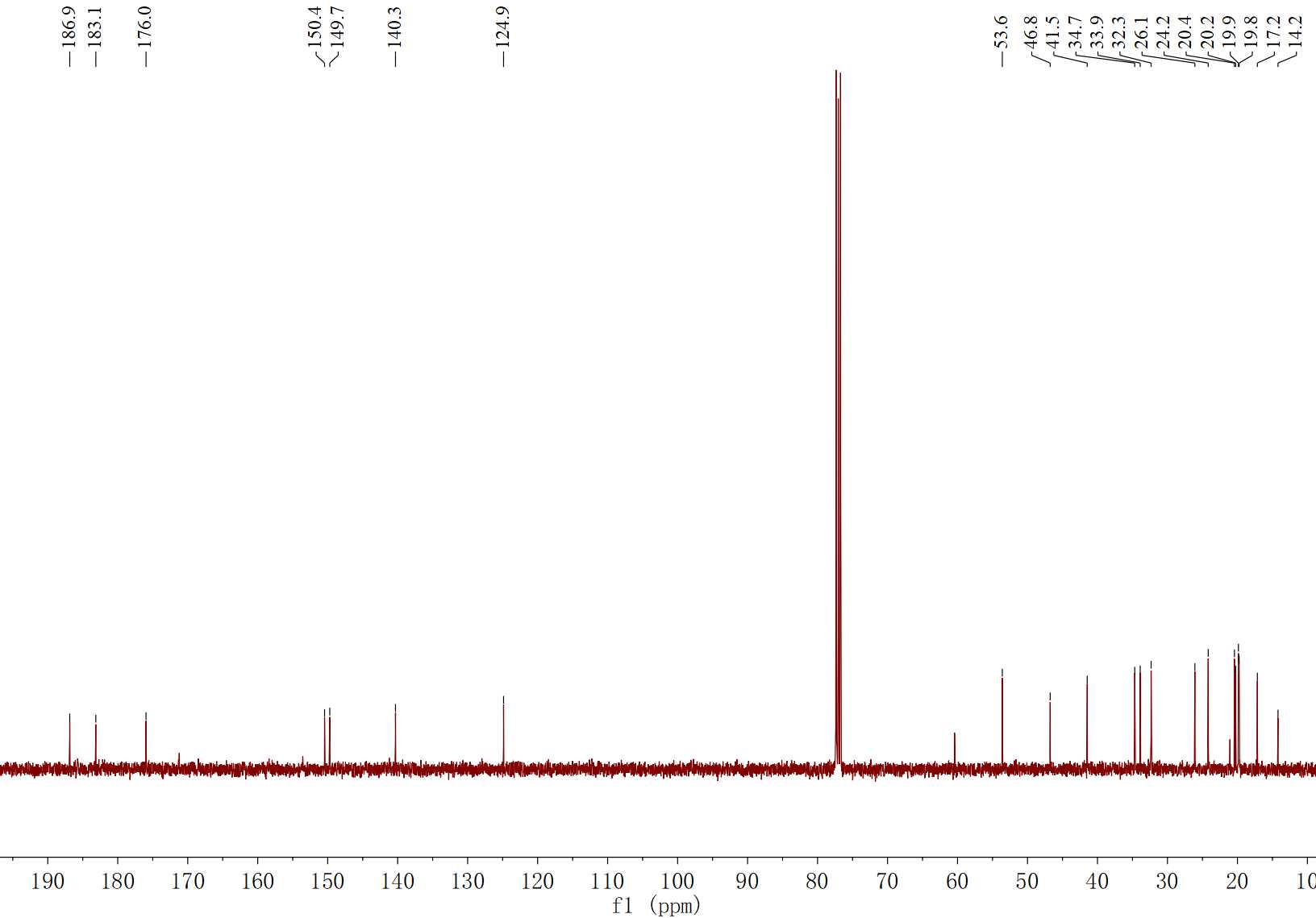


**Compound 7: ^1^H NMR**


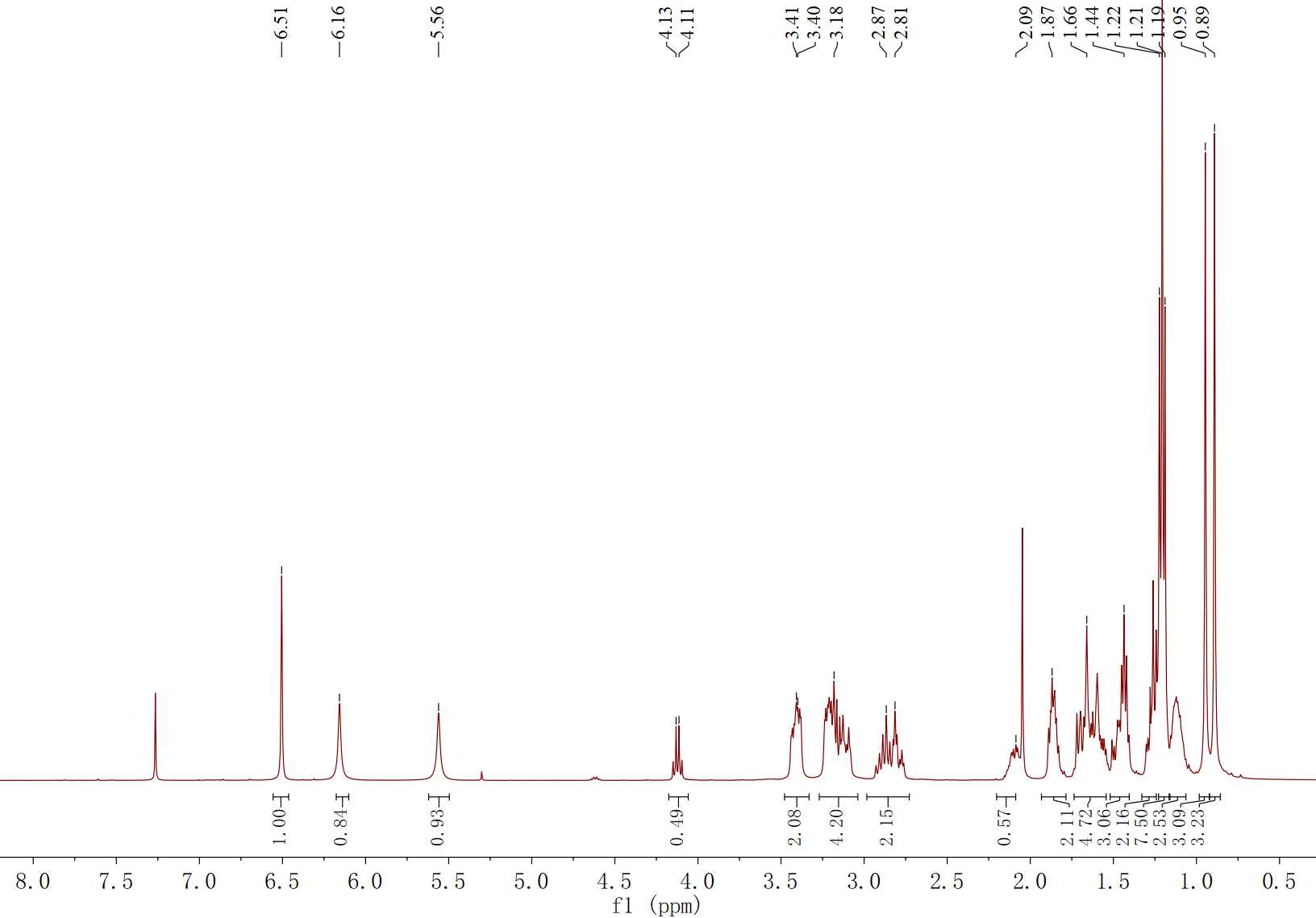


**Compound 7: ^13^C NMR**


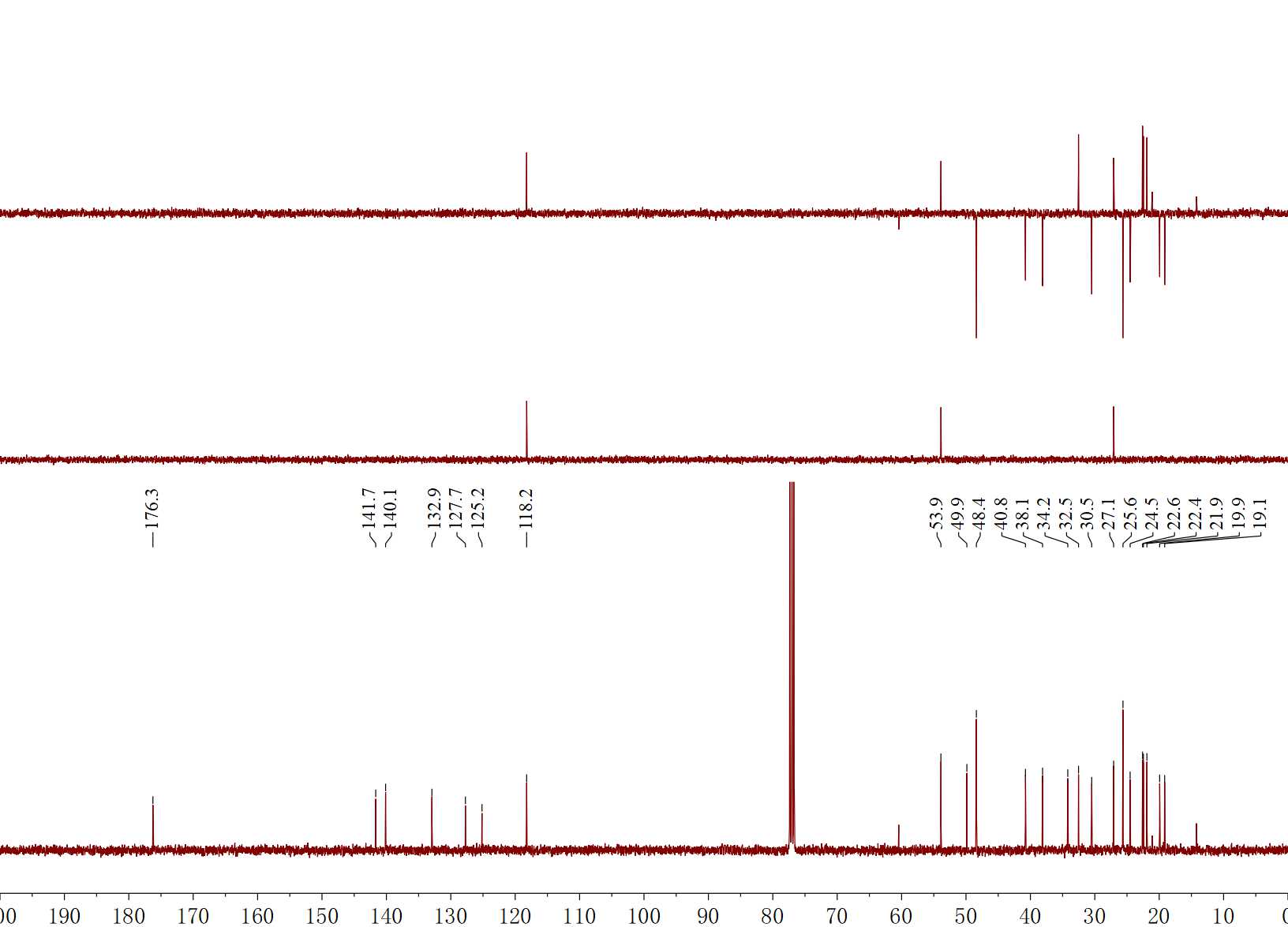


**Compound 8: ^1^H NMR**

**
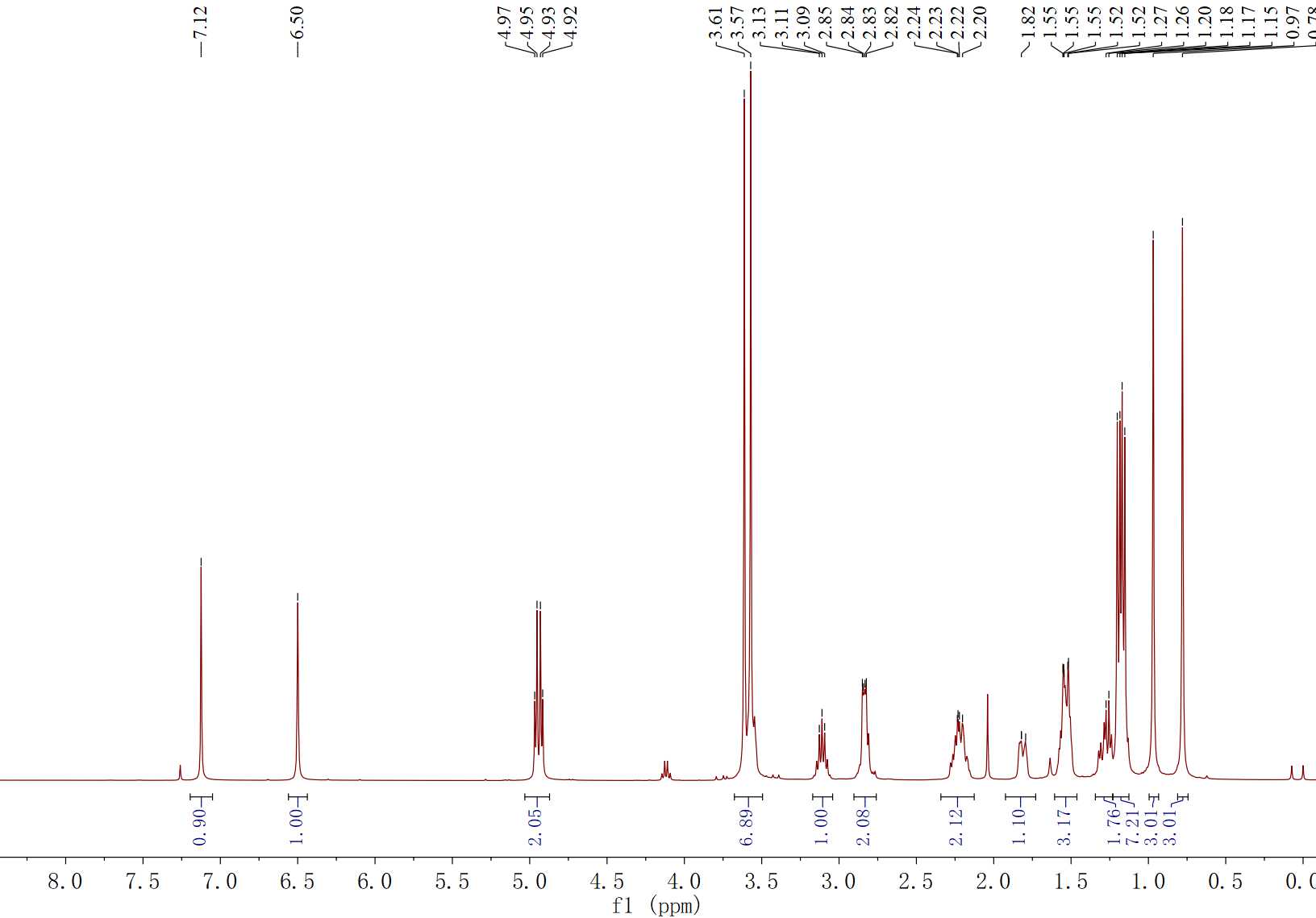
**

**Compound 8: ^13^C NMR**

**
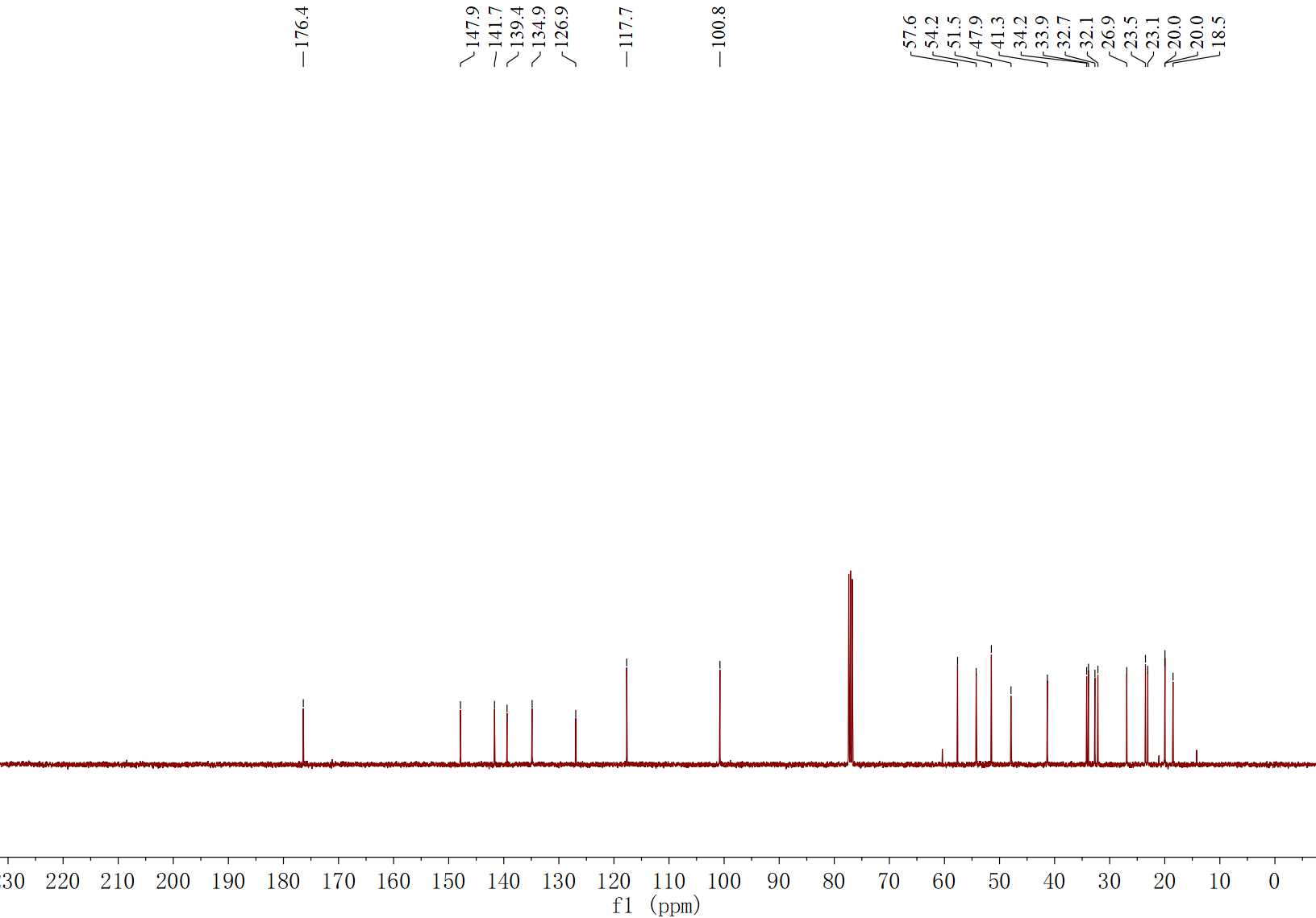
**

**Compound 9: ^1^H NMR**


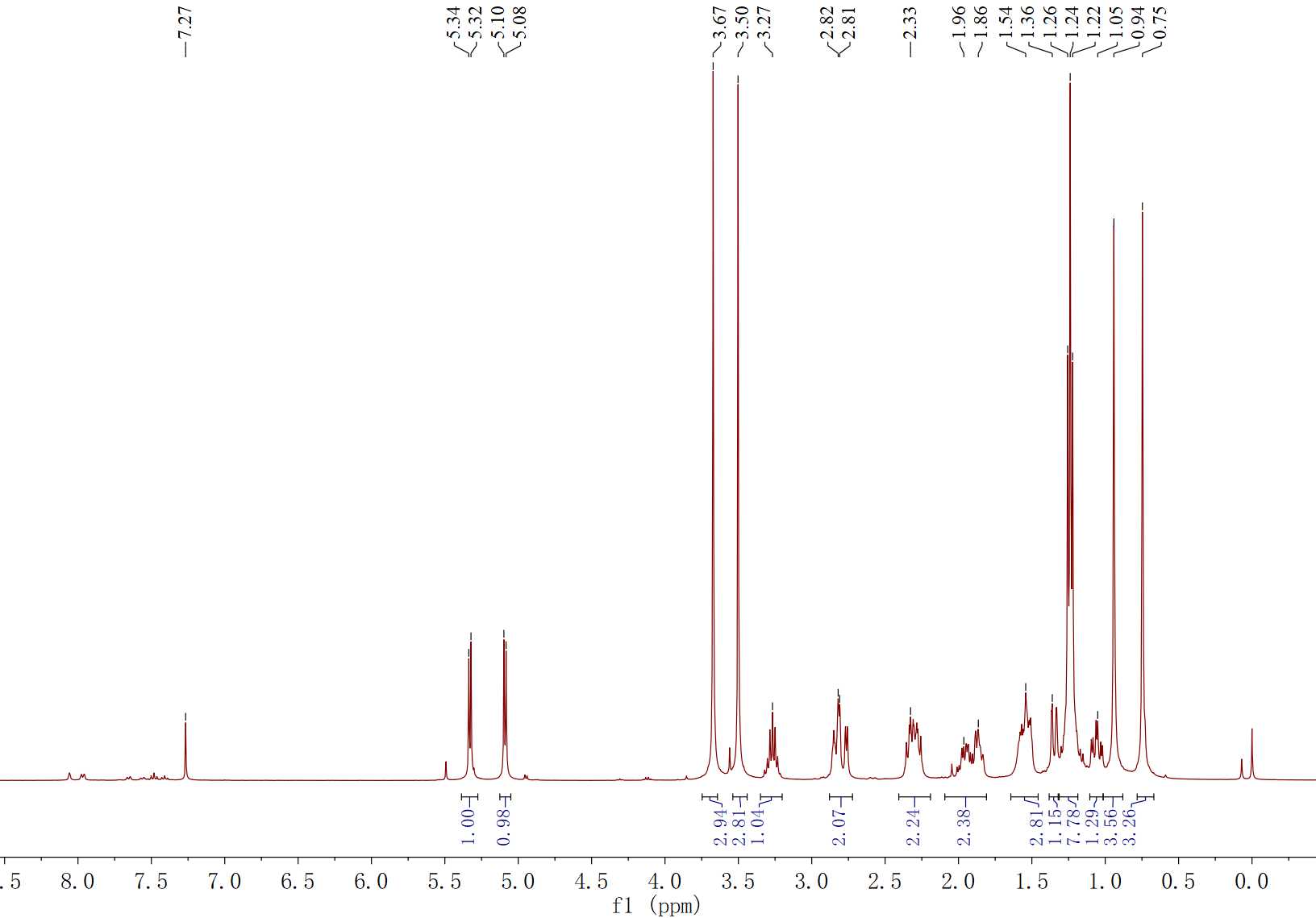


**Compound 9: ^13^C NMR**


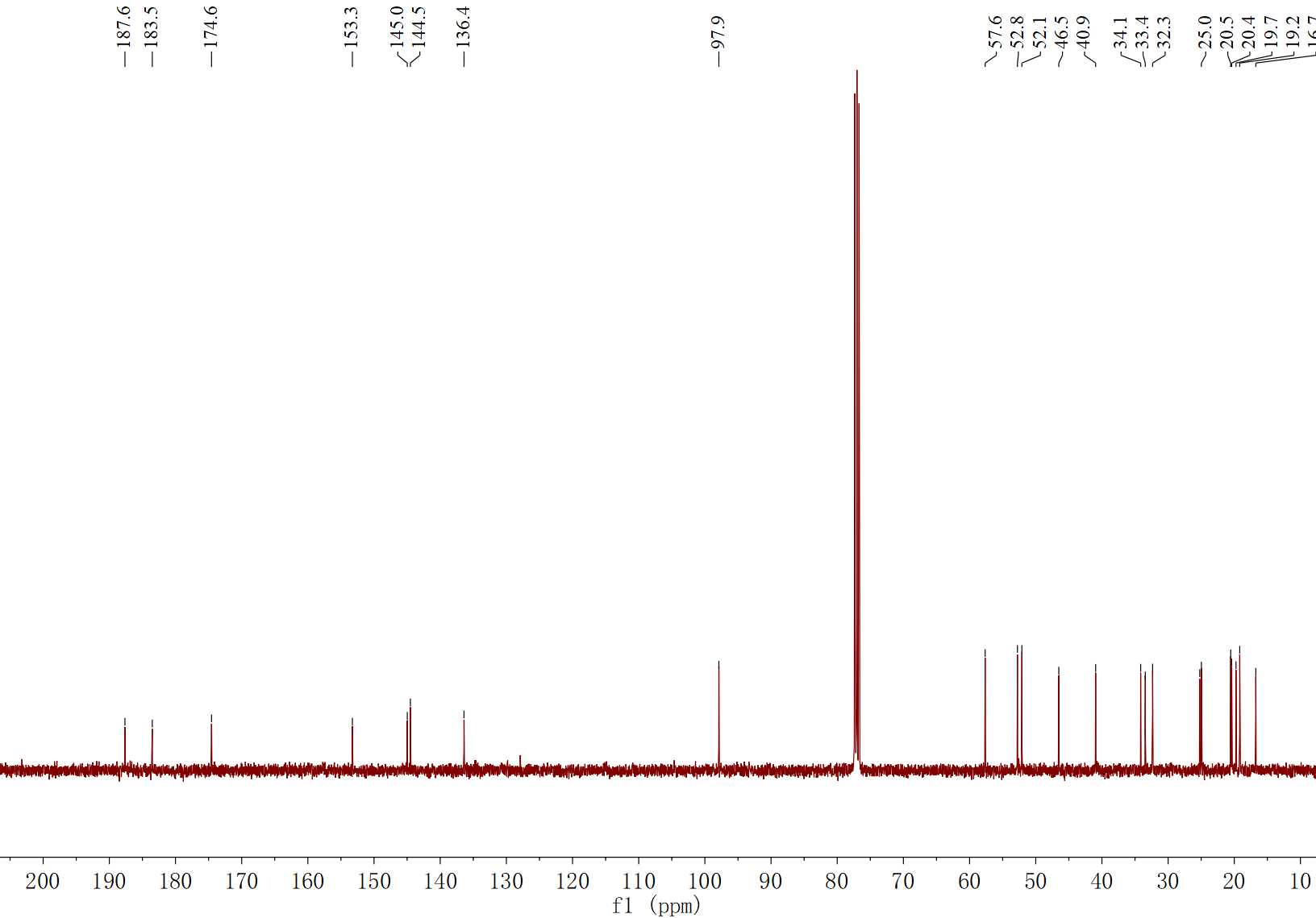


**Compound 10: ^1^H NMR**

**
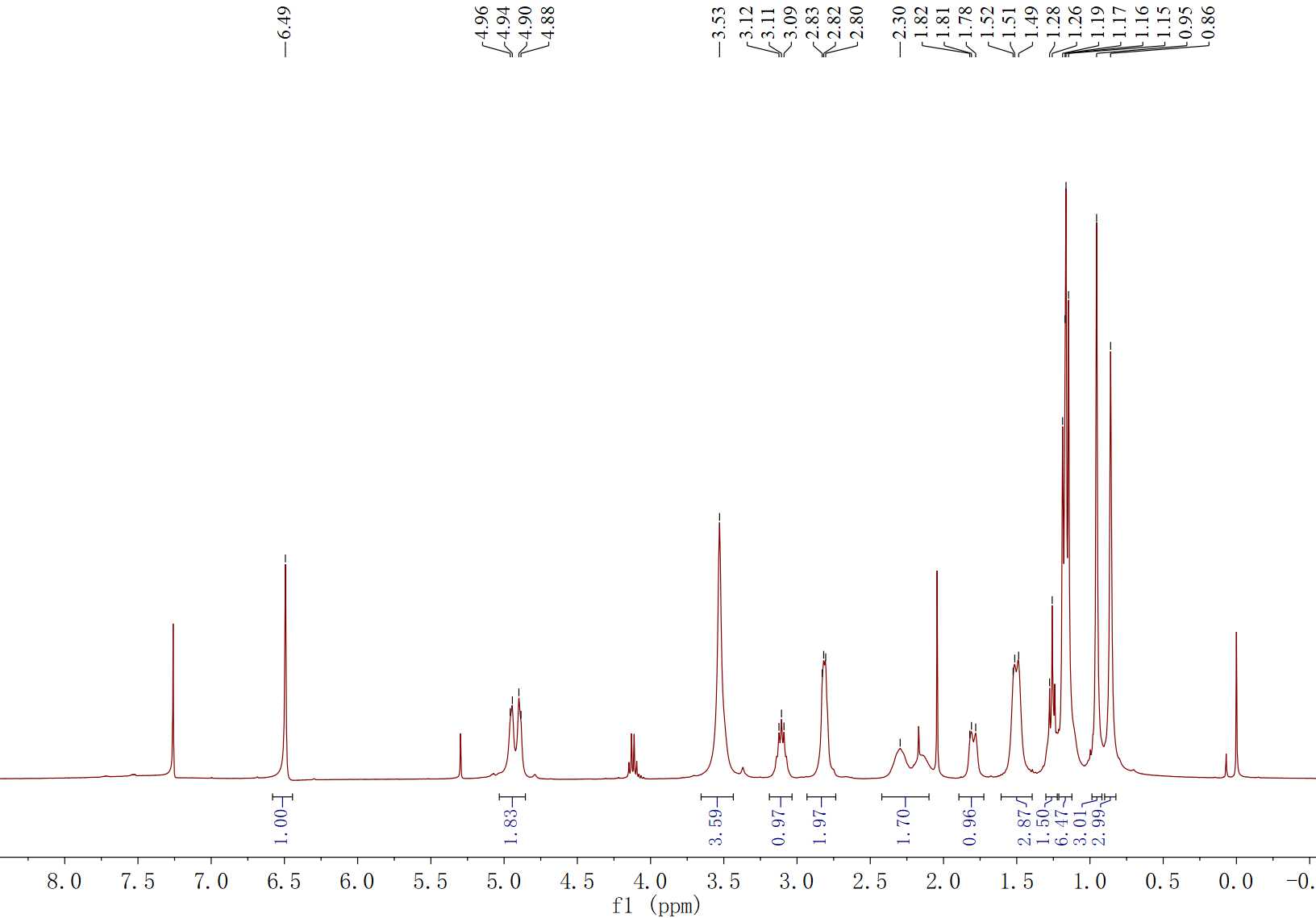
**

**Compound 10: ^13^C NMR**

**
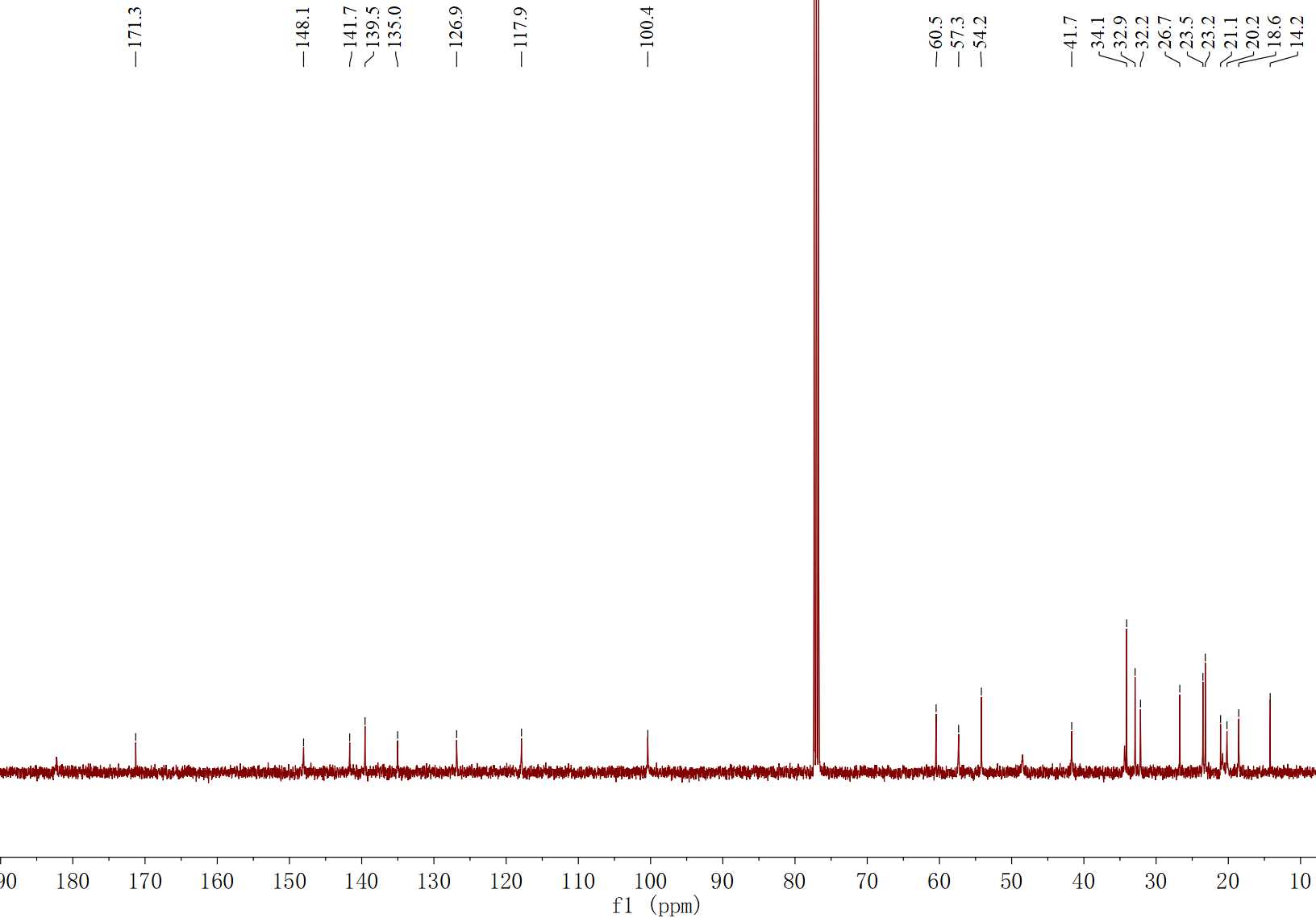
**

**Compound** **11**: **^1^H NMR**


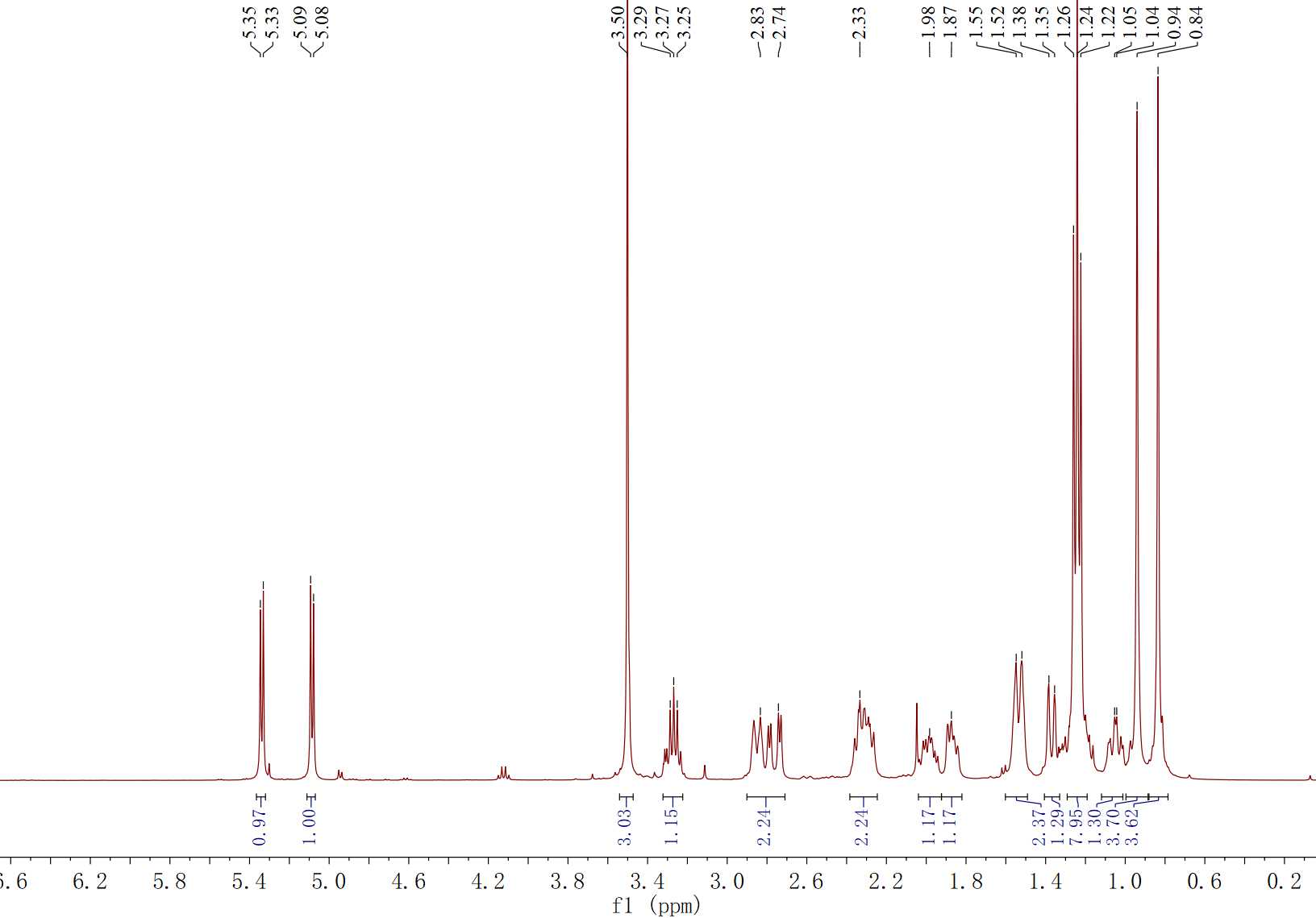


**Compound** **11**: **^13^C NMR**


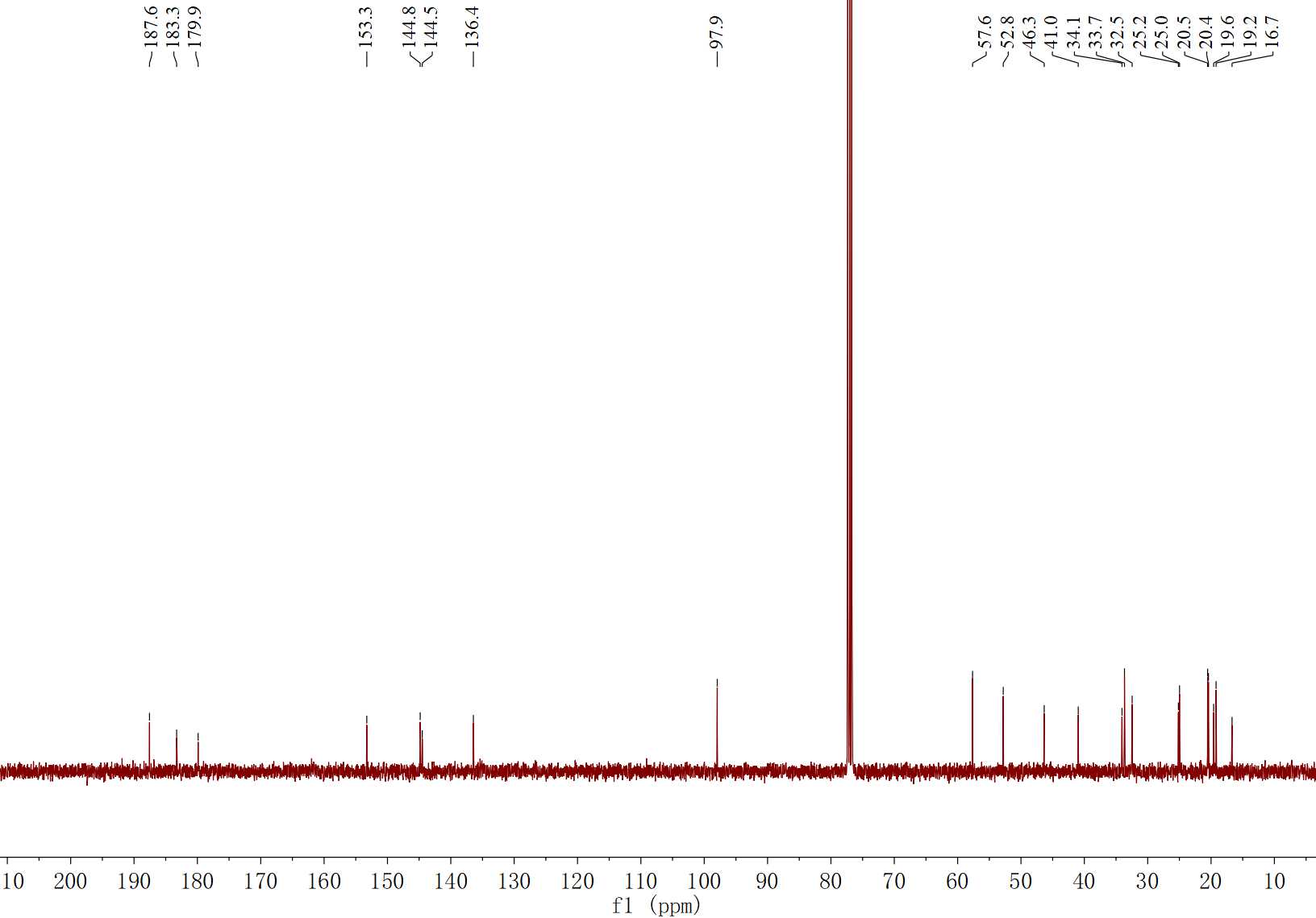


**Compound 12: ^1^H NMR**

**
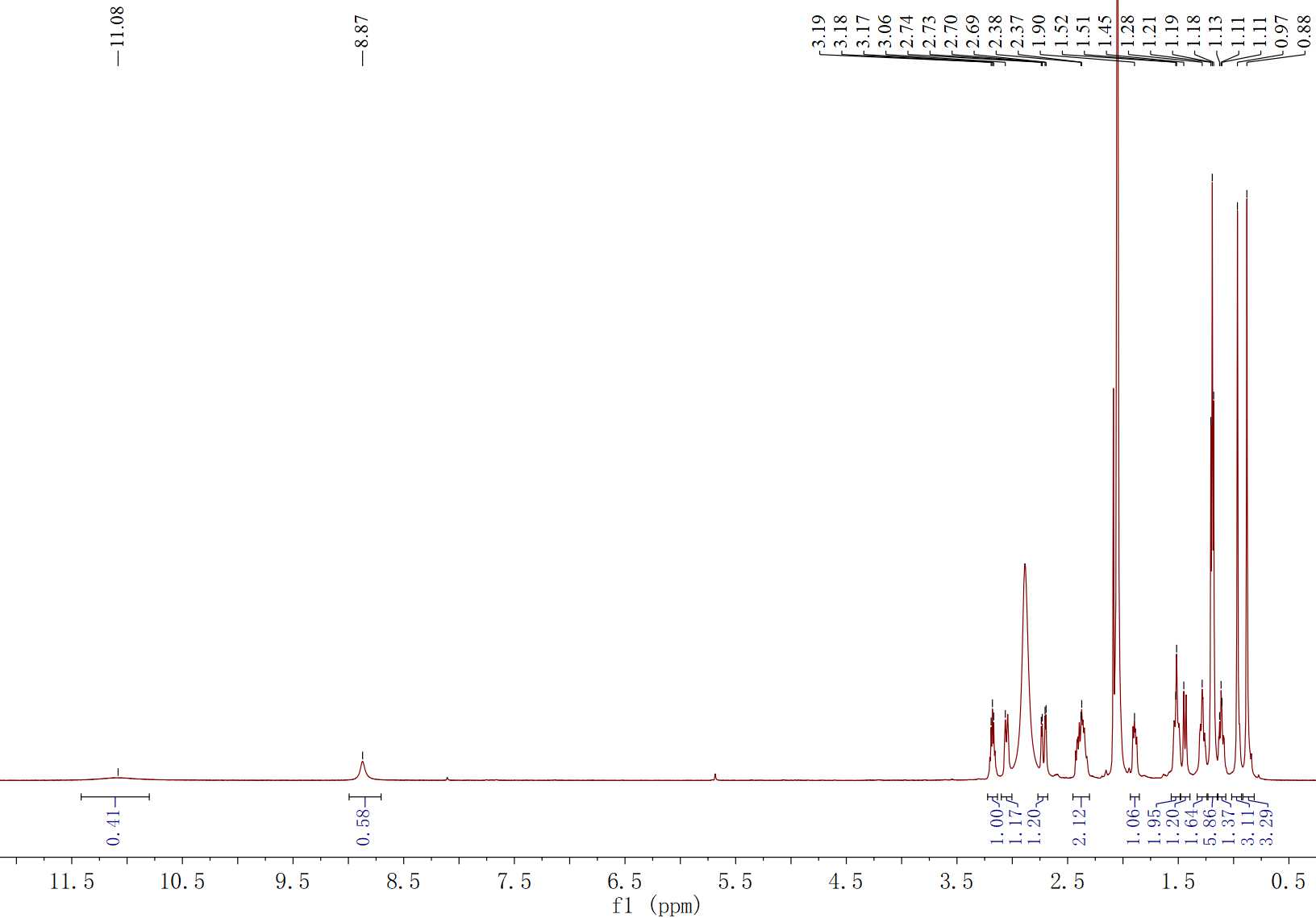
**

**Compound 12: ^13^C NMR**

**
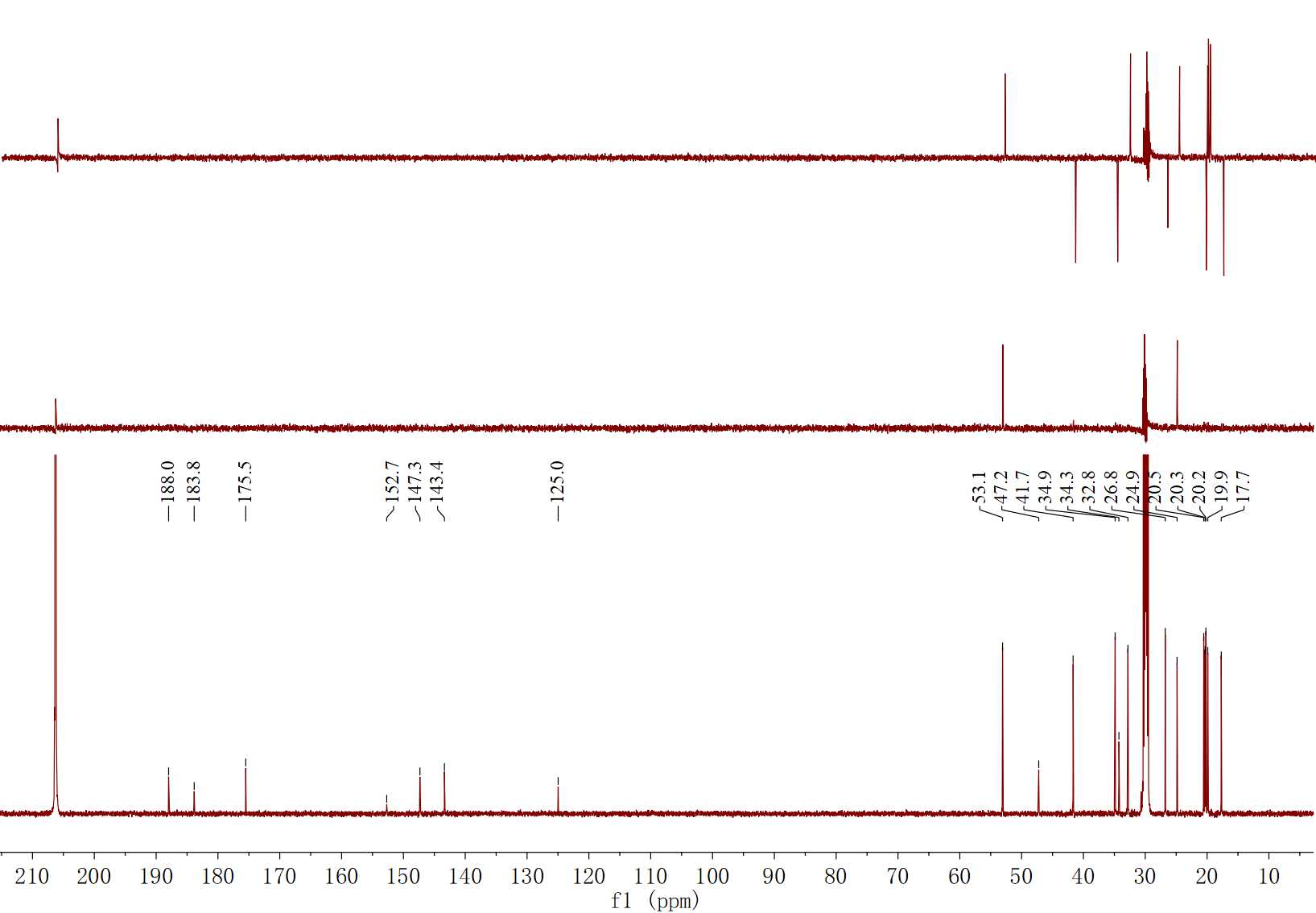
**

**Deoxyneocytotanshinone: ^1^H NMR**

**
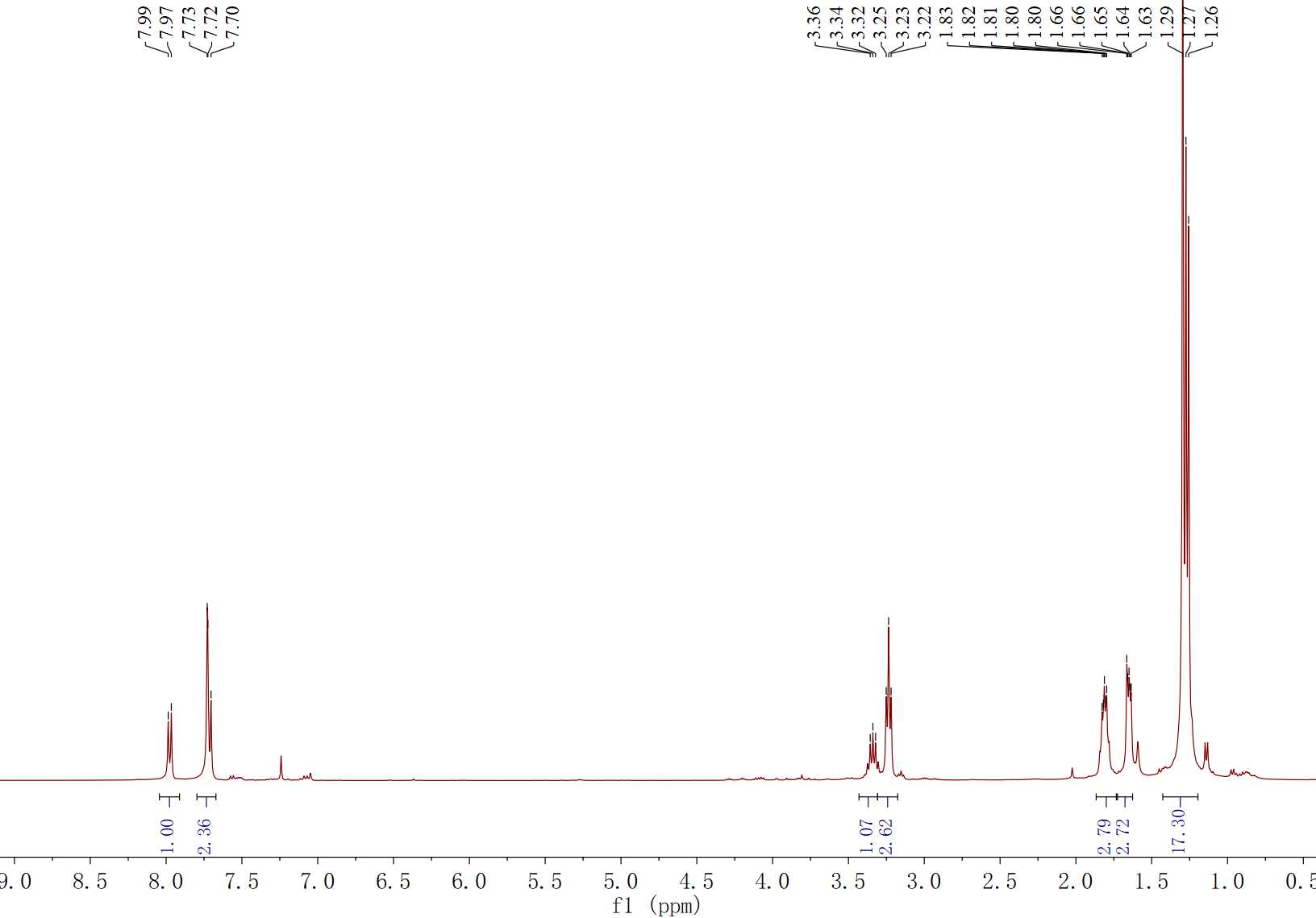
**

**Deoxyneocytotanshinone: ^13^C NMR**

**
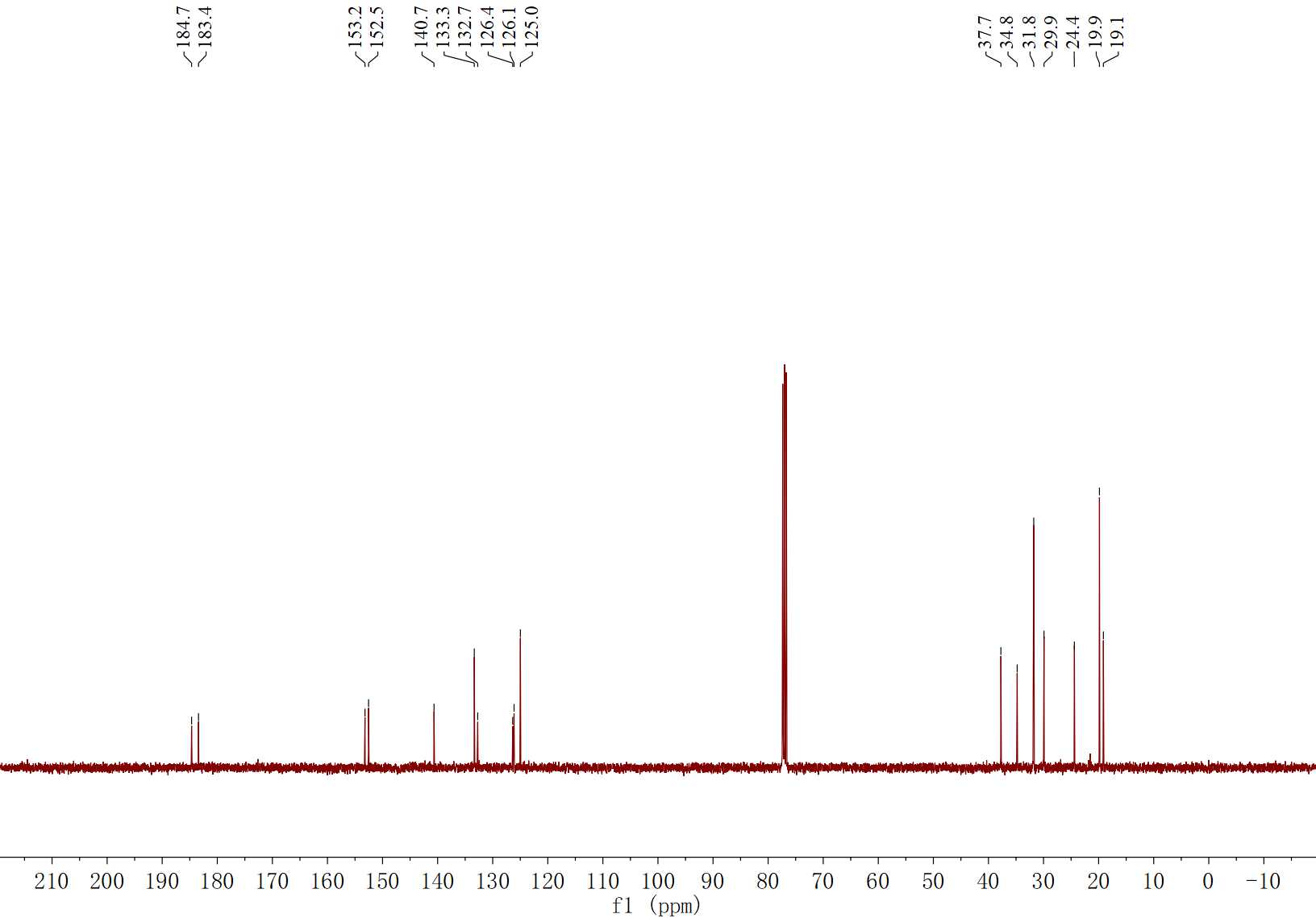
**

**Arucadiol: ^1^H NMR**

**
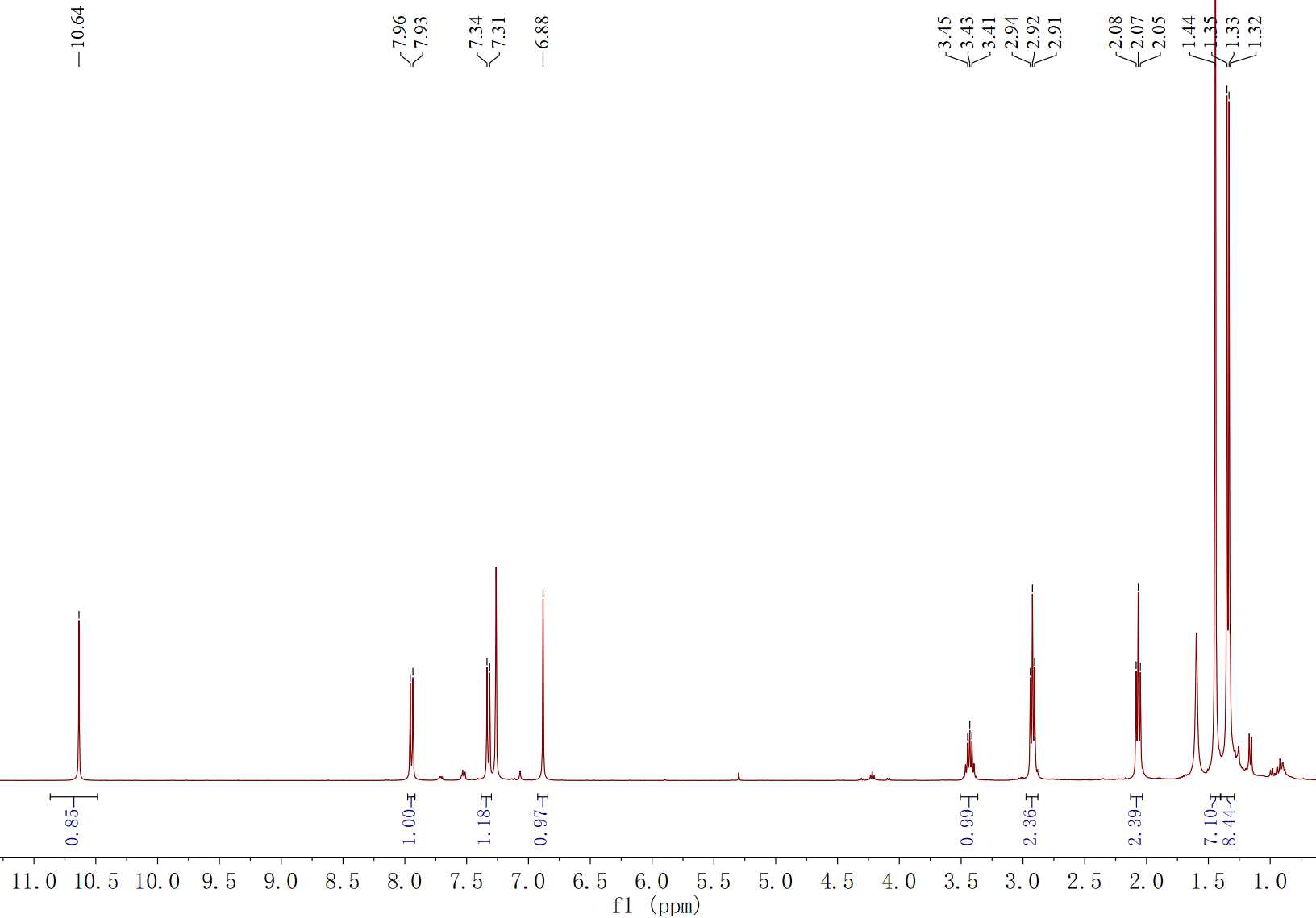
**

**Arucadiol: ^13^C NMR**

**
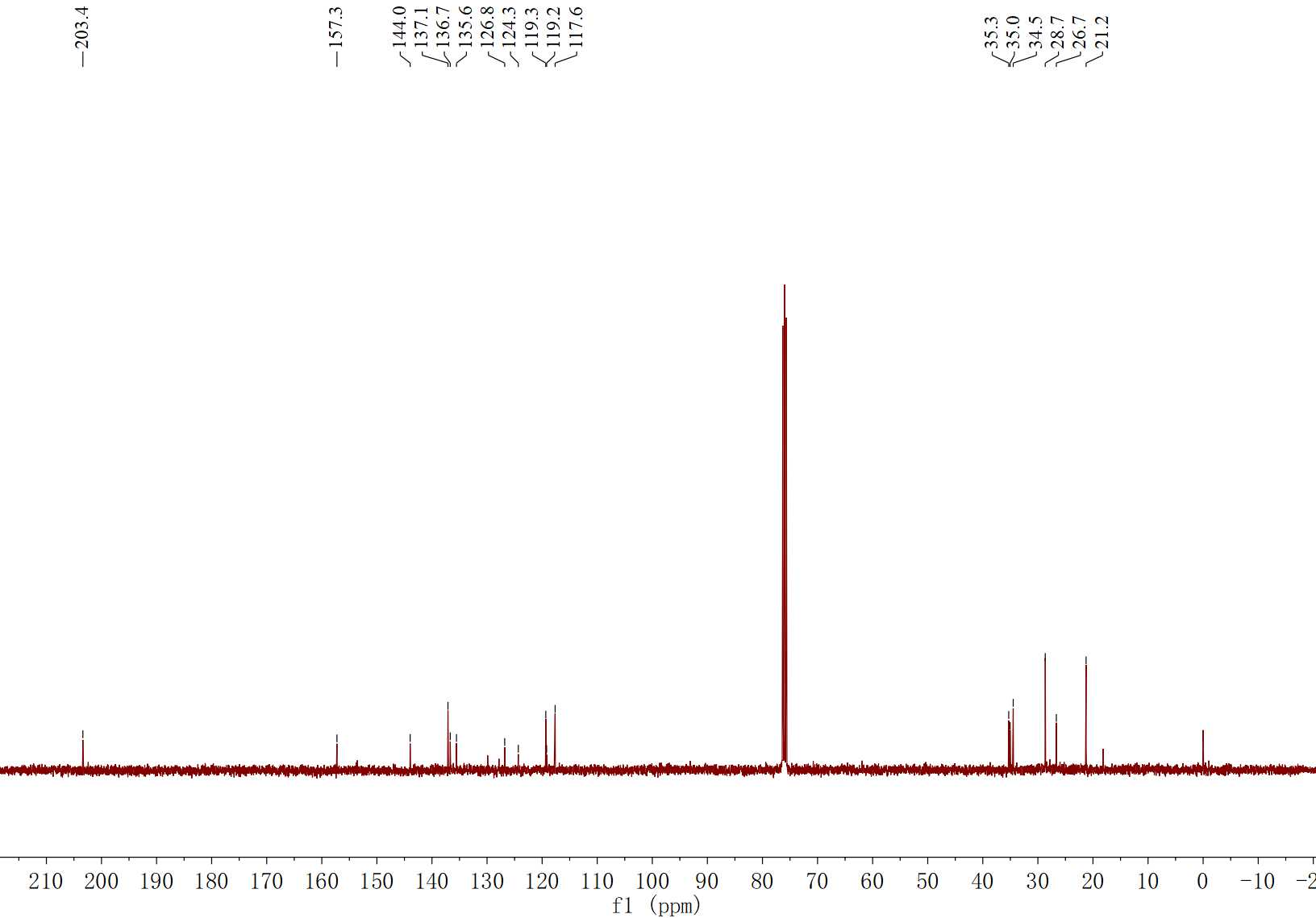
**

**Miltinone I: ^1^H NMR**

**
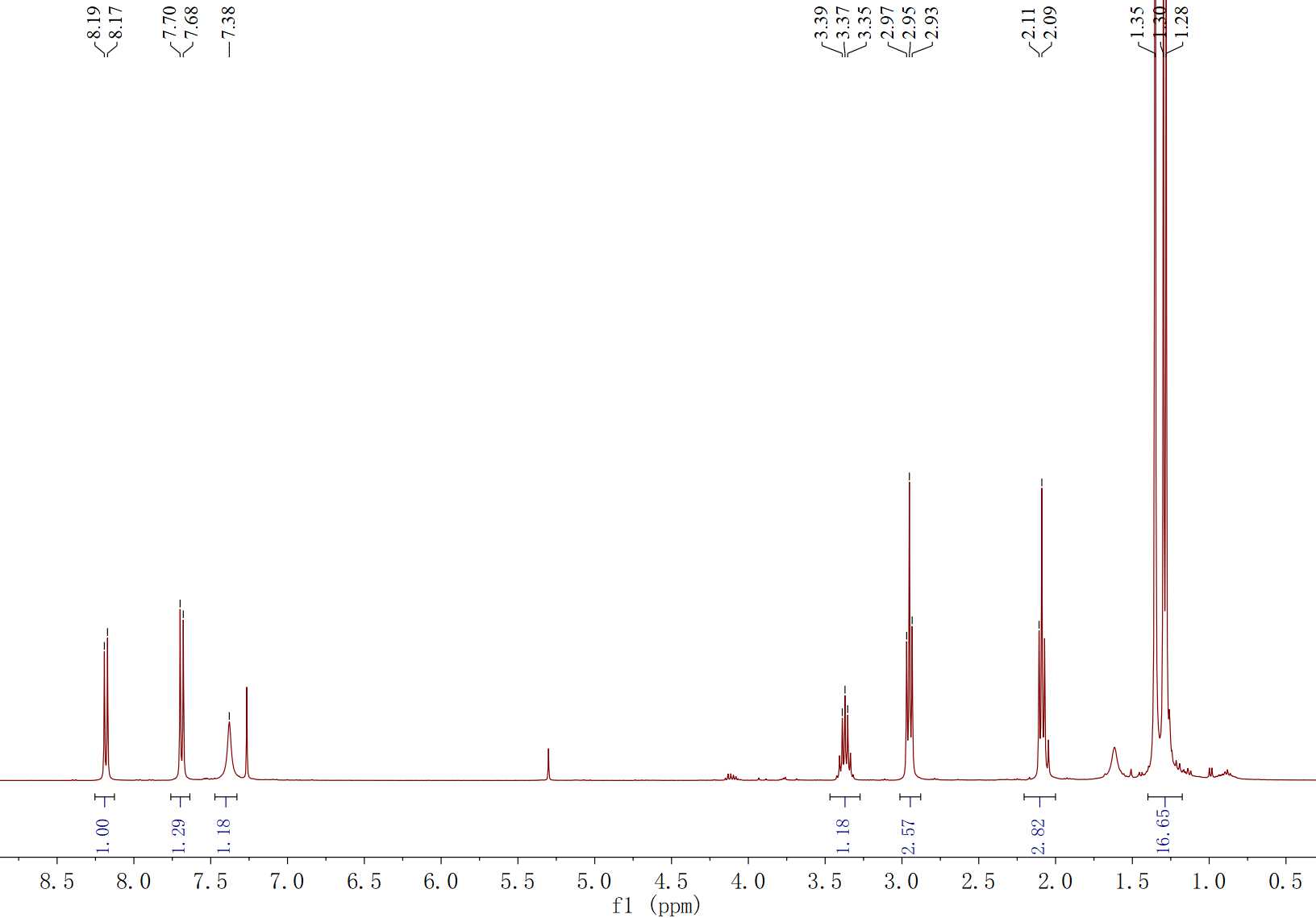
**

**Miltinone I: ^13^C NMR**

**
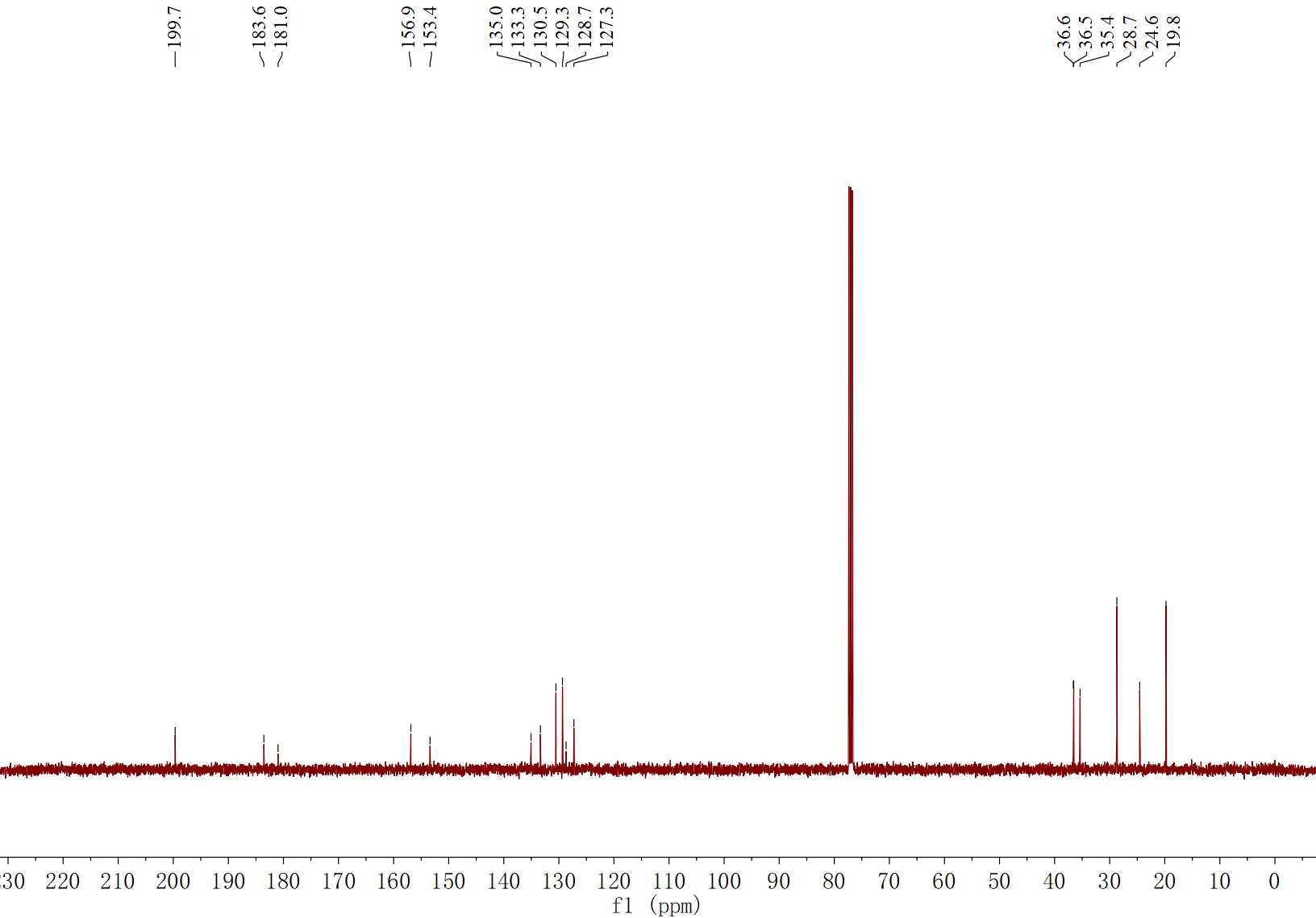
**

1. ^^ [↑](#footnote-ref-2)
2. ^^ [↑](#footnote-ref-3)
3. ^^Corresponding authors. e-mails: [qinhongbo@mail.kib.ac.cn](mailto:qinhongbo@mail.kib.ac.cn); [xugang008@mail.kib.ac.cn; xuhongxi88@gmail.com](mailto:xugang008@mail.kib.ac.cn; xuhongxi88@gmail.com).

   ^†^Both authors contributed equally to this work. [↑](#footnote-ref-4)
